# Pelvic and hindlimb osteology and ontogeny of a troodontid from the upper member of the Two Medicine Formation (Cretaceous) of Montana

**DOI:** 10.1371/journal.pone.0356249

**Published:** 2026-09-09

**Authors:** David J. Varricchio, Heath R. Caldwell

**Affiliations:** 1 Department of Earth Sciences, Montana State University, Bozeman, Montana, United States of America; 2 Department of Biological Sciences, North Carolina State University, Raleigh, North Carolina, United States of America; 3 Paleontology, North Carolina Museum of Natural Sciences, Raleigh, North Carolina, United States of America; Universiteit Maastricht, NETHERLANDS, KINGDOM OF THE

## Abstract

Troodontid theropod dinosaurs from the Campanian of western North America have historically been known from fragmentary remains. This limited material has made understanding the biology and taxonomy of this clade challenging. Here we describe the pelvic and hindlimb osteology for a troodontid from the Flagg Butte Member, the uppermost unit, of the Two Medicine Formation of western Montana, USA. This sample of embryonic to skeletally mature material provides a nearly complete picture of this part of the skeleton. In addition to providing details of anatomy for this taxon, information on individual variation and pathology can also be assessed. This material has historically been referred to as *Troodon formosus* and we follow the recent proposal to formally establish this taxonomic assignment. As is frequently reported in troodontid literature, many of the pedal elements for *T. formosus* in this sample are potentially pathologic. Overall element morphology is relatively consistent through ontogeny, with a majority of major skeletal landmarks being observable across the growth series. This observed invariance through growth in *Troodon* further constrains our understanding of intraspecific variation for troodontids and justifies the taxonomic uniqueness of potentially immature specimens (e.g., *Philovenator curriei*). The major ontogenetic changes that do occur (element robustness, skeletal fusion, surface texture) appear to happen at consistent growth stages, potentially reflecting low ontogenetic plasticity. The material described here provides additional opportunities for researching large-bodied troodontids in North America.

## Introduction

Troodontidae is an enigmatic clade of theropods that were widespread across the Northern Hemisphere from the Late Jurassic to the Late Cretaceous [1–7]. Nevertheless, the record of troodontids, especially in North America, is particularly sparse. This has been especially true for troodontines (sensu [8]) such as *Troodon formosus* [6,9], which has historically been known from isolated skeletal remains and resulted in a convoluted taxonomic history [6]. The specific taxonomic challenge for *Troodon formosus* is that the type specimen, an isolated tooth [9], is both of vague geographic and stratigraphic placement no more definitive than the Campanian of Montana [10] and remains currently undiagnostic at the species level [11–13]. Leidy [10] simply states the specimen was “obtained from the vicinity of the Judith River, one of the tributaries near the source of the Missouri River.” Further, in one individual troodontid, teeth are highly variable [3,14] and currently it is unclear how one could tackle diagnostic characters of teeth by species without complete dentitions for each taxon. Thus, the *T. formosus* type specimen only allows recognition of a troodontid in Montana during the Campanian [11–13]. Nevertheless, the name, *Troodon formosus*, has had common usage for forty years, especially for material from the Two Medicine Formation of Montana. Its usage persists in research papers and the public. For example, all seven publications focusing on troodontids from 2026 (up to the time of the submission of this manuscript), use *Troodon formosus* or *Troodon* in some form [15–21]. Consequently, there is a need for both a clear and stable definition of the taxon. Here, we follow the proposal of Varricchio et al. [6] and Hogan and Varricchio (In Review, Case 3917 with International Commission of Zoological Nomenclature [22]) to recognize MOR 553 as a neotype for *Troodon formosus*. This paper represents the first in a planned series of publications to describe the MOR 553 material and other *Troodon* specimens from the Two Medicine Formation in detail.

With the discovery and more detailed analysis of additional troodontid remains over the last 30 years, a clearer understanding of troodontid biology and ecology is emerging. Morphologic and isotopic data, for example, have been used to argue that some troodontids were omnivorous [23,24]. The discovery of nesting traces and egg clutches associated with embryonic and adult remains of *Troodon* have provided insight into parental care and reproduction for this group [25–29] and has prompted questions regarding how these animals engaged in such behaviors [30,31]. Although typically resolved as sister to Dromaeosauridae (e.g., [32]), there are some features of troodontids that are more similar to crown birds [33,34] and may suggest alternative relationships amongst this group of animals. Despite these advancements in troodontid research, the sparse fossil record has limited more precise interpretations for this group, especially for larger taxa such as *Troodon*.

The pelvic and hindlimb skeleton of troodontines is morphologically distinct from other theropods, including other troodontids [35–37]. A detailed understanding of the anatomy of this part of the skeleton will enable more nuanced interpretations of functional morphology for this group. Multiple osteologic features point towards cursoriality in troodontids, especially troodontines. The relative proportions and muscle attachment locations of the pelvis and hindlimb for troodontines are generally consistent with what is expected for cursorial animals [38,39]. Unlike dromaeosaurids, troodontids possess an arctometatarsalian or sub-arctometatarsalian metatarsus and a reduced fibula that does not articulate with the calcaneum [40]. These features have been argued to be advantageous for cursorial locomotion and pivoting [41,42]. The relative lack of ginglymoid pedal articulations in troodontids compared to dromaeosaurids may also be consistent with cursorial abilities in the former [43,44]. Analyses of trabecular bone orientation and musculoskeletal modeling also suggest that troodontids had a hindlimb posture that is intermediate between the more upright stance of large bodied theropods (e.g., tyrannosaurs) and more crouched stance of small extant birds (e.g., *Gallus*) [45,46]. Despite this extensive body of work, the complicated nature of biomechanics makes interpretations, especially from fragmentary remains, difficult [38]. A more complete sample of fossil material for a single troodontine is needed to address these questions.

Troodontid ontogeny is poorly understood. Numerous osteohistologic investigations of troodontids have been undertaken (e.g., [33,47–50]). However, an understanding of morphological changes through ontogeny is more limited, especially for troodontines. Only a few troodontid taxa, including *Troodon*, are known from multiple specimens [1,4,6]. This has resulted in some varied taxonomic interpretations of troodontid specimens over the years. For example, the potentially immature holotype specimen of *Philovenator curriei* [51] was initially interpreted as a juvenile specimen of *Saurornithoides mongoliensis* [52]. Similarly, perinate troodontid material initially assigned to *Byronosaurus* [53] is now considered to represent a distinct taxa [54]. While these more recent taxonomic interpretations are likely not problematic, an ontogenetic series for a single troodontine taxon would help clarify these interpretations.

Here we provide a detailed description of the pelvic and hindlimb osteology for a collection of *Troodon formosus* material from the Upper Cretaceous Two Medicine Formation of Montana. The specimens described here include associated remains from embryonic to skeletally mature, as well as multiple individuals from a bonebed. These specimens provide a rare opportunity for understanding the complete osteology and individual variation for a historically enigmatic theropod.

## Methods

### Taxonomy

We follow the rationale of Varricchio et al. [6] and the proposal that the material from Jack’s Birthday Site, MOR 553, in the Two Medicine Formation of Montana should be adopted as the neotype for *Troodon formosus*. The official proposal, Case 3917 [22], is currently under review. The justification for this revision is primarily an argument for stability. The name *Troodon formosus* has a forty-year history of use broadly in the Campanian formations of the Alberta-Montana region of Laramidia as well as specifically in the Two Medicine Formation beginning with Currie [14] and Horner [55]. The name has been extensively used within scientific literature with at least 327 references between 1856 and 2024 and the name and dinosaur are widely known among the public as well (161 references between 1986 and 2020) [22]. Hence, there is value in preserving the name from both a scientific and outreach perspective, and this description provides part of the necessary anatomical foundation.

Although the original type specimen for *T. formosus*, a single tooth (ANSP 9259) (Leidy [9]) has proved problematic at the species level [8,11,56] it remained sufficiently diagnostic to eventually demonstrate the presence of a troodontid in the Campanian of Montana and Alberta. Currie [14] formalized the use of *T. formosus* and this species name was applied to troodontid material in the Two Medicine Formation of Montana and elsewhere in the region. More recently, van der Reest and Currie [8] proposed abandonment of *T. formosus* and a return to *Stenonychosaurus inequalis* as a name. This proposal has two major flaws. First, its type specimen, a crushed and weathered foot [57,58] is equally undiagnostic as Leidy’s tooth. Second, the name has had little use in comparison to *T. formosus*. Hence, Varricchio et al. [6] and Hogan and Varricchio [22] propose a neotype of MOR 553 that addresses the anatomical issue with a sample of most skeletal elements including ontogenetic variation while preserving a name broadly used by both researchers and the public.

Van der Reest and Currie ([8], p. 934) suggest that any material to be identified as *T. formosus* must come from the Judith River Formation—the type horizon for *T. formosus*. However, Art 75.3.6 states that a proposed neotype need only originate from as close as practicable to the type locality and horizon [59]. Given the absence of any precise locality data for the tooth, the Two Medicine Formation is a reasonably practical solution. The Two Medicine and Judith River Formations of Montana are time equivalent, comprise one sedimentary wedge and represent portions of the same coastal plain back in the Cretaceous [60]. Teeth from the Two Medicine and specifically from MOR 553 broadly overlap morphologically with the type specimen and have been conflated in some recent analyses [12,13,61]. Importantly, in establishing a neotype, the type locale would now be that of Jack’s Birthday Site in the Two Medicine Formation, a site well placed stratigraphically and chronologically [60].

Petitions to the ICZN can take years to be resolved; e.g., Case 3506 for the neotype designation of *Allosaurus fragilis* [62] took 13 years before an official opinion was reached [63]. Here we treat MOR 553 as the presumed neotype. But by providing a detailed osteologic and ontogenetic descriptions of this Two Medicine troodontid material, we hope to facilitate any needed nomenclatural decisions once Case 3917 is ultimately resolved.

Hogan and Varricchio [22] consider *Stenonychosaurus inequalis* to be a junior synonym of *Troodon formosus*. Though *Latenivenatrix mcmasterae* could prove to be synonymous with *Troodon* [64], we treat it as a potentially distinct taxon. We use ‘*L. mcmasterae*’, following Cullen et al. [64], to refer to morphotypes assigned to this taxon by van der Reest and Currie [8].

### Materials

**Fig 1:**
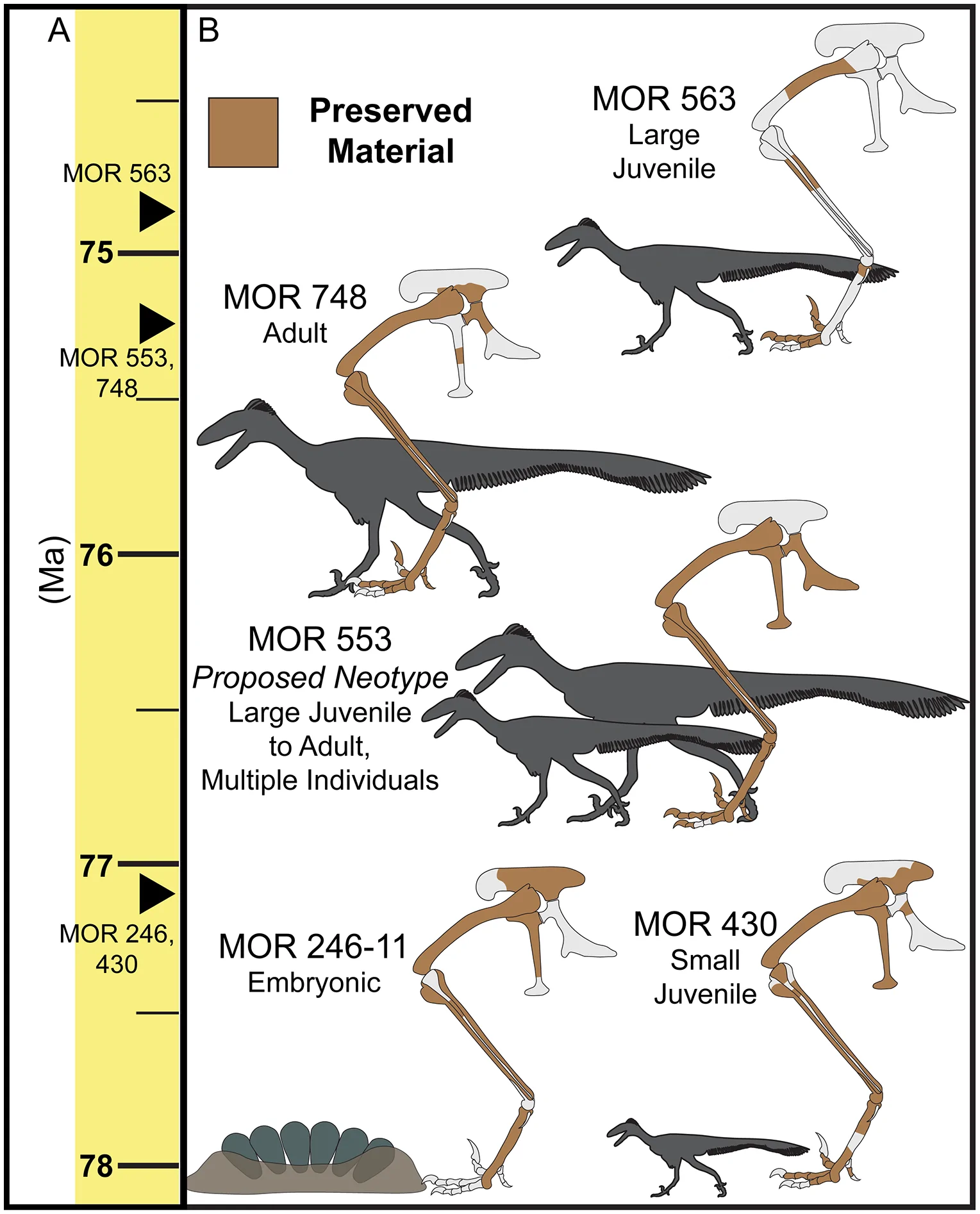
Geologic context and preserved material for *Troodon* described here. Chronologic placement of Two Medicine *Troodon* specimens after Rogers et al. [60] (A). Graphical representation of preserved pelvic and hindlimb material, with silhouettes reflecting relative size of the individuals preserved (B). Abbreviation: Ma, millions of years ago. *Troodon* silhouettes made with reference to work by Scott Hartman (https://creativecommons.org/licenses/by-nc-sa/3.0/).

Descriptions are based on five Museum of the Rockies (MOR) specimens from the Campanian Two Medicine Formation of Montana. Across these specimens, nearly the entire pelvic and hindlimb skeleton is preserved: MOR 246−11, a partially exposed embryonic skeleton [27,65]; MOR 430, a partially complete small juvenile skeleton; MOR 563, a partially complete large juvenile skeleton; MOR 748, a partial adult skeleton; and MOR 553 (Jack’s Birthday Site) a multi-taxic bonebed containing multiple disarticulated *Troodon* individuals ranging from large juvenile to adult (Fig 1). The material described here from MOR 553 can be confidently assigned to *Troodon* based on comparisons to other troodontid specimens and the close association of elements from the locality [43]. Additional fragmentary specimens (S1 File) are also evaluated.

It is worth noting that MOR 246 and 430 are stratigraphically separated from and bear some anatomical features (see below) that distinguish them from the other Two Medicine troodontid specimens. Whether or not MOR 246 and 430 are taxonomically distinct from the other Two Medicine specimens is the subject of future study.

### Stratigraphy/locality

The specimens described here come from either Teton (MOR 246, 430) or Glacier (MOR 558, 563, 748) County, Montana. The two ontogenetically youngest specimens, the embryonic MOR 246 and small juvenile, MOR 430, come from nearly stratigraphically equivalent and geographically close (~1.4 km) locales: Egg Island (MOR locality TM-024) and Egg Mountain site (TM-006). Both sit near the top of a 10- to 15-meter sequence dominated by lacustrine deposition [66]. Stratigraphic placement of this lacustrine carbonate interval (LCI) suggests a date of approximately 77–76.5 Ma (Rogers et al. [60], fig. 15). The large juvenile through adult specimens, MOR 563, MOR 553, and 748, come from higher in the formation. MOR 553 and MOR 748 also occur both close geographically and stratigraphically (TM-068), sitting 12.5 and 14 meters, respectively, above the 90TMT-590-U60 bentonite of Ramezani et al. [67] and Rogers et al. [60]. MOR 563 comes from an isolated patch of outcrops with a less precise stratigraphic position (locality TM-071). However, the associated lithologies are more consistent with a position above MOR 748 and 553 [60].

All the specimens come from the upper Two Medicine Formation, recently recognized as the Flag Butte Member [60]. This unit correlates to and encompasses the entirety of both the upper, fossil-producing portion of the Judith River Formation, the Dinosaur Park Formation, and likely at least portions of the Oldman Formation [60,67]. Recent radiometric dates reflect these relationships, with the Flag Butte Member dating to 76.99–74.78 Ma (Rogers et al. [60], fig. 15). Whereas embryonic and small juvenile specimens (MOR 546, 430) occur early in this range, the large juvenile and adult material (MOR 553, 563, 748) represent a much smaller interval (< 0.5 million years) closer to 75 Ma. In total, the five specimens represent approximately 1.5 million years of the Flag Butte Member. The larger, ontogenetically older Two Medicine specimens span the most likely source of the type specimen from the Judith River and the ranges of “Stenonychosaurus inequalis” and “L. mcmasterae” as reported by van der Reest and Currie [8].

### Taphonomy

Many of the specimens described here have undergone some form of taphonomic deformation. This is especially prevalent in some specimens from the MOR 553 locality. Most of the small elements from MOR 553 are relatively undeformed. Larger elements variably exhibit taphonomic deformation near the epiphyses, which can be crushed or telescoped onto the relatively undeformed diaphysis. This deformation reflects the lithostatic compaction of the matrix after burial and varies by the horizontal through vertical range of orientations in which the bones were found [68]. MOR 430 also has some bones that exhibit some taphonomic flattening or distortion. MOR 563 and 748 present some taphonomic alteration of the cortical bone, likely corroded through soil processes.

### Ontogeny

The relative growth stages of these *Troodon* specimens can be confidently inferred based on osteohistology [47,49,50,69,70] and the relative degree of skeletal co-ossification [71]. MOR 246−11 is an embryonic individual [27]. MOR 430 represents an individual well under a year in age [69]. MOR 563 and other large juveniles of MOR 553 represent individuals approximately 1 year old [50,71]. Larger individuals (subadults through adults) of MOR 553 and 748 are three to perhaps eighteen years old [47,50,69]. MOR 748 being found associated with a clutch of eggs [70] is additional evidence that individuals of this body size are sexually mature.

### Allometric analysis

Allometric analyses for selected individual skeletal elements and inter-element relationships in the *Troodon* sample were conducted to assess changes in element shape and limb proportions through ontogeny. For inter-element comparisons, limb bone lengths were regressed against the anteroposterior diameter of the femur midshaft, a proxy for body mass [72]. Linear measurements (e.g., length and width) were log-transformed and used to generate least-squares linear regressions with 95% confidence intervals. For the intra-element analyses, relationships with scaling coefficients that were significantly greater than 1 were classified as positively allometric whereas elements with scaling coefficients not significantly different from 1 were considered isometric (S1 File). Given the small sample sizes (n = 3) for inter-element analyses, no statistical tests were conducted, and scaling relationships were classified based on the value of the scaling coefficient alone (e.g., greater than or less than 1). It is important to note that, while specimens with obvious taphonomic deformation were excluded from this analysis, more subtle taphonomic factors may be influencing these results.

### Institutional abbreviations

**AMNH**, American Museum of Natural History, New York City, New York, USA. **ANSP**, Academy of Natural Sciences of Drexel University, Philadelphia, Pennsylvania, USA; **CMN**, Canadian Museum of Nature, Ottawa, Canada; **DLXH**, Dalian Xinghai Museum, Dalian, China; **DNHM**, Dalian Natural History Museum, Dalian, China; **IGM**, Geological Institute of the Mongolian Academy of Sciences, Ulaan Bataar, Mongolia; **IVPP**, Institute of Vertebrate Paleontology and Paleoanthropology, Bejing, China; **LH**, Long Hao Institute of Geology and Paleontology, Hohhot, Inner Mongolia, China; **MOR,** Museum of the Rockies, Bozeman, Montana, USA; **MPC-D**, Mongolian Paleontology Center, Ulaan Bataar, Mongolia; **UALVP**, University of Alberta Laboratory of Vertebrate Paleontology, Edmonton, Alberta, Canada; **WYDICE,** Wyoming Dinosaur Center, Thermopolis, Wyoming, USA; **ZPAL**, Institute of Paleobiology, Polish Academy of Sciences, Warsaw, Poland.

## Results

### Systematic paleontology

Theropoda Marsh, 1881 [73]

Maniraptora Gauthier, 1986 [74]

Troodontidae Gilmore, 1924 [75], sensu Turner et al., 2012 [32]

Troodontinae Gilmore, 1924 [75], sensu van der Reest and Currie, 2017 [8]

*Troodon* Leidy, 1856 [9]

Type species

*Troodon formosus,* Leidy, 1856 [9], by original designation from the Judith River Formation of central Montana, U.S.A. [9,76] pl. 9, figs. 53–55).

*Troodon formosus,* Leidy, 1856 [9]

### Holotype

Tooth (ANSP 9259) from the Judith River Formation, Montana, U.S.A. by original designation ([9,76] pl. 9, figs. 53–55).

### Proposed neotype

Disarticulated bonebed remains (MOR 553) from the Two Medicine Formation, Montana, U.S.A. consisting of the skeletal remains of multiple individuals from large juvenile to adult [6]. See Hogan and Varricchio [22] for a complete list of materials.

### Diagnosis (from Varricchio et al. [6])

A troodontid within Troodontinae that is distinguished from other troodontids by possessing a maxilla with anteriorly, a larger, more broadly rounded maxillary fenestra, a low-angled nasal process with a stepped anterior portion, bearing 23 teeth, and having a large palatal shelf extending posteriorly along the midline to the posterior limit of the maxillary fenestra; more pronounced basioccipital tubera [77]; an L-shaped to triangular frontal with a flat, shallowly anteroposteriorly rippled nasofrontal contact [8].

### Expanded diagnosis (specific to the pelvic girdle and hindlimb)

*Troodon formosus* can also be distinguished from other troodontines from a unique combination of characters in the pelvic girdle and hindlimb.

An ilium (MOR 246−11, 430) with a ventrally sloping dorsal margin of the postacetabular process (shared with *Almas ukhaa* [54] and *‘L. mcmasterae’* – UALVP 55804 [8]). For adults, a pubis (MOR 553s-8-3-9-387) with a pubic boot that extends further anteriorly than posteriorly (shared with *Almas ukhaa* [54]), but see below. An ischium (MOR 553l-7-27-8-87) that tapers to an acute point distally (shared with *Saurornithoides mongoliensis* [3]), has a relatively small obturator process (shared with *Saurornithoides mongoliensis* [3] and *Gobivenator mongoliensis* [35]) with a deep notch immediately posterior to it (shared with *Almas ukhaa* [54] and *Gobivenator mongoliensis* [35]). A femur (e.g., MOR 553s-7-2-91-239, 748) lacking a fourth trochanter (shared with *Talos sampsoni* [56] and *Saurornithoides mongoliensis* [3]), and a medial condyle that is slightly wider than the lateral condyle (shared with *Gobivenator mongoliensis* [35]). A cnemial crest of the tibia that (e.g., MOR 553s-8-16-92-254, 748) is rounded with an apex at midheight (shared with *Almas ukhaa* [54] and *Harenadraco prima* [78]). An astragalus (e.g., MOR 553s-7-7-91-19, 748) with a nearly straight transverse groove at the base of the ascending process, a craniodorsal groove on the medial condyle, a notch between the lateral condyle and the ascending process, and a medial condyle that extends slightly proximal to the base of the ascending process [56]. A relatively short metatarsus (e.g., MOR 748, shared with *Linhevenator tani* [79]). Metatarsal III (e.g., MOR 8-6-9-406, 748) with an asymmetrical tongue-like projection of the distoposterior articular surface (shared with *Urbacodon* [80], *Talos sampsoni* [56], and specimens attributed to *Stenonychosaurus inequalis* – e.g., NMC 8539 [58]), and a shaft with a flat to convex anterior face (shared with *Talos sampsoni* [56] and specimens attributed to *Stenonychosaurus inequalis* – e.g., TMP 1998.068.0090 [8]) and triangular to oval-shaped extensor fossa (shared with *Talos sampsoni* [56], *‘L. mcmasterae’* – e.g., TMP 1997.133.0008 [8], and specimens attributed to *Stenonychosaurus inequalis* – e.g., TMP 1998.068.0090 [8]). Metatarsal IV with a dorsolateral groove on the distal end that is not closed off distally [56]. Pedal phalanx I-1 (e.g., MOR 553s-7-21-92-45, 748) with a proximally placed pit on the lateral surface (shared with *Talos sampsoni* [56]).

### Description

#### Ilium.

**Fig 2:**
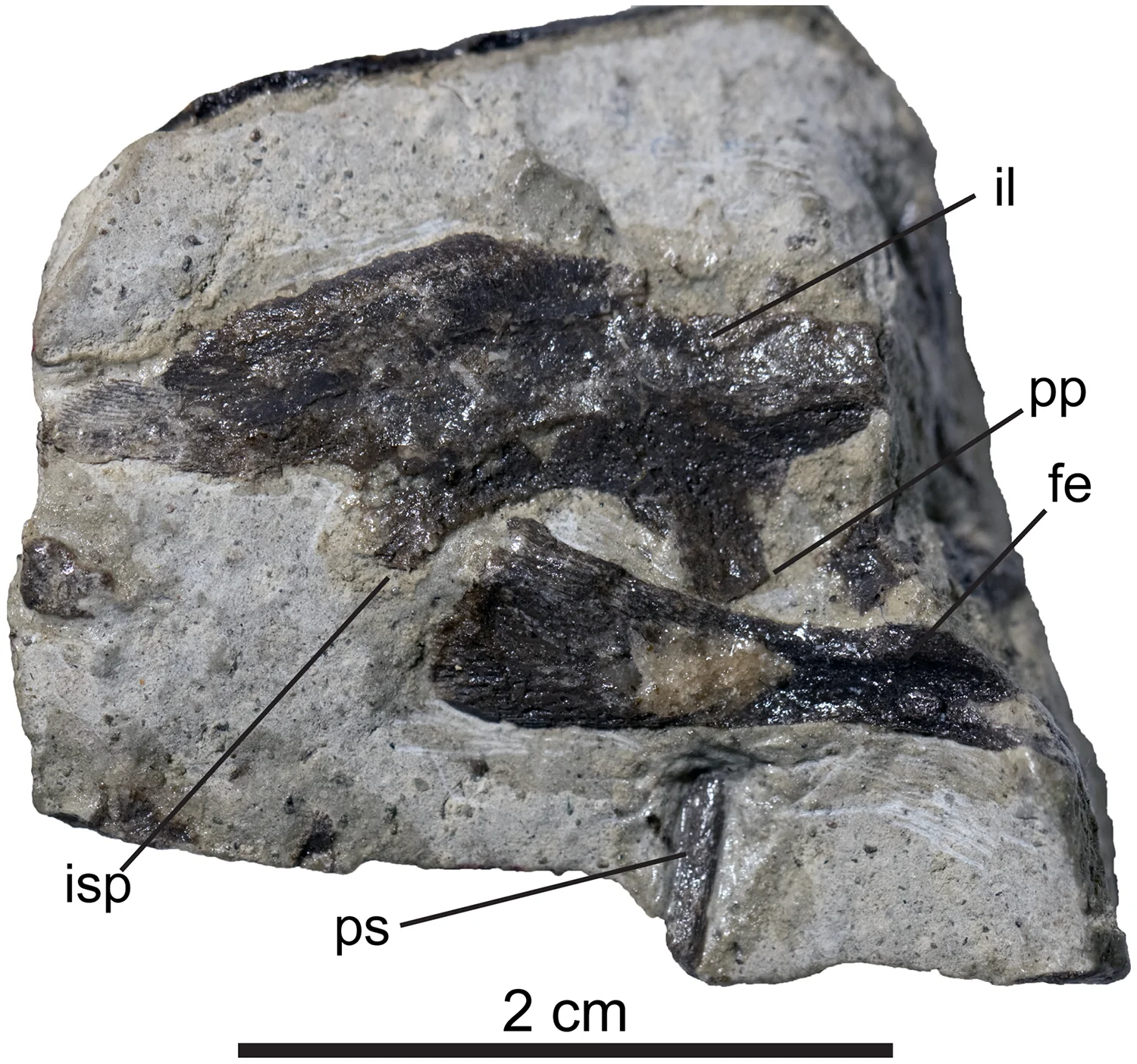
Embryonic pelvis and femur of *Troodon formosus.* Embryonic pelvic elements for *Troodon* (MOR 246−11) in lateral view. Abbreviations: fe, femur; il, ilium; isp, ischiadic peduncle; pp, pubic peduncle; ps, pubis.

Only partial ilia are known for specimens of *Troodon* from the Two Medicine Formation (MOR 246−11, MOR 430, MOR 553 (float), MOR 563, MOR 748). By far the best specimen is that from MOR 246−11, which contains the right ilium, femur, and pubis in articulation (Fig 2). The egg has split so that the exposed fracture cut through the ilium and femur in a parasagittal plane. This affords a view of the overall shape, but not of any surface features. The anterior end is not observable, remaining buried within the matrix. The ilium of MOR 246−11 is long and low, the dorsal-ventral height being shorter than the acetabular region (from anterior of pubic peduncle to posterior of ischiadic peduncle). The acetabulum is anteroposteriorly longer than dorsoventrally deep. The dorsal margin remains relatively horizontal, showing a slight dip above the acetabulum then posterior to ischiadic peduncle it slopes ventrally. The post-acetabular region is nearly as long as the acetabular region, and tapers to a rounded point. The pubic peduncle is longer antero-posteriorly and extends slightly farther ventrally than the ischiadic peduncle.

MOR 748 preserves a few isolated fragments of the ilium, including: one piece spanning the left acetabular region; two fragments of the acetabulum from the right side; and two pieces that are mirror images of each other, possibly from the iliac blade. The acetabular portions show that the ilium’s contribution to the acetabulum faces ventrally and is fairly broad. The blade fragments may represent the postero-dorsal margin of the ilium. Both bear on presumably their dorsal edge a roughened and fattened muscle scar. These pieces each measure ~22 mm long and ~7–9 mm wide.

**Fig 3:**
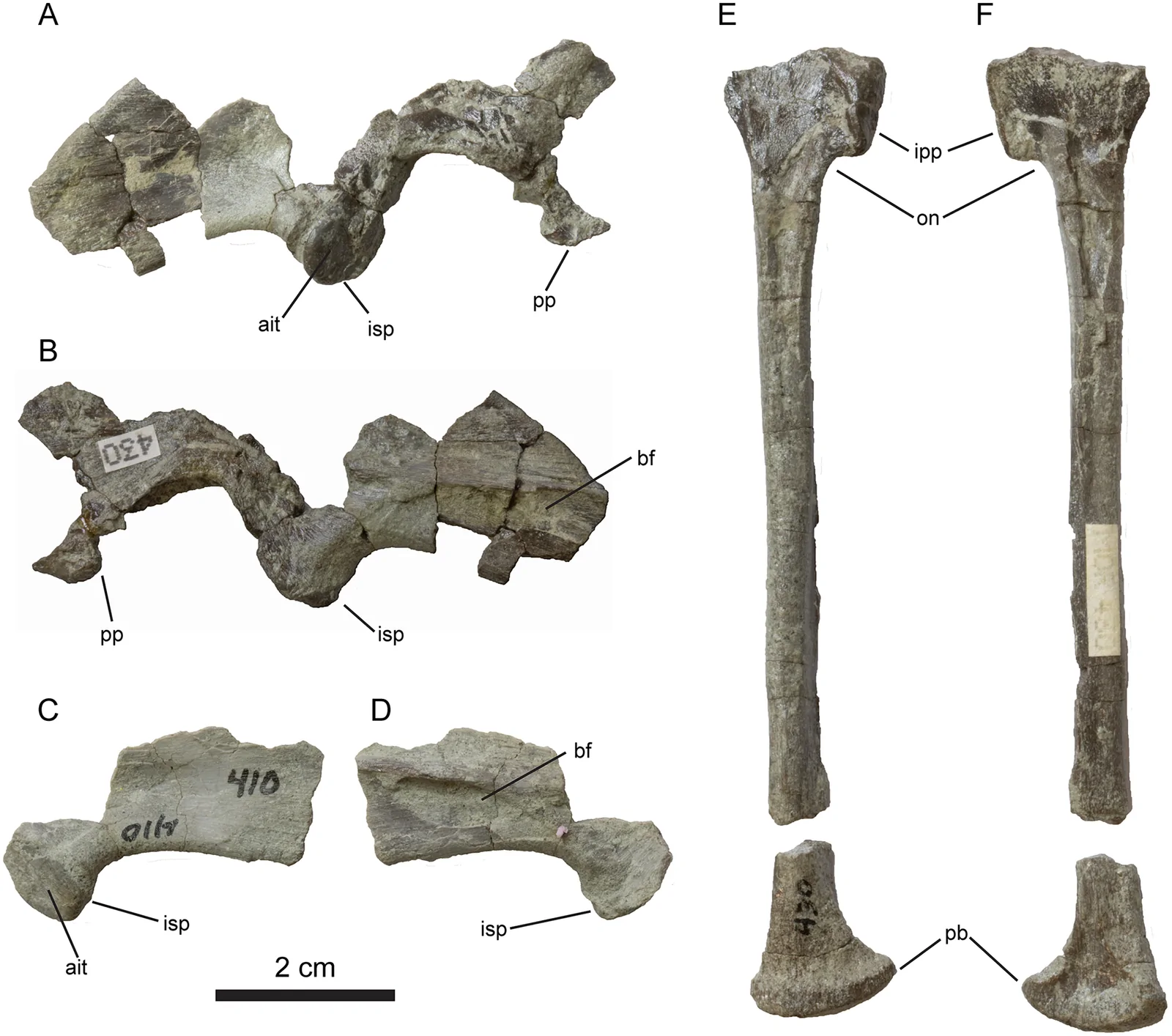
Ilium and pubis of small juvenile *Troodon.* Pelvic elements of *Troodon* (MOR 430). Right ilium in lateral (A) and medial (B) views. Left ilium in lateral (C) and medial (D) views. Left pubis in lateral (E) and medial (F) views. Abbreviations: ait, antitrochanter; bf, brevis fossa; ipp; ischiadic process of pubis; isp, ischiadic peduncle; on, obturator notch; pb, pubic boot; pp, pubic peduncle. Note: Some portions of MOR 430 were originally catalogued as part of MOR 410.

MOR 430 also preserves portions of both ilia, with some fragments originally being catalogued as MOR 410 (Fig 3). These pieces preserve the post-acetabular region and the ischiadic peduncle. The right ilium also includes the complete acetabular portion and some of the pubic peduncle. The preserved outline matches that of the embryo (MOR 246). The medially directed horizontal brevis shelf runs the length of the post-acetabular portion about mid-height and divides this portion into subequal dorsal and ventral halves. This shelf’s medial edge hangs down slightly and helps define a slightly concave ventrally located depression – the brevis fossa. A small concavity sits just dorsal to the ischiadic peduncle on the medial side of the ilium. Laterally, the blade of the ilium is concave in the preserved post-acetabular region. The right ilium of MOR 430 preserves the acetabular region fairly well. The ilium’s contribution to the acetabulum begins with a slightly concave surface. As it arches upwards it becomes strongly concave and remains so through its apex. It then rapidly shifts to a planar surface rotated laterally. The surface also widens. Thus, the posterior portion of the acetabulum- the antitrochanter- is a broad antero-laterally and ventrally directed planar surface. The entire surface has a smooth glossy finish, indicating it was covered in life with articular cartilage. The contact for the ischium appears to be relatively small and rounded, though this may reflect the immaturity of the specimen.

#### Ilium comparisons.

The overall profile of the ilium (MOR 246−11, 430) for *Troodon* is similar to other troodontids, with a dolichoiliac shape [8,81,82] and subequal lengths of the acetabular and postacetabular regions [1,8,35,83]. Based on the embryonic specimen (MOR 246−11), *Troodon*, at least early in ontogeny, has an estimated ilium/femur length ratio (~0.82) that is similar to *Almas* (0.83) [54] and *Liaoningvenator* (0.72) [83], but greater than *Mei* (0.53) [1] and other early troodontids [84–86] (Table 1).

**Table 1:** Pelvic and hindlimb length proportions for troodontids.

Taxon	Specimen	Femur Length (mm)	Ilium/Fe	Pubis/Fe	Ischium/Pubis	Fe/Tibia	MT III/Fe
Troodon	MOR 246−11	33.8	0.82^E^	–	–	–	–
Troodon	MOR 430	126	–	0.82	–	0.72^E^	–
Troodon	MOR 748	320	–	–	–	0.89	0.66
Troodon	MOR 553s	218-330^E^	–	–	0.77^E^	–	–
Philovenator	IVPP V10597	86.5	–	–	–	–	1.24
Mei	IVPP V12733	81	–	–	–	0.8^E^	0.72
Mei	DNHM D2154	65^E^	0.53	–	–	0.8^E^	0.75
Liaoningvenator	DNHM D3012	109	0.68	–	–	0.675	–
Daliansaurus	DNHM D2885	130.8	0.63	–	–	0.69	–
Sinornithoides	IVPP V9612	140	0.52	0.64	0.53	0.75^E^	0.79
Almas	IGM 100/1323	68.3	0.83	0.88	0.63	0.75^E^	–
Gobivenator	MPC-D 100/86	192	–	0.88	0.54	–	0.83
Linhevenator	LH V0021	240^E^	–	–	–	–	0.63^E^
Jianianhualong	DLXH 1218	116.5	0.645	0.78	0.48	–	0.725
Sinovenator	IVPP V12615	–	–	–	0.40	–	–
Sinusonasus	IVPP V11527	–	–	–	0.54	–	–

Pelvic and hindlimb length proportions for *Troodon* and other troodontids [1,35,37,52,54,79,83–88]. Abbreviations: ^E^estimated value, Fe, femur; -, no data.

The ventrally sloping dorsal margin of the postacetabular process for *Troodon* (MOR 246−11, 430) is present in *Almas* [54], *Jianianhualong* [86], *Dalianasaurus* [85], and ‘*L. mcmasterae’* (UALVP 55804) [8], *Buitreraptor* [89], *Anchiornis* [82], and dromaeosaurids such as *Velociraptor mongoliensis* [90] and *Microraptor* [91], but contrasts with the nearly horizontal dorsal margin of *Mei* [1] and *Gobivenator* [35] and concave dorsal margin of *Rahonavis* [92] and unenlagiines [89,93].The horizontal ventral margin of the postacetabular process (MOR 246−11, 430) is shared with most troodontids [1,8,35,37,54], which is unlike the ventral deflection in the troodontid *Daliansaurus* [85] as well as *Anchiornis* [82], unelagiines [89,93], many dromaeosaurids [36,91,94,95], and various Mesozoic birds [93,96]. The posteriorly rounded postacetabular process in *Troodon* is shared with *Mei* [1] *Daliansaurus* [85], *Gobivenator* [35], and many non-troodontid paravians (e.g., [82,90–94,96,97],but is unlike the subvertical surface in ‘*L. mcmasterae*’ (UALVP 55804) [8]. The concave lateral surface of the iliac blade in *Troodon* (MOR 430) is also present in *Mei* [1] *Gobivenator* [35] and dromaeosaurids such as *Velociraptor mongoliensis* [90] and *Shri devi* [36]. The ilium for *Troodon* (MOR 430) lacks the pronounced lateral flaring present in *Mei* [1]. The greater distal extent of the pubic peduncle in *Troodon* is shared with *Sinornithoides* [81], *Gobivenator* [35], ‘*L. mcmasterae*’(UALVP 55804) [8], and most other paravian dinosaurs (e.g., [36,91,97]). *Troodon* and *Almas* both possess a concavity dorsomedial to the ischiadic peduncle [54]. The laterally placed antitrochanter on the ischiadic peduncle in *Troodon* (MOR 430) is shared with *Sinornithoides* [84] and the dromaeousaurids *Mahakala* [98] and *Velociraptor* [90], but contrasts with the more dorsally-located antitrochanter in *Daliansaurus* [85]. The supracetabular rim in *Troodon* (MOR 430) is shared with other paravian dinosaurs [35,81,90,98], but is more extensive in *Mei* [1] and *Anchiornis* [7].

### Pubis

**Fig 4:**
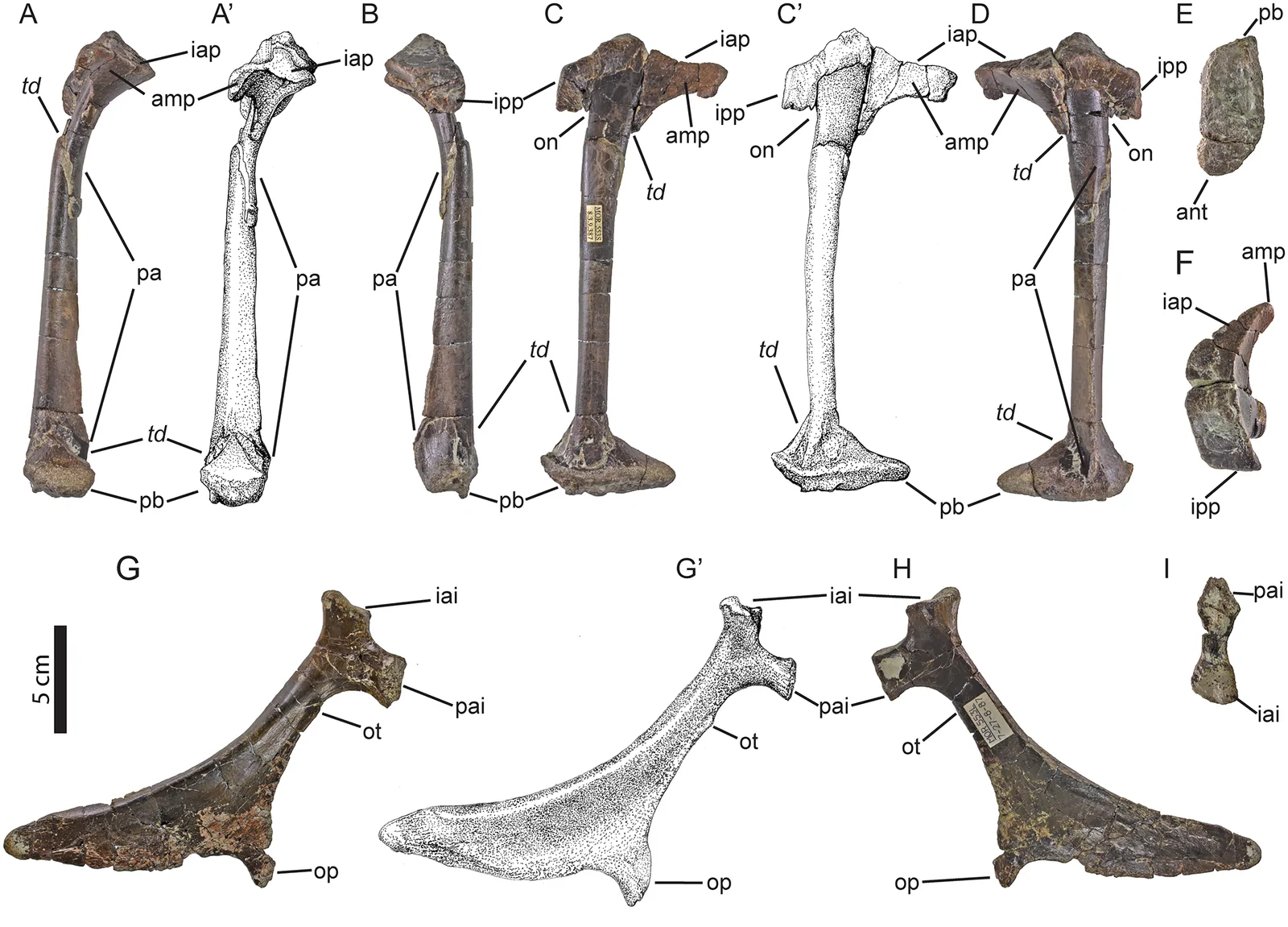
Pubis and ischium of adult *Troodon formosus.* Right pubis (MOR 553s-8-3-9-387) of *Troodon* in anterior (A), posterior (B), lateral (C), medial (D), distal (E), and proximal (F) views. Right ischium (MOR 553l-7-27-8-87) in lateral (G), medial (H), and proximal (I) views. Corresponding line drawings (A’, C’, G’). Abbreviations: amp, ambiens process; iai, iliac articulation of ischium iap, iliac articulation of pubis; ipp, ischiac process of pubis; on, obturator notch; op; obturator process; ot, obturator tubercle; pa, pubic apron; pai, pubic articulation of ischium; pb, pubic boot; *td*, taphonomic deformation.

A much better sample of *Troodon* pubes exist. The sample includes possibly an embryonic pubis from MOR 246−11 (Fig 2), a nearly complete as well as partial pubis from MOR 430 (Fig 3), a complete adult pubis (Fig 4) and partial juvenile pubis from MOR 553, and a shaft fragment from the adult MOR 748.

The proximal end of the pubis (MOR 553s-8-3-9-387) is concave laterally and convex medially. The ovoid ischiadic process is posteriorly facing, parallel to the shaft of the pubis, and perpendicular to the longer iliac articulation. The iliac articulation makes up a majority of the proximal end. The relative orientation of the articular surfaces (in addition to the orientation of the pubis in the articulated embryo of MOR 246−11) suggests that the pubis was oriented nearly vertically in life. The acetabulum sits between these articular surfaces, slightly longer than the ischial articulation and gently concave. Anteriorly, the ambiens process tapers and curves laterally. This contributes to the concave lateral side of the proximal end. An obturator notch is present adjacent to the ischiadic articulation.

The pubis for *Troodon* (MOR 553s-8-3-9-387) has a straight shaft with an oval cross-section for its approximate upper third and becomes more triangular in cross section distally. The pubic apron begins about one third of its length from the proximal end and increases in height distally. No evidence of fusion along the pubic apron is present. In MOR 553s-8-3-9-387, the pubis is weakly concave behind the pubic apron. A flat, striated, and posteromedially facing surface marks the shaft just posterior to the proximal portion of the pubic apron. The otherwise strait shaft arcs medially at its distal end.

Distally, the pubic boot from a presumably adult individual (MOR 553s-8-3-9-387) contains a small posterior projection and a large anterior projection. This specimen exhibits telescoping of the shaft and this taphonomic modification may have accentuated the asymmetry of the anterior/posterior projections. This contrasts with the condition that can be observed in the small juvenile individual, MOR 430, where the boot extends further posteriorly than anteriorly. It is possible that the boot of MOR 430 could be reversed from its true position, although the shaft and boot pieces appear to correspond to each other in cross section in the current articulation. It appears most likely that this indicates a shift in ontogeny where the anterior portion of the boot grows faster than the posterior portion. The pubic boot is medially flat to concave in MOR 430 and flat in MOR 553s-8-3-9-387, where this surface would contact the boot of the opposite pubis. MOR 246–11 also lacks a pubic boot, but the presence of porous bone and an extremely hollow shaft suggests that the pubic boot may not have been ossified at this early ontogenetic stage. In MOR 553s-8-3-9-387, bone on the dorsal surface of the pubic boot bears irregular grooves suggesting some type of soft tissue attachment. The ventral surface of the boot is slightly convex overall, bears irregular pits and bumps, and on a fine scale has a grainy finish. The surface contrasts sharply from the smooth bone of the shaft and resembles that of the ilium and ischium contacts.

### Pubis comparisons

The relative length of the pubis for *Troodon* is largely consistent with that of other troodontids with a pubis length to femur length ratio of 0.82 in MOR 430 (Table 1). This is similar to that of *Almas* (0.88) [54], *Gobivenator* (0.88) [35], and *Jianianhualong* (0.78) [86], but is greater than *Sinornithoides* (0.64) [84].

The laterally concave and medially convex proximal end of the pubis in *Troodon* (MOR 553s-8-3-9-387) contrasts with ‘*L. mcmasterae’* (laterally flat, medially convex) (UALVP 55804) [8], and *Mei* (concave laterally and medially) [1]. The orientations of the ischial and iliac articulations and vertical pubic shaft for *Troodon* (MOR 430, 553s-8-3-9-387) is shared with *Saurornithoides* [3], *Gobivenator* [35], and ‘*L. mcmasterae*’ (UALVP 55804) [8]. This is unlike the posteroventrally oriented pubis of *Sinornithoides* [84], *Sinovenator* [37], and dromaeosaurids such as *Velociraptor mongoliensis* [99]. The ovoid ischial articulation in *Troodon* (MOR 553s-8-3-9-387) contrasts with the triangular one in ‘*L. mcmasterae’* (UALVP 55804) [8]. The anteroposteriorly expanded articulation for the ilium (which presumably joins with the ilium to form the ambiens process) in *Troodon* (MOR 553s-8-3-9-387) is shared with *Gobivenator* [35], ‘*L. mcmasterae’* (UALVP 55804) [8], and *Saurornithoides* [3], but unlike other troodontids such as *Sinovenator* [37] and *Mei* [1]. The anterolateral curvature of the iliac articulation in *Troodon* appears more extreme than that of ‘*L. mcmasterae’* (UALVP 55804) [8].

*Troodon* (MOR 553s-8-3-9-387), ‘*L. mcmasterae’* (UALVP 55804) [8], and *Sinornithoides* [81] have a similar pubic shaft cross-section. The straight, nearly vertically oriented pubic shaft for *Troodon* (MOR 246–11, 430, MOR 553s-8-3-9-387) is shared with *Almas* [54] and *Gobivenator* [35], but is unlike the anteriorly curved state for ‘*L. mcmasterae’* (UALVP 55804) [8], and the opisthopubic state of *Sinovenator* [37], many dromaeosaurids [36,90,97] (with the exception of the propubic state in *Achillobator* [32]), *Unenlagia* [93], and birds [34,100,101]. The position and extent of the pubic apron in *Troodon* (MOR 553s-8-3-9-387) is similar to that of *Almas* [54], *Sinornithoides* [84], and *Talos* [56]*, Archaeopteryx* [102] as well as some dromaeosaurids such as *Shri devi* and *Velociraptor mongoliensis* [36,99]. The concavity posterior to the pubic apron for *Troodon* (MOR 553s-8-3-9-387) is similar to the medial grooves reported for *Almas* [54] and *Sinovenator* [37]. The distolateral muscle scar reported for *‘L. mcmasterae’* (UALVP 55804) [8] is not observable in any of the Two Medicine *Troodon* specimens. However, this difference could be ontogenetic.

The larger anterior projection and smaller posterior projection of the pubic boot in a large specimen (MOR 553s-8-3-9-387) is also reported for the potentially skeletally immature holotype specimen of *Almas*, IGM 100/1323 [54] and the dromaeosaurid *Achillobator* [32]. In contrast, the reversed condition of the pubic boot in the smaller specimen of *Troodon*, MOR 430, is similar to that of *Anchiornis* [82] and the presumably skeletally mature holotype specimen of *Gobivenator*, MPC-D 100/86 [35]. The Early Cretaceous troodontids *Sinovenator* [37] and *Sinornithoides* [84] lack a pubic boot with pronounced anterior or posterior projections. The pubic boot of numerous dromaeosaurids (e.g., *Adasaurus* [32]*, Microraptor* [103]), *Anchiornis* [82], unnlagiines [89,93], and many birds (e.g., *Piscivorenantiornis* [75], *Jeholornis* [104]) possess a posterior projection and lack an anterior projection entirely.

### Ischium

The sample of *Troodon* ischia includes portions of the right and left elements from both MOR 430 and MOR 748, and complete and partial right ischia from MOR 553, MOR 553l-7-27-8-87 (Fig 4) and MOR 553s-8-5-9-392, respectively. MOR 553 specimens are preserved well enough to incorporate all relevant features of the ischium. The following description and comparisons is based on MOR 553l-7-27-8-87 and MOR 553s-8-5-9-392. No differences other than size occur between these and the more poorly preserved specimens of MOR 430 and MOR 748.

Comparisons of an ischium and pubis from MOR 553 with similar corresponding suture sizes suggests that *Troodon* had a relatively large ischium, with an ischium/pubis length ratio of about 0.77, though this could be affected by the taphonomic shortening of the pubis (Table 1). In lateral view, the ischium weakly outlines a triangle with the corners marked by the proximal articulating region, point of the obturator process, and acute point of the caudodistal end.

The pubic articulation is located on an anteriorly projecting neck and is subcircular with a flattened dorsal perimeter and an irregular rugose texture. The sutural surfaces of both MOR 553 specimens match both in size and outline the ischiadic suture surface of the large right pubis from the site, MOR 553s-8-3-9-387. The iliac articulation has an elliptical outline with its long axis running anteromedial to posterolateral. A small bump and a larger raised swelling mark the two ends of this axis making the sutural surface broadly convex. The ischium’s contribution to the acetabulum forms about a 90^o^ arc between the pubic and iliac articulations. The dorsal view has a roughly hourglass outline with its narrowest portion located slightly more towards the iliac articulation. Two small foramina sit on the lateral surface just distal to the acetabulum’s edge. A narrow (~1 mm), raised edge marks the medial edge of the acetabulum. Several distinct grooves curve up from the lateral side to the acetabulum near its narrowest portion. A smooth-surfaced bump projects cranio-dorsally out of the acetabulum just below the iliac articulation.

Distal to the sutures, the ischium consists of a thickened posterior edge which arcs caudally. From this, the obturator process projects anteroventrally as a thin, triangular expanse of bone. The ventral outline of the latter arcs distinctly inwards creating a distinct notch just distal to the anteroventral tip of the obturator process whereas the outline is convex proximal to this tip. The medial surface of the ischium is very flat and bears, over its distal half, fine serrations running parallel to the long dimension of the bone. The obturator process and shaft form a slightly concave lateral surface. Muscle scars occur distally on the dorsal, lateral and lateroventral surfaces and striations and rugosities run along the medial ventral edge of the ischium. The obturator tuberosity consists of a small but distinct cranially projecting bump with irregular muscle scaring about 4 cm distal from the proximal end.

### Ischium comparisons

*Troodon* likely had a relatively large ischium. The ischium/pubis length ratio of about 0.77 for *Troodon* is greater than *Almas* (0.63) [54], *Gobivenator* (0.54) [35], *Sinornithoides* (0.53) [84], *Jianianhualong* (0.48) [86], *Sinovenator* (0.40) [37], and *Sinusonasus* (0.54) [88] (Table 1). The proximally positioned point of the obturator process in *Troodon* is shared with *Sinusonasus* [88], *Liaoningvenator* [83], *Saurornithoides* [3], *Almas* [54], and *Gobivenator* [35], as well as dromaeosaurids [36,90,97,105–107] and unenlagiines [89,93,108]. In contrast, the obturator process and caudodistal end have a subequal distal extent in *Sinornithoides* [84], *Daliansaurus* [83], *Sinovenator* [37], and *Jianianhualong* [86] as well as *Archaeopteryx* [109]. *Troodon*, *Almas* [54], and *Gobivenator* [35] all have a deep notch associated with the obturator process. This notch is not as prominent in *Saurornithoides* [3], *Dalianasaurus* [85], *Sinornithoides* [84], *Sinusonasus* [88], *Sinovenator*, and *Mei* [87]. The extent of the obturator process is greater in *Almas* [54] and even more extreme in *Liaoningvenator* [83], unenlagiines [89,93,108], and *Archaeopteryx* [109]. Unlike the convex margin of *Troodon*, the edge proximal to the tip of the obturator process is concave in unenlagiines [89,93,108] and some dromaeosaurids [36,97,105,107].The caudodistal end is an acute point in *Troodon*, *Saurornithoides* [3], *Sinusonasus* [88], and many other paravian dinosaurs [36,89,90,105,106,108,109], while this feature is more “squared off” in *Gobivenator* [35] and broadly rounded in *Almas* [54] and *Liaoningvenator* [83]. The 90^o^ arc of the acetabular contribution in *Troodon* is also present in *Gobivenator* [35], *Talos* [56], and *Buitreraptor* [89,108], but unlike the straighter contribution in *Almas* [54], *Sinovenato*r [37], *Sinusonasus* [88], *Saurornithoides* [3], *Shri* [36,107], *Deinonychus* [106], and *Unenlagia* [93]. The caudally arcing, thickened caudal edge in *Troodon* is shared with *Saurornithoides* [3], *Gobivenator* [35], and*Talos* [56]. The thickened caudal edge in these troodontids contrasts with, the more laterally-placed ridge present in many dromaeosaurids [36,97,106,107] and unenlagiines [89,93,108]. As in *Talos* [56], *Troodon* lacks the posterior processes that are present in *Sinovenator* [37], *Jianianhualong* [86],*Mei* [87], microraptorines [105,110,111]*, Rahonavis* [92], unenlagiines [89,93], and *Archaeopteryx* [102]. *Troodon*, *Talos* [56], and *Saurornithoides* [3] all have a concave lateral surface and flat medial surface of the ischium. This contrasts with the flat or nearly flat lateral surfaces of *Almas* [54], *Mei* [1], and *Sinovenator* [37]. The location of the striated and rugose medioventral ridge in *Troodon* is the ischial symphysis, which is fused in *Saurornithoides* [3].

### Femur

**Fig 5:**
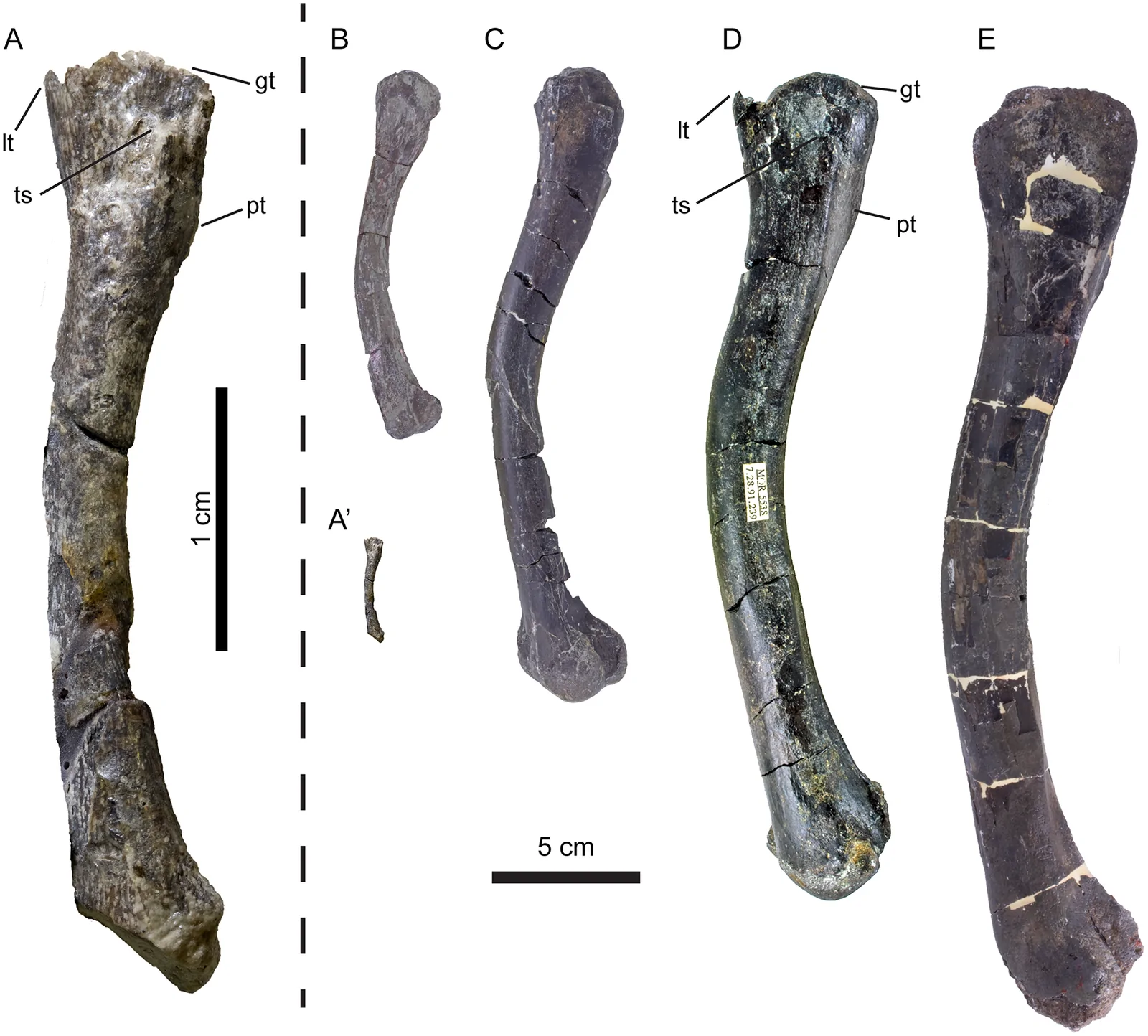
*Troodon* femora through ontogeny. *Troodon* femora in lateral view. Embryonic MOR 246–11 (A, A’); small juvenile MOR 430, reversed, (B); MOR 553s-7-28-91-234, reversed, (C); MOR 553s-7-28-91-239 (D); MOR 748, reversed, (E). Abbreviations: gt, greater trochanter; lt, lesser trochanter; pt, posterior trochanter; ts, trochanteric shelf. Scale bar = 1 cm (A) and 5 cm (A’, B-E). Note that the proximal processes of B, C, and D are taphonomically missing.

**Fig 6:**
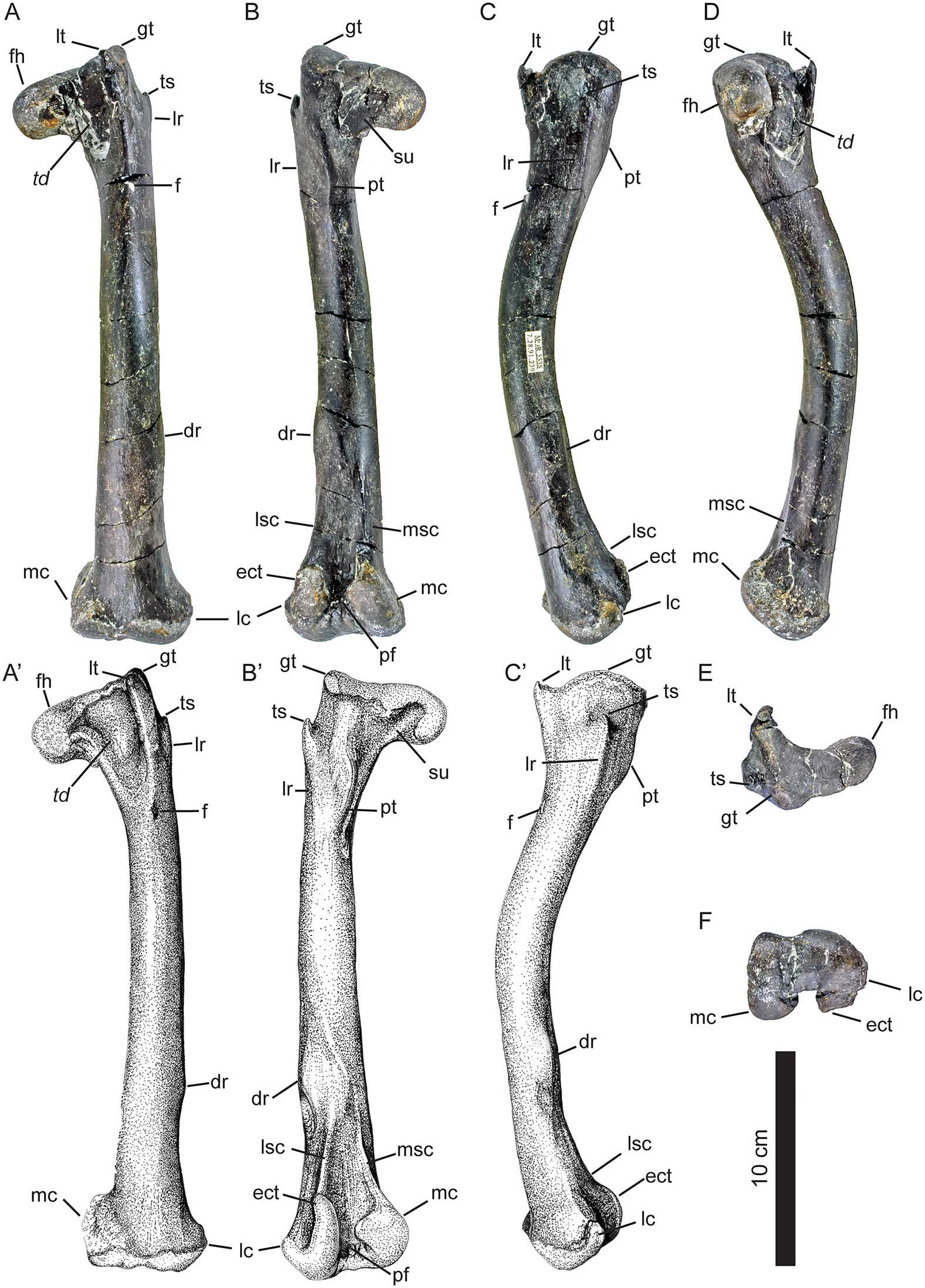
Left femur of *Troodon formosus.* Left femur (MOR 553s-7-38-91-239) in anterior (A), posterior (B), lateral (C), medial (D), proximal (E), and distal (F) views, with corresponding line drawings (A’-C’). Abbreviations: ect, ectocondylar tuber; dr, distolateral ridge; f, foramen; fh, femoral head; gt, greater trochanter; lc, lateral condyle; lr, lateral ridge; lsc, lateral supracondylar crest; lt, lesser trochanter; mc, medial condyle; msc, medial supracondylar crest; pf, popliteal fossa; pt, posterior trochanter;; *td*, taphonomic deformation; ts, trochanteric shelf.

Femur specimens include an embryonic left and right from MOR 246−11, a complete right from the small juvenile MOR 430, portions of both from the large juvenile MOR 563, a complete right and partial left from the adult MOR 748, and five isolated but complete femora from the MOR 553s, including one right (7-28-91-234) and four lefts (8-7-9-417, 7-22-91-164, 7-28-91-239, and 7-16-0-61). The sample of femora includes embryonic through adult specimens and displays a 10-fold increase in linear dimensions; greatest lengths range from 32.7 mm to over 330 mm in an adult (Fig 5). The following description and comparisons of the femur are primarily based on MOR 553s-7-2-91-239 (Fig 6) unless otherwise noted. Noted features are found on all other *Troodon* femora from MOR 553 and MOR 748. Some differences occur on the two smallest femora, the embryonic one (MOR 246−11) and the small juvenile (MOR 430).

In proximal view, the femoral head is set at a right angle to the trochanteric crest, the dorsally convex ridge formed by the greater and lesser trochanters on the proximolateral edge. Relative to the distal end of the femur, the trochanteric crest sits directly between the two distal condyles. A small notch separates the greater and lesser trochanters, which can also be observed in MOR 246 (Fig 5). The lesser trochanter is roughly level with the femoral head while the greater trochanter sits further dorsally. A small foramen perforates the anterior and slightly lateral aspect of the femur just distal to the prominence of the lesser trochanter. The circular to subcircular femoral head sits on a distinct neck. A prominent sulcus marks the posteromedial margin of the femoral head. In at least the larger specimens (e.g., MOR 553s-7-28-91-239, 7-16-0-61), several small foramina mark the floor of the sulcus. On all specimens, a small dorsally projecting knob (the trochanteric shelf) sits distal to the greater trochanter on the lateral face of the femur. A muscle scar is present anterolaterally and slightly distal to the trochanteric shelf. The posterior trochanter (sensu [3]) exists as a pronounced ridge on the posterior aspect of the femur just distal to the level of the trochanteric shelf. The long axis of the posterior trochanter is roughly parallel to that of the femoral shaft and bears some muscle scar pitting proximolaterally. Medial to the posterior trochanter, a muscle scar also runs from the base of the femoral neck to several centimeters distal to the trochanter.

The main portion of the shaft has a nearly circular cross-section and is significantly bowed anteriorly. This curvature occurs consistently throughout ontogeny from embryo to adult. Towards the distal end, the shaft broadens medio-laterally. A small ridge begins approximately two-thirds down the femur on the posterolateral border of the shaft. This distolateral ridge flattens and broadens after 2–3 centimeters in the adult specimens into a slightly raised muscle scar at its distal end. Some muscle scarring continues from this line distally down the shaft. Another ridge occurs at the same level of the shaft on the posterior aspect of the femur. This one begins laterally and angles toward the midline distally. The presence of these two ridges creates a flat facet facing posteriorly and slightly laterally.

The distal articular surface remains relatively smooth through ontogeny to subadult stage including MOR 553s-7-28-91-239 (which is 88% the length of MOR 748). However, smooth-surfaced ridges and grooves irregularly mark the surface of the largest specimens, MOR 553s-7-16-0-61 and MOR 748. The majority of the distal articular surface angles at about 50 degrees from the long axis of the femur. Anteriorly, a shallow groove separates the medial and lateral condyles. Both condyles continue onto the anterior surface of the femur briefly but have long, robust posterior extensions. Two ridges, the lateral and medial supracondylar crests run down the posterior and distal portion of the femur to support these extensions. The lateral supracondylar crest (also called the crista tibiofibularis [94]) terminates with the ectocondylar tuber distally and is separated from the lateral condyle by a distinct notch. The popliteal fossa is bounded by these ridges. While the lateral condyle tapers to a rounded point, the medial condyle is much broader. Both condyles have lateral projections.

**Fig 7:**
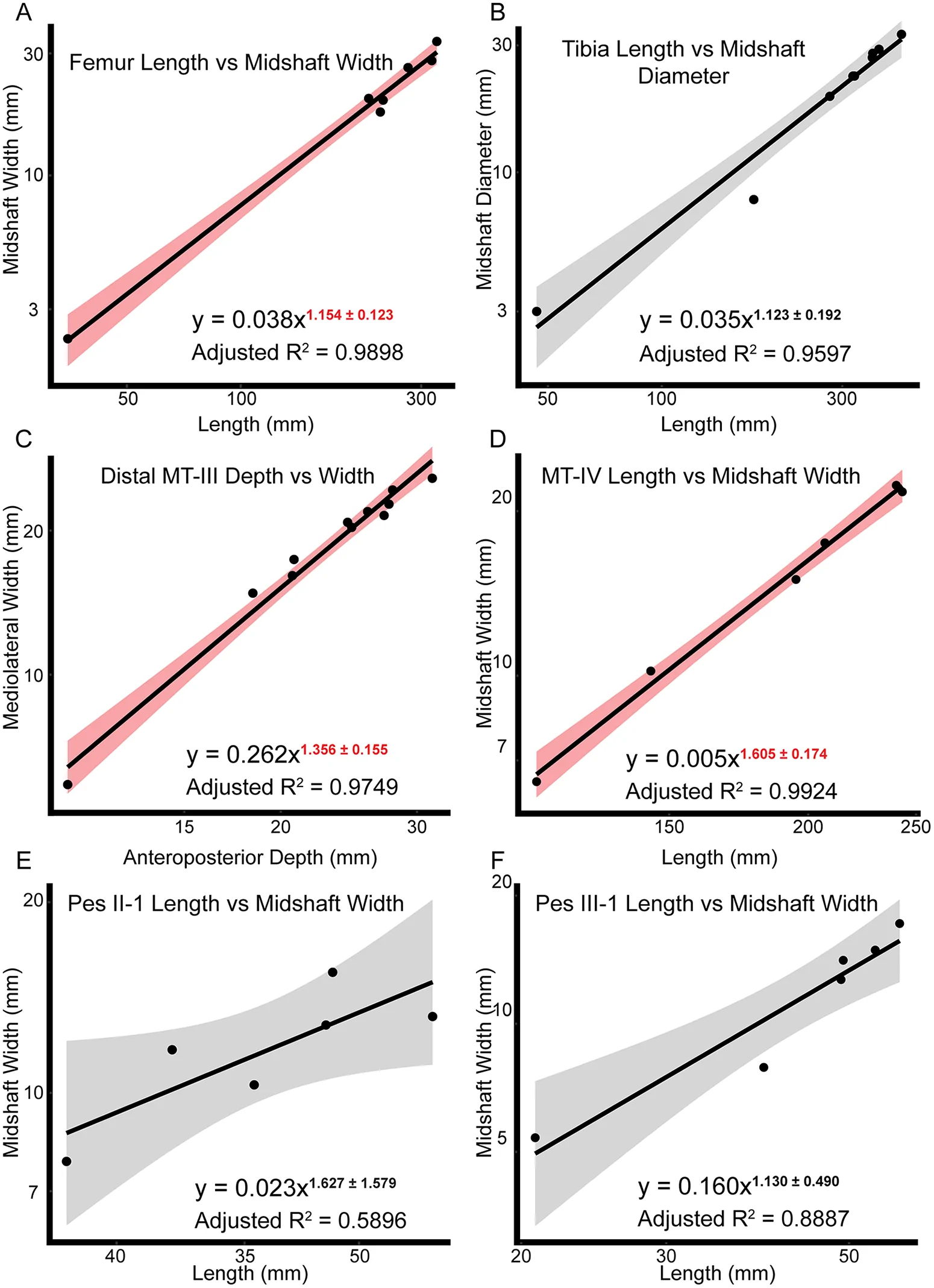
Ontogenetic trends the hindlimb of *Troodon formosus.* Regressions quantifying changes in relative dimensions for the femur (A), tibia (B), distal articulation of metatarsal III (C), metatarsal IV (D), and pes phalanx III-1 (E). Shaded regions correspond to 95% confidence interval for the regressions.

Numerous ontogenetic changes can be observed in the femur for *Troodon* (Fig 5). Initially, stoutness of this element slightly increases through ontogeny for these specimens, though it is not significantly different from isometry (Fig 7). Additionally, the surficial bone texture changes from porous in juveniles (e.g., MOR 430) to denser and smoother in adults (e.g., MOR 748). Muscle scars also become more visible through growth, with all the muscle scars being visible on femora greater than 218 millimeters in length (e.g., MOR 553s-7-28-91-234). The lateral and medial supracondylar crests develop after the ontogenetic stage of MOR 430 (femur length of 126 millimeters), but before the ontogenetic stage of individuals with a femur length of 218 millimeters (e.g., MOR 553s-7-28-91-234).

### Femur comparisons

The overall degree of curvature in the femur of *Troodon* is comparable to that of other paravian theropods [35,36,85]. The distinct neck of the femoral head in *Troodon* is shared with *Saurornithoides* [3] and *Philovenator* [51] and other paravian dinosaurs [92,93,98]. The dorsally convex trochanteric crest with a notch separating the greater and lesser trochanters in *Troodon* is also present in *Saurornithoides* [3], *Gobivenator* [35], dromaeosaurids such as *Mahakala* and *Microraptor zhaoianus* [98,110], and *Unenlagia* [93], in contrast to *Rahonavis* and some early birds [92,100,112] where the notch is absent. The greater trochanter has a greater dorsal extent than the lesser trochanter in *Troodon*, *Gobivenator* [35], *Saurornithoides* [3], *Talos* [56], and *Philovenator* [52], but opposite of *Daliansaurus* [85]. The greater dorsal projection of the trochanteric crest relative to the femoral head for *Troodon*, *Philovenator* [51], some dromaeosaurids such as *Deinonychus* and *Velociraptor mongoliensis* [90,113], and *Unenlagia* [93] is reversed in *Saurornithoides* [3]. The presence of a trochanteric shelf and associated lateral ridge distal to the greater trochanter in *Troodon* is shared with most other paravians [3,35,36,51,56,85,97,100,113,114]. As in *Almas* [54], *Talos* [56]*, Linhevenator* [79], *Philovenator* [51], and *Saurornithoides* [3] a posterior trochanter is present in *Troodon*. Unlike *Troodon*, *Talos* [56], *Saurornithoides* [3], *Sinornithoides* [81], and most dromaeosaurids [99], a small fourth trochanter is present distal to the posterior trochanter in *Linhevenator* [79], *Liaoningvenator* [83] and *Harenadraco* [78] as well as some other paravian taxa [92,97,98,114]. The small distolateral ridge as in *Troodon* may be present in *Dalianasaurus* [85], *Philovenator* [51], and *Linhevenator* [79]. *Troodon*, *Byronosaurus* [115], *Gobivenator* [35], and *Hypnovenator* [116] all possess a notch separating the supracondylar crest from the lateral condyle, unlike *Almas* [54] and *Daliansaurus* [85]. Troodon lacks the process projecting from the mediodistal end present in *Philovenator* [51]. In *Troodon* and *Gobivenator*, the lateral distal condyle is narrower than the medial condyle, unlike *Almas* [54], *Dalianasaurus* [85], *Sinovenator* [37] where the lateral condyle is wider. The large lateral projections on the distal condyles for *Troodon* are also present in *Hypnovenator* [116], but unlike dromaeosaurids [32,107,113], where these features are reduced or absent.

### Tibia

The sample of *Troodon* tibiae consists of 18 specimens ranging from embryonic material to skeletally mature. Nine of these elements (4 right and 5 left tibiae) were found disarticulated in a bonebed (MOR 553) and represent subadult to adult-sized individuals. MOR 748 preserves a complete right tibia and incomplete left. MOR 563 preserves a proximal left tibia. MOR 246–11 preserves both tibiae within the egg. MOR 246-1 preserves a partial right tibia that is largely free of the matrix. Unless otherwise stated, the description and comparisons of the tibia is based on the MOR 553s-8-16-92-254 and MOR 748

**Fig 8:**
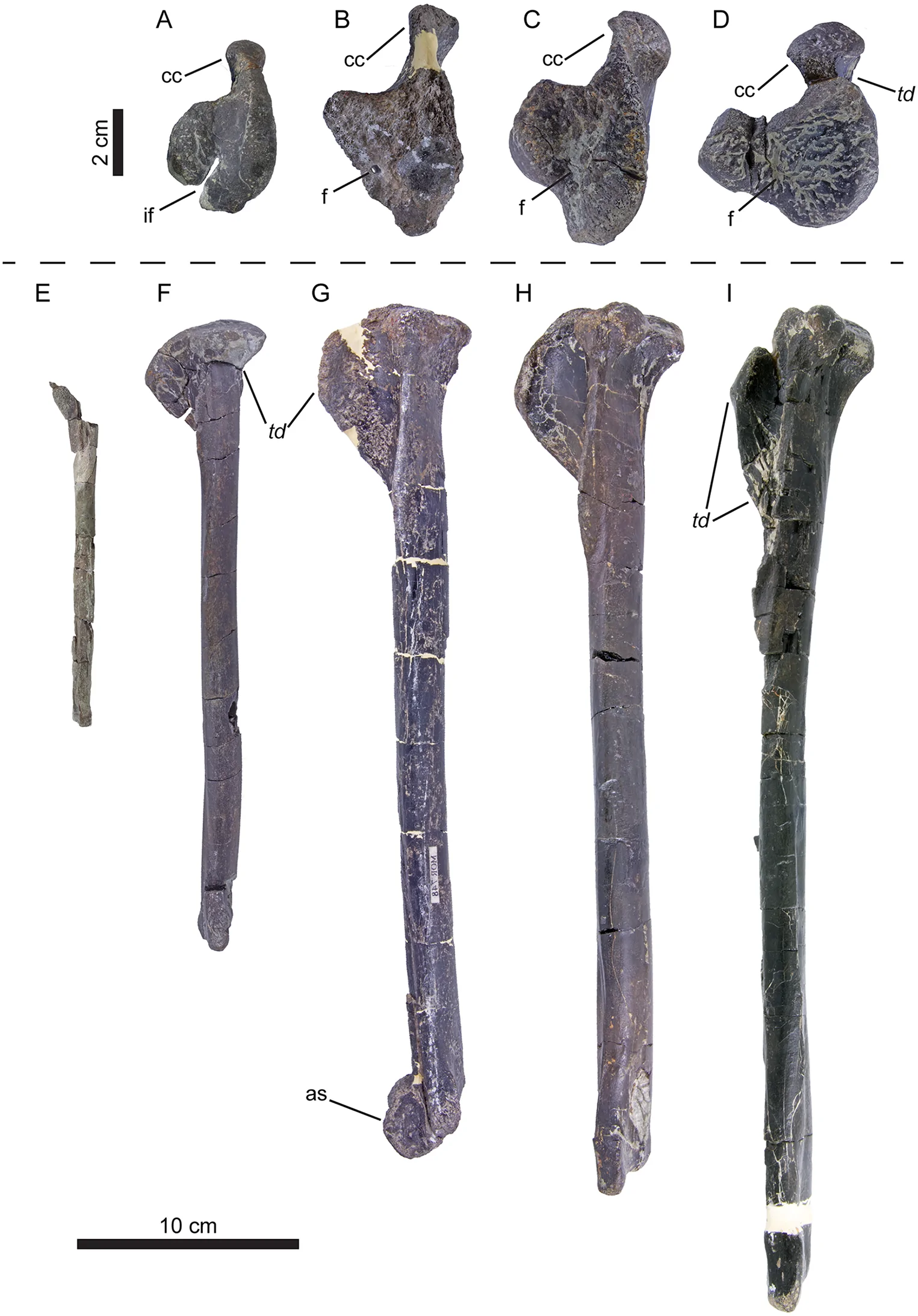
*Troodon* tibiae through ontogeny. *Troodon* tibiae in proximal (A, B, C, D) and left lateral (E, F, G, H, I) views. Small juvenile MOR 430 (E, reversed), large juvenile MOR 553s-7-20-91-132 (A, F), adult MOR 748 (B, G, reversed), adult MOR 553s-8-16-92-254 (C, H, reversed), adult MOR 553l-7-24-8-64 (D, I, reversed). Abbreviations: as, astragalus; cc, cnemial crest; if, incomplete foramen; f, foramen; *td*, taphonomic deformation.

**Fig 9:**
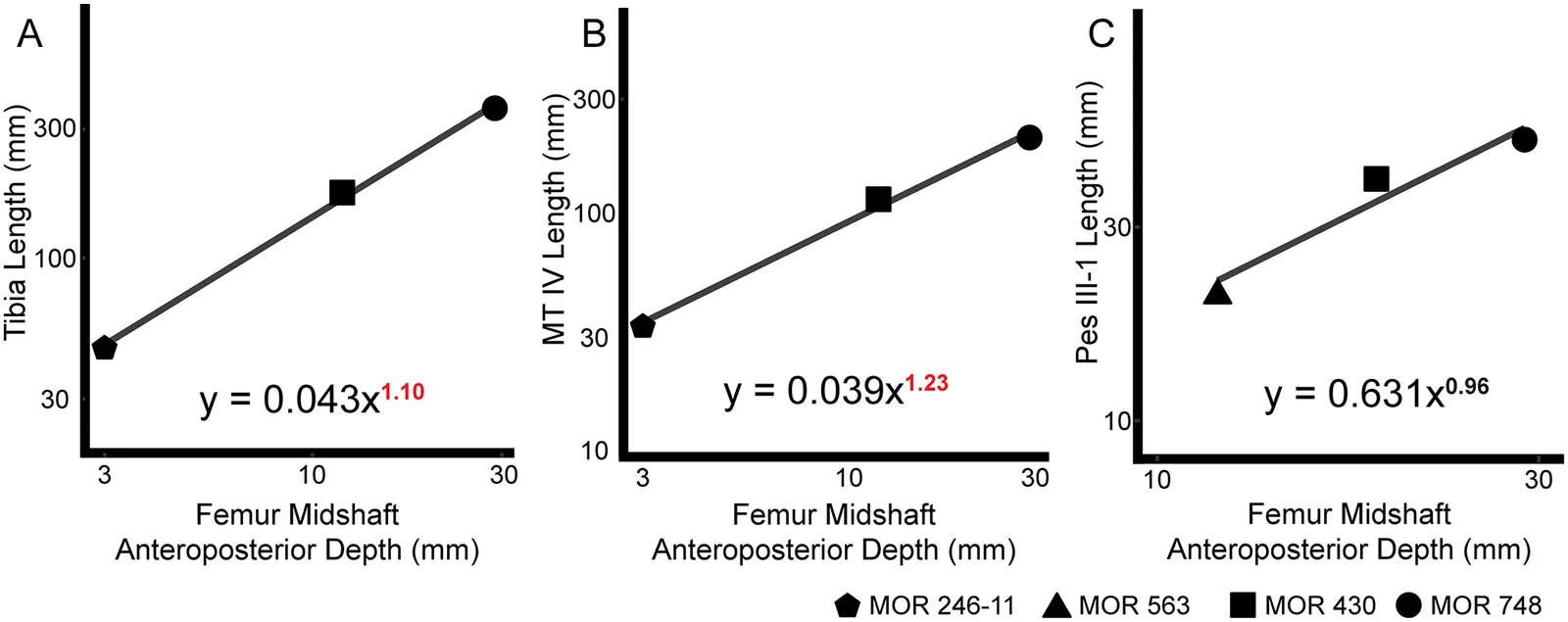
Inter-element scaling for *Troodon formosus.* Regressions of inter-element scaling for associated *Troodon* specimens for anteroposterior midshaft depth of the femur against tibia length (A), metatarsal IV length (B), and pedal phalanx III-1 length (C). Scaling coefficients in red correspond to positive allometric relationships.

**Fig 10:**
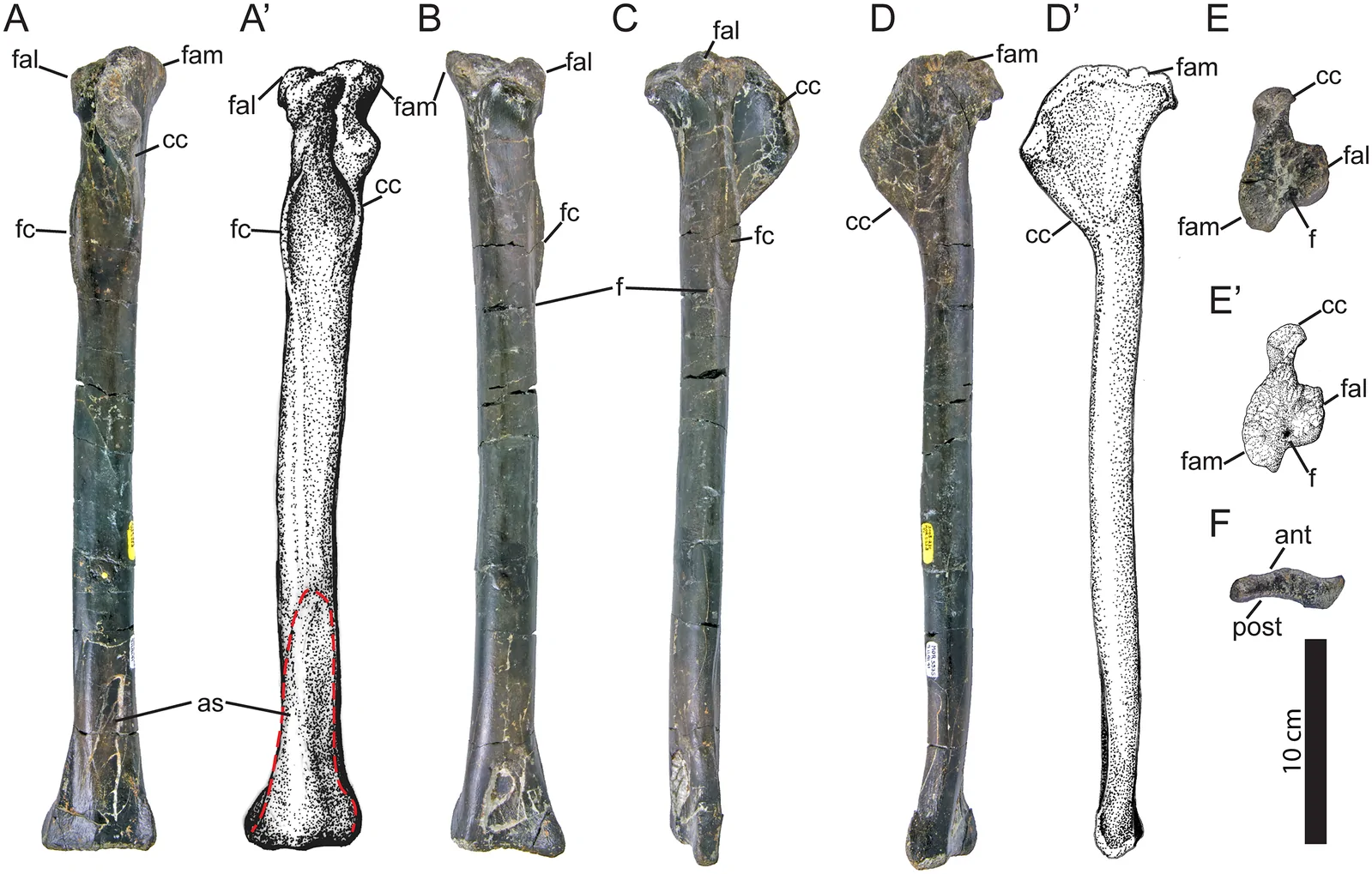
Right tibia of *Troodon formosus.* Right tibia (MOR 553s-7-11-91-41) in anterior (A), posterior (B), lateral (C), medial (D), proximal (E), and distal (F), with corresponding line drawings (A’, D’, E’). Abbreviations: ant, anterior side; as, astragalar scar; cc, cnemial crest; f, foramen; fal, facies articularis lateralis; fam, facies articularis medialis; fc, fibular crest; post, posterior side; *td*, taphonomic deformation. Red dotted line outlines the margin of the astragalar scar.

With the exception of surface texture and the undeveloped facies articularis lateralis and medialis in the embryonic tibiae (MOR 246−1, MOR 246−11), the general morphology of the tibiae is unchanged through ontogeny. Through growth, tibia robusticity (minimum midshaft diameter/length) is largely isometric (Fig 7 and 8). Although specimens at later ontogenetic stages are slightly more robust with a slightly broader distal end than smaller specimens. For associated specimens, tibia length slightly increases relative to midshaft diameter of the femur (Fig 9). The femur/tibia length ratios range from 0.72 in the small juvenile, MOR 430, to 0.89 for the MOR 748 adult (Table 1). The following description is based upon the articulated specimen MOR 748 and elements from MOR 553 (Fig 10).

The proximal end consists of a small facies articularis lateralis and a larger facies articularis medialis, the latter running continuously with the cnemial crest. These articular faces become well developed by the growth stage of the small juvenile MOR 430 (femur length of 126 millimeters). Anteriorly, the lateral and medial facies articularis are not clearly separated. In younger individuals (tibia length < 279 mm; e.g., MOR 553s-7-20-91-132), a narrow split separates the facies posteriorly. In contrast, larger specimens (lengths > 320 mm, e.g., MOR 553s-7-17-0-74) possess a bridge of bone that fuses the two (Fig 8). This bony bridge creates a foramen running through the proximal articular surface and exiting on the posterior surface of the tibia just distal to the medial and lateral facies articularis. This foramen persists in even the largest individuals (e.g., MOR 553l-7-24-8-64) and typically measures 2.0–3.0 mm in diameter. Similar to the distal end of the femur, the proximal articular surface of the tibia transitions from smooth in smaller specimens (lengths < 361 mm; e.g., MOR 553s-7-20-91-132) to being irregularly covered in ridges and grooves at larger sizes (e.g., MOR 553s-8-16-92-254). When viewed proximally, the lateral facies articularis has a square to oval outline, with relatively straight anterior and lateral margins. The lateral margin abuts the fibula. From anterior to posterior, the articular surface lies flat, then bulges proximally, and finally curves to form a proximoposterior face. The facies articularis medialis has an elongated oval outline in proximal view. It extends farther both anteriorly and posteriorly in comparison to its smaller lateral counterpart. The generally flat, articular surface slopes laterally, so that the medial margin of the facies extends most proximally. Anteriorly, the medial articular facies narrows then curves gently distally to form the cnemial crest.

The cnemial crest reaches its greatest anterior extension (the apex) roughly halfway down its proximodistal length. In larger specimens this puts the maximum extension several centimeters distal to the proximal end of the tibia. The cnemial crest remains very narrow everywhere except at its greatest anterior extension, where it swells to form a subcircular articular boss. The gentle curve away from the tibia’s proximal end and the distal location of the articular boss give the cnemial crest a “droopy” appearance when viewed medially. The fibular crest begins on the lateral margin of the tibia at a level just distal to the greatest extension of the cnemial crest. This well-formed ridge projects anterolaterally. Pronounced soft tissue attachment scarring marks the fibular crest and its base, particularly on its posterior side. A small foramen lies just posterior to the fibular crest near its distal limit.

Through the diaphysis of the tibia, the cross-section has a very slightly convex to flat anterior aspect but a fairly evenly curved posterior aspect. Soft tissue scarring marks the antero-lateral margin of much of the midshaft region. Overall, the cross-section of the tibia changes distally, widening mediolaterally and flattening anteroposteriorly.

The astragalar scar extends over the final 30% of shaft anteriorly. A low, broad ridge runs the length of this scar to the distal end of the bone. The scar is nearly symmetrical, sitting just slightly to the medial side. In MOR 430, the shape of the ascending process of the astragalus matches that of the scar. The distal articulating surface for the reception of the body of the astragalus is subrectangular and nearly flat. Two small ridges mark the distal end of the tibia. One projects posterolaterally and has muscle/ligament scars laterally. The other projects anteromedially with scarring just medial to it. The former may represent the tuberculum retinaculi m. fibularis.

### Tibia comparisons

The juvenile femur/tibia length ratio 0f 0.72 for *Troodon* (MOR 430) is similar to those of small bodied troodontids including *Mei* (~0.76) [1,87], *Liaoningvenator* (~0.67) [83], *Daliansaurus* (0.69) [85], *Sinornithoides* (~0.72) [84], and *Almas* (~0.73) [54] (Table 1). A tibia that is longer than the femur is also shared with most dromaeosaurids with the exception of *Achillobator* [36,99]. The relative widening of the shaft distally for *Troodon* is also reported for other troodontids such as *Almas* [54], *Borogovia* [117], and *Mei* [1]. The rounded cnemial crest with an apex at midheight in *Troodon* is shared with *Almas* [54], and *Harenadraco* [78], but unlike *Byronosaurus* [115], *Hypnovenator* [116], *Buitreraptor* [114], and some dromaeosaurids such as *Shri devi* and *Deinonychus* [36,106], where the apex is more proximally located. The cnemial crest extends further anteriorly from the midshaft in *Troodon* than it does in *Rahonavis* [92]. The boss on the cnemial crest in *Troodon* is present in *Velociraptor mongoliensis* [90] and *Rahonavis* [36], but absent in *Philovenator* [51]. *Troodon*, *Sinornithoides* [81], *Almas* [54], *Philovenator* [51], and *Byronosaurus* [115] lack the lateral cnemial crest that is present in *Sinovenator* [37] and *Dalianasaurus* [85]. In *Troodon, Sinovenator* [37], and *Sinornithoides* [81], the fibular crest begins on the lateral margin of the tibia at a level just distal to the greatest extension of the medial cnemial crest. *Troodon* lacks a ridge distal to the medial cnemial crest that is present in *Almas* [54] and *Sinovenator* [37]. The extent of scaring for the ascending process of the astragalus on the tibia is likely comparable (30% tibia length) to *Philovenator* [51], but greater than *Liaoningvenator* (16%) [83]. Assuming the ascending process matches the outline of the astragalar scar, the symmetry of the scar in *Troodon* is also observed in *Philovenator* [51], *Zanabazar* [3], and *Borogovia* [118].

### Fibula

**Fig 11:**
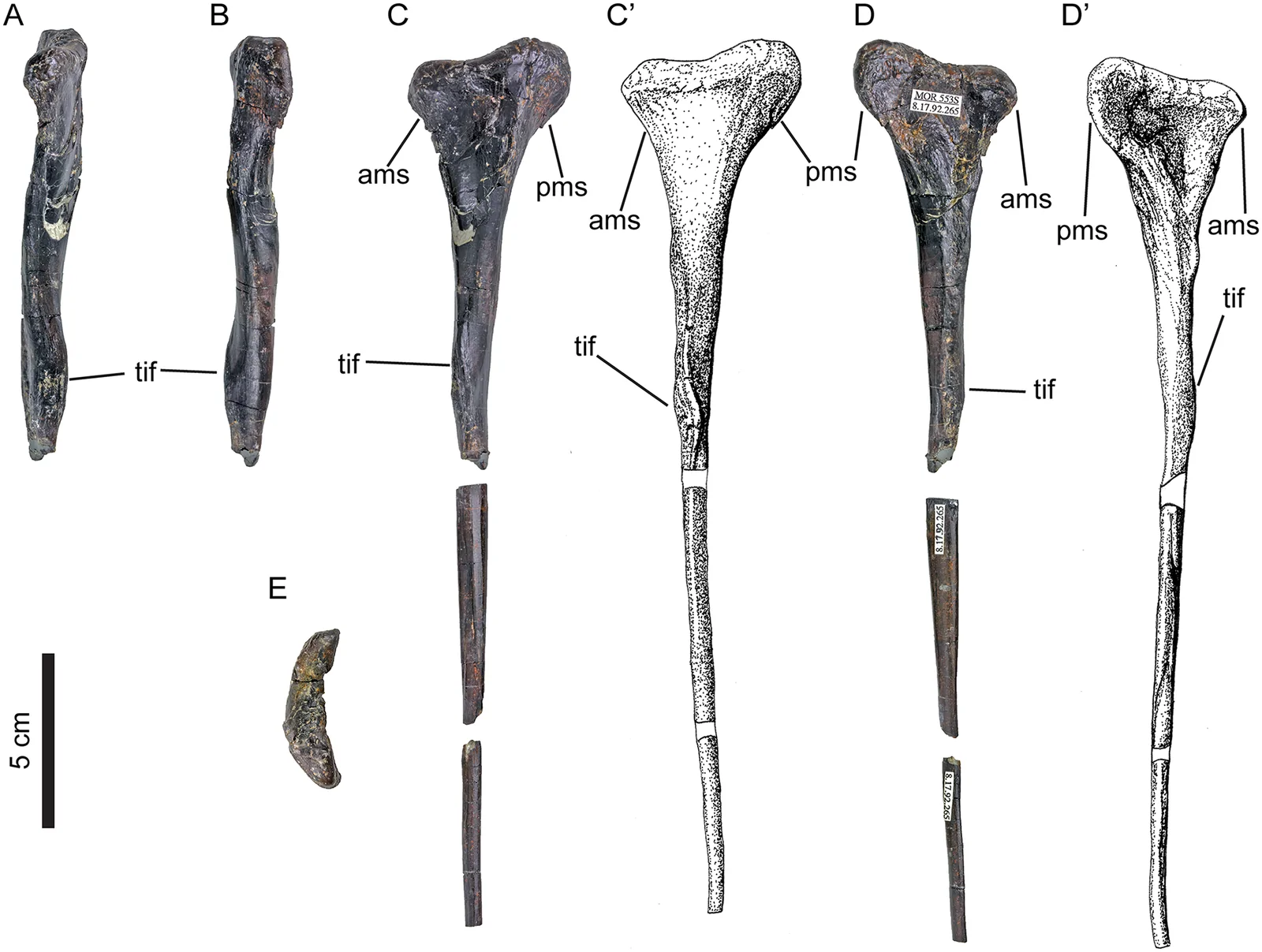
Fibula of *Troodon.* Left fibula (MOR 553s-8-17-92-265) of *Troodon* in anterior (A), posterior (B), lateral (C), medial (D), and proximal (E) views, with corresponding line drawings (C’, D’). Abbreviations: ams, anterior muscle scar; pms, posterior muscle scar; tif, tuburculum m. iliofibularis.

Fibula specimens include three elements from associated skeletons and two disarticulated elements from MOR 553. A small fragment of embryonic right fibula comes from MOR 246–11. This consists of a short (10 mm), roughly cylindrical section adhering to the fibular crest of the tibia. This exhibits no significant features and has cross-sectional diameters of 2.0 mm (anteroposteriorly) and 1.2 mm (mediolaterally). MOR 430 contains two portions of the right fibula including most of the proximal end. The final associated specimen, MOR 748, consists of the right proximal half. This has been damaged by soil corrosion and recent weathering. The two best specimens come from Jack’s Birthday Site (MOR 553) and preserve roughly the proximal two-thirds of the element. The larger, MOR 553s-8-17-92-265 (Fig 11), likely would fit a tibia of length greater than 360 mm. Unless otherwise stated, the description and comparisons of the fibula is based on MOR 553s-8-17-92-265.

The fibula begins with an expanded proximal end, but then rapidly tapers to a very narrow shaft for the last two thirds of its length. The proximal end has a concave medial surface and a convex lateral one. This gives the articulating surface a mediolaterally narrow, sub-crescentic outline in proximal view. The posterior portion of the articulating surface extends farther proximally than the anterior portion. In lateral view, the proximal end also extends farther posteriorly than anteriorly from the long axis of the fibula. Two regions of soft-tissue attachment scarring mark the lateral surface of the fibula just distal to the proximal end. An anterior one faces anterolaterally, and a posterior one faces posteriorly to posterolaterally. Further distally is the brief anterior expansion of the tuberculum m. iliofibularis. Small-scale rugosities mark this overall flat surface. The preserved remainder of the shaft has a sub-circular cross-section with a flatter medial aspect, presumably where it abutted the tibia.

The final third of the fibula is not preserved in any post-embryonic *Troodon* specimen. In MOR 246−11, the right leg appears to have the distal fibula preserved. It looks, as suggested, to run the length of the tibia and lie on that anterolateral shelf. In post-embryonic specimens, its shape of the distal end can be inferred based upon the structure of the tibia, astragalus and calcaneum. None of the known calcanea bear a facet for articulation with the fibula. Nevertheless, as previously described [58] and as shown by three more Two Medicine specimens, the tibia and astragalus form a narrow groove for the fibula. This lies between the lateral edge of the ascending process of the astragalus and the anterolateral edge of the tibia. This groove extends to the level of the calcaneum. Consequently, the fibula in *Troodon* apparently extended the length of the tibia but lacked an articulation for the calcaneum. The fibula is unfused to the tibial shaft in *Troodon*.

### Fibula comparisons

The expanded proximal end and rapid distal tapering of the fibula that lacks an articulation with the calcaneum for *Troodon* is also present in other troodontids [35,51,54,83–85,114,119,120], *Rahonavis* [92], and many birds [119]. By contrast, some dromaeosaurids such as *Deinonychus* [32,106] and *Archaeopteryx* [102] have a more robust fibula that articulates with the calcaneum. The greater proximal extent of the posterior portion of the articular surface for *Troodon* is reversed in *Liaoningvenator* [83]. The greater posterior extent of the proximal end in *Troodon* is shared with *Liaoningvenator* [83], *Almas* [54], and *Gobivenator* [35]. The fibula is possibly fused to the tibial shaft in *Sinornithoides* [84], unlike the *Troodon* material described here.

### Calcaneum

**Fig 12:**
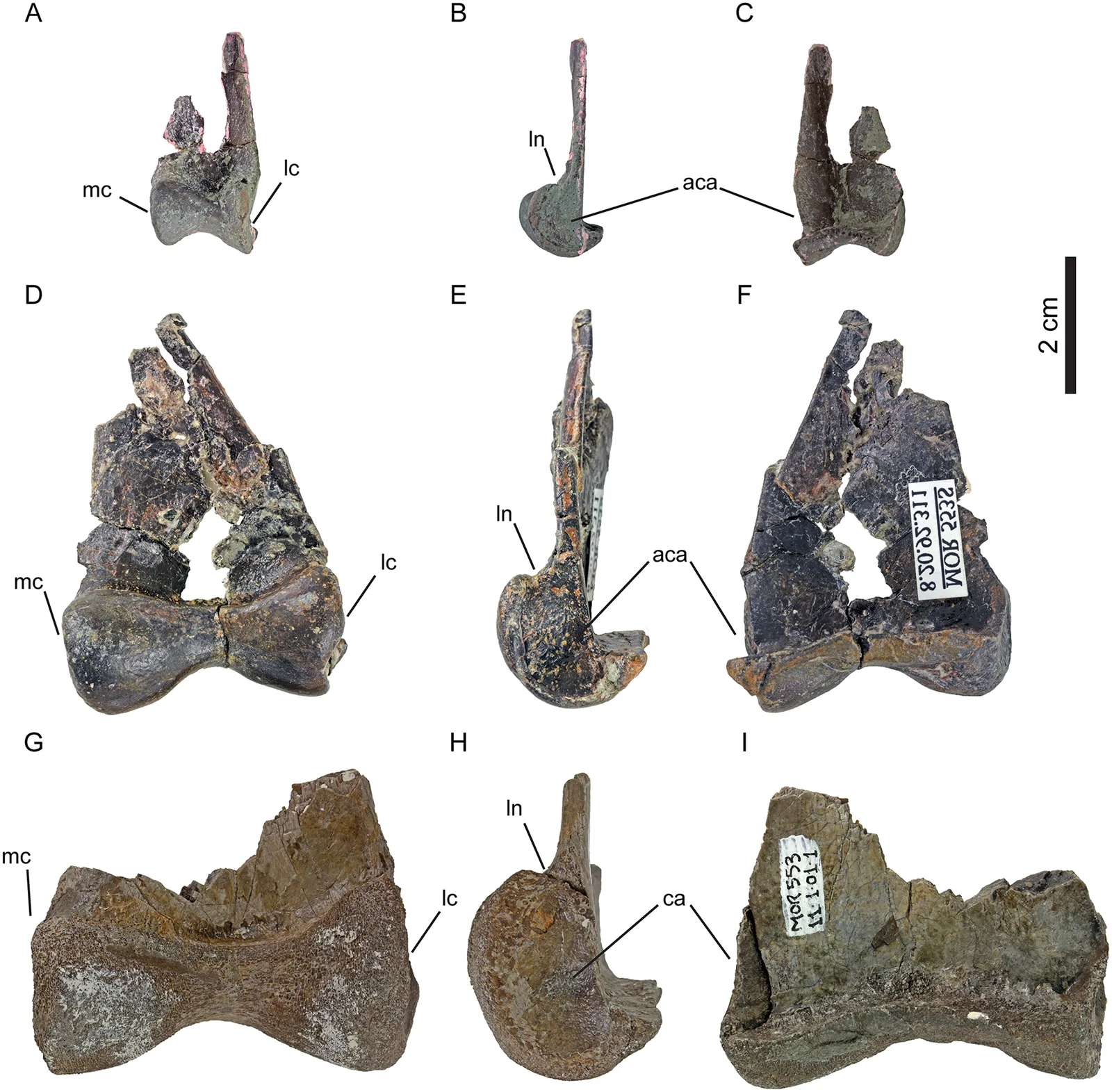
Astragalus and calcaneum of *Troodon* through ontogeny. Right astragalus of MOR 430 (small juvenile, reversed) in anterior (A), lateral (B), and posterior (C) views. Right astragalus from MOR 553s-8-20-92-311 (large juvenile, reversed) in anterior (D), lateral (E), and posterior (F) views. Left astragalus and calcaneum from MOR 553-11-1-01-1 (adult) in anterior (G), lateral (H), and posterior (I) views. Abbreviations: aca, absent calcaneum; ca, calcaneum; lc, lateral condyle; ln, lateral notch; mc, medial condyle.

No example of an isolated calcaneum exists for *Troodon*. The three calcanea specimens remain tightly appressed or are fused to the astragalus. In MOR 430, the small juvenile, the left calcaneum lies tightly against the astragalus, but the right calcaneum is absent from its astragalus. There is thus no indication of fusion at this early ontogenetic stage. In the two adult specimens (MOR 553s-11-1-01-1, 748), the calcaneum appears at least partially fused to the astragalus (Fig 12). Fusion is most extensive anterodistally and least so posteriorly, where a clear gap exists between astragalus and calcaneum. In all specimens, the calcaneum is a very thin element transversely, fitting snuggly or fusing onto the lateral depression of the astragalus. The calcaneum largely sits anterior to the distal tibia when in articulation (as in MOR 748) and has a roughly semi-lunate outline in lateral view. The element is concave laterally. The calcaneum lacks any sign of an articular facet for receiving the fibula. A small pit sits about mid-length on the lateral side of the two adult specimens. When articulated with the astragalus, the calcaneum presents a flattened posterior surface that articulates with a portion of the anterior face of the distal tibia. When MOR 553-11-1-01-1 is articulated with its associated tibia, a narrow anterior-facing surface lies lateral to the calcaneum. If this represents a contact between the distal tibia and fibula, contact with the calcaneum would have been minimal.

### Calcaneum comparisons

Few calcanea have been described for troodontids, likely due to the diminutive form of the element as well as ease of loss through taphonomic processes. The semi-lunate outline and lateral concavity of the calcaneum in *Troodon* specimens contrasts with the sub-circular, convex shape in *Liaoningvenator* [83]. In comparison to troodontids, the calcaneum is relatively wider in some dromaeosaurids such as *Shri devi* and *Deinonychus* [32,106] and the unenlagiine *Buitreraptor* [114]. The lack of an articular facet for the fibula in *Troodon* is shared with other troodontids [51,83,84,120] and birds [119], but not *Deinonychus* [121].

### Astragalus

**Fig 13:**
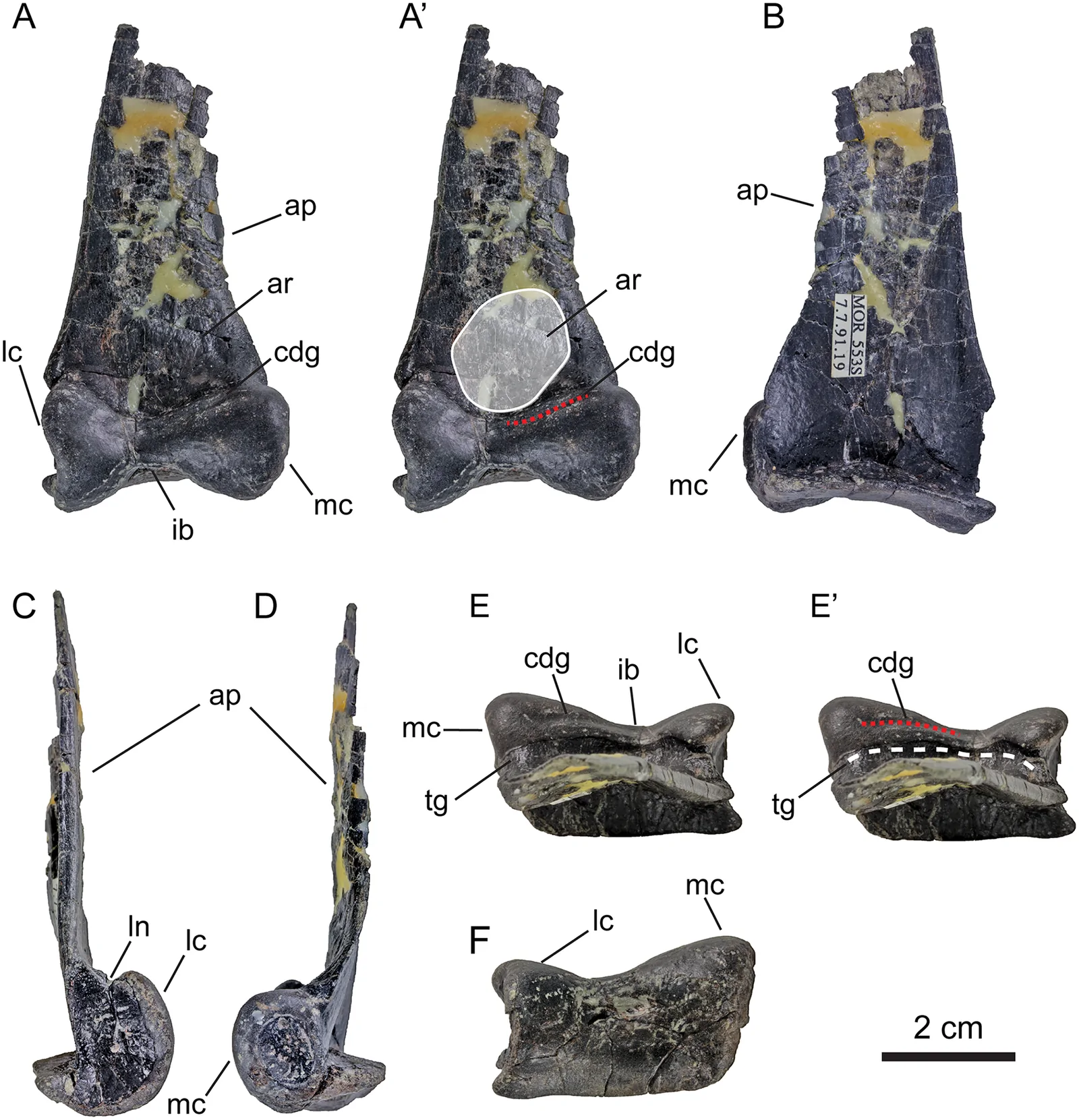
Astragalus of *Troodon formosus.* Right astragalus (MOR 553s-7-7-91-19), a large juvenile, in anterior (A, A’), posterior (B), lateral (C), medial (D), proximal (E, E’), and distal (F) views. Abbreviations: ap, ascending process; ar, anterior rugosity; cdg, craniodorsal groove; ib, intercondylar bridge; lc, lateral condyle; ln, lateral notch; mc, medial condyle; tg, transverse groove. Color/line annotation: red dashed line, craniodorsal groove; white dashed line, transverse groove; white shape, margins of anterior rugosity.

The sample of *Troodon* astragali from the Two Medicine Formation includes at least 9 specimens represented by a four-fold increase in condylar width and over a two-fold increase in overall length (Fig 12). Two of the elements are from the small juvenile specimen, MOR 430, five are from MOR 553 (Fig 13), and one is from MOR 748. Unless otherwise stated, the description and comparison of the astragalus for *Troodon* is based on MOR 553s-7-7-91-19, a large juvenile.

**Fig 14:**
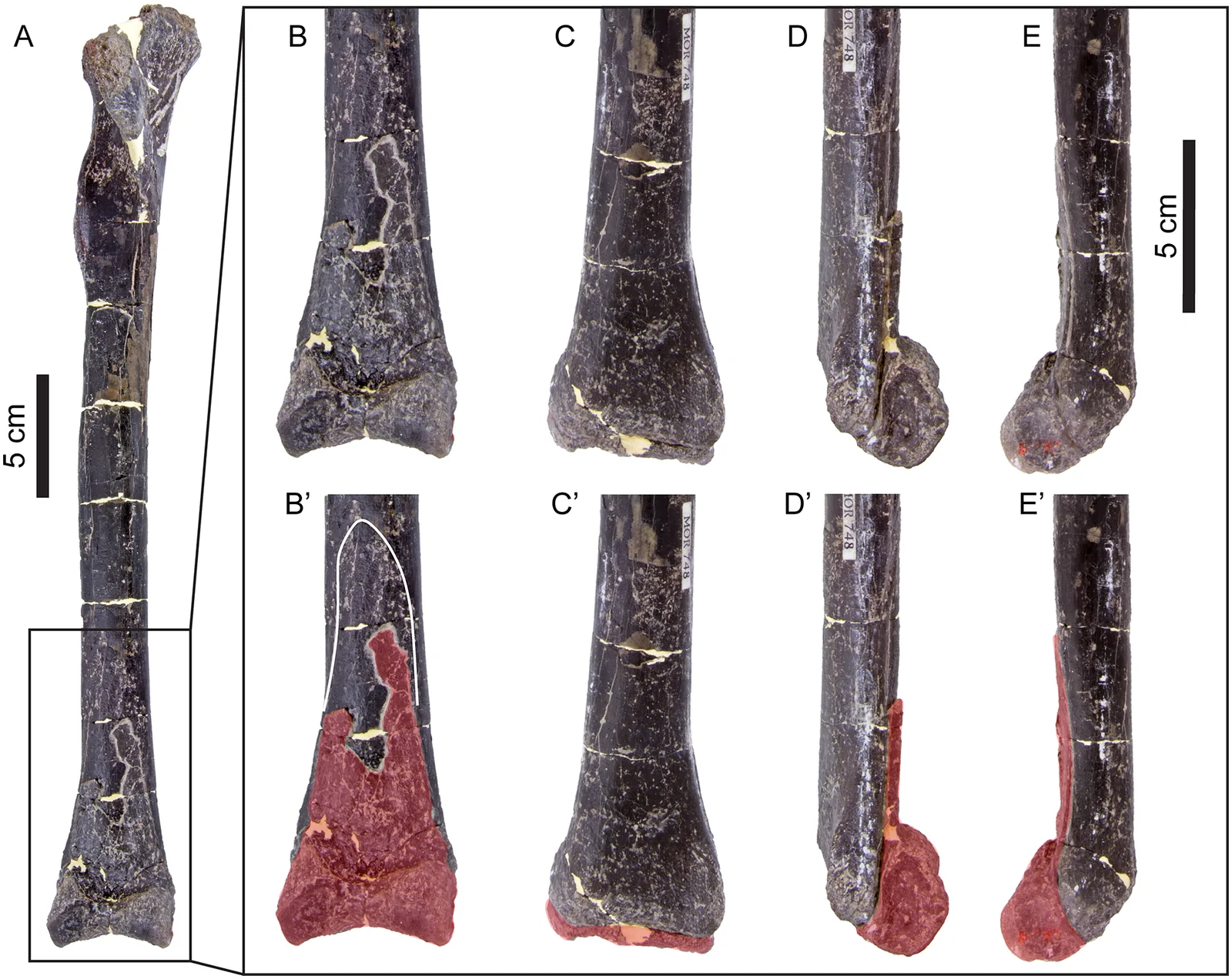
Astragalus and tibia articulation for *Troodon formosus.* Right tibiotarsus (MOR 748), an adult, in anterior (A) view, distal end of tibiotarsus in anterior (B, B’), posterior (C, C’), lateral (D, D’), and medial (E, E’) views. Color/line annotation: red coloration, preserved portions of astragalus; white line, approximate margin of astragalar scar.

When in articulation with the tibia (as in MOR 748), both condyles of the astragalus sit largely anterior to the distal end of the tibia (Fig 14). A thin shelf extends posteriorly capping the entire distal end of the tibia. This shelf bears a slight midline rise proximally which fits into a correspondingly gentle concavity in the end of the tibia. The shelf also has a slight lateral projection that abuts the distal end of the calcaneum posteriorly. With the proximal tarsals in articulation with the tibia, a narrow anterior strip of the tibia remains exposed lateral to the ascending process and calcaneum and likely accommodated the distal fibula. There is no evidence for fusion between the astragalus and tibia in any specimen.

In the smallest specimen, MOR 430, both condyles are considerably narrower relative to their length but maintain uniform proportions throughout the remaining specimens and sizes (Fig 12). For the larger specimens (e.g., MOR 553s-8-20-92-311, 553-11-1-01-1), the medial condyle is transversely broader, proximo-distally shorter, and anteroposteriorly longer than the lateral condyle. In medial view, the medial condyle has a convex profile that extends proximal to the base of the ascending process. A pronounced oval concavity sits on the medial aspect of the medial condyle. Both condyles narrow towards the intercondylar bridge. The craniodorsal aspect of the medial condyle bears a faint transverse groove that runs through the intercondylar bridge sometimes extending onto the medial portion of the lateral condyle. The lateral condyle has a sigmoidal lateral edge bordering the lateral depression for the calcaneum.

The tall ascending process is exceedingly thin proximally and is incomplete in all the specimens except MOR 430. Overall, it is convex anteriorly and concave posteriorly where it contacts the tibia. On the posterior aspect, a faint midline groove bifurcates distally with a branch arcing laterally and medially. The ascending process terminates in a thickened edge laterally and a thin edge medially. There is a distinct notch between the ascending process and the dorsal margin of the lateral condyle in *Troodon* [56]. Distally, the calcaneum fits medially to the anterolateral edge, so that the edge and lateral surface of the calcaneum form a nearly confluent lateral margin. A midline pit marks the very base of the ascending process on its anterior aspect. This pit opens proximally and consequently is largely hidden in anterior view. Depth of the pit varies among the specimens and appears independent of size. A nearly straight, transverse proximal groove is also present at the base of the ascending process. A short distance proximally from the base of the ascending process of the astragalus sits a small bump or ridge. Typically, the most proximal point of this feature sits near the midline. As it extends distally it angles medially, so that it is typically centered medial to the midline of the element.

### Astragalus comparisons

The degree to which the condylar portion of the astragalus covers the distal tibia in *Troodon* (e.g., MOR 430, 748) is similar to that of *Zanabazar* [3], *Harenadraco* [78], *Philovenator* [52], *Sinornithoides* [84], *Velociraptor mongoliensis* [90], *Deinonychus* [106], and unenlagiines [32,114], but differs from *Mei* [1] where the astragalus extends onto the posterior surface of the tibia. *Troodon*, *Gobivenator* [35], and *Talos* [56] all possess a medial condyle that is transversely broader and proximo-distally shorter than the lateral condyle, the opposite condition to that of *Sinornithoides* [81]. In *Zanabazar* [3] and *Harenadraco* [78], the medial condyle is proximodistally taller and transversely broader than the lateral condyle. The medial condyle is anteroposteriorly longer than the lateral condyle in *Troodon*, *Sinornithoides* [81], *Sinovenator* [56], *Borogovia* [118], and *Philovenator* [51], while the condyles have “subequal” anteroposterior depth in *Talos* [56]. The extent of the medial condyle proximal to the base of the ascending process in *Troodon* contrasts with the distally sloping medial condyle of *Talos* [56]. Anteriorly, the dorsal margin of the medial condyle in *Troodon* and *Sinornithoides* is convex, while this margin is more horizontal in *Talos* and *Sinovenato*r [56].

The pinching of the condyles towards the midline of the astragalus for *Troodon* is present in most paravian taxa [2,3,32,54,78,81,90,92,114,116,118], but is more extreme in *Talos* [56] than *Troodon*. The transverse groove on the dorsal aspect of the astragalar condyles in *Troodon* is also present in *Mei* [87], but absent in *Talos*, *Sinornithoides*, and *Sinovenator* [56]. The sigmoidal edge of the lateral condyle in *Troodon* is shared with *Zanabazar* [3] and *Talos* [56]. The notch on the dorsal margin of the lateral condyle in *Troodon* is not as extreme in *Talos* [56], *Harenadraco* [78], *Borogovia* [118], or *Zanabazar* [3].

The midline pit at the anterior base of the ascending process in *Troodon* is also present in *Zanabazar* [3], *Sinornithoides* [81], *Hypnovenator* [116], and *Talos* [56], but not *Philovenator* [51] and *Liaoningvenator* [83]. The nearly straight groove at the base of the ascending process in *Troodon* contrasts with the “v-shaped” groove in *Talos* [56]. The small bump near the base of the ascending process for *Troodon* is also shared with *Zanabazar* [3]. The anteroposteriorly thin medial margin of the ascending process for *Troodon* contrasts with the thicker edge in *Talos* [56].

### Distal tarsal III

A small oval disk of bone from MOR 430 may represent the only isolated tarsal III (measuring 6.7 by 5.8 by 1.3 mm). This element is nearly featureless except for being flat on one side and weakly convex on the other. The size is appropriate given the dimensions of tarsal IV in MOR 430.

### Distal tarsal IV

**Fig 15:**
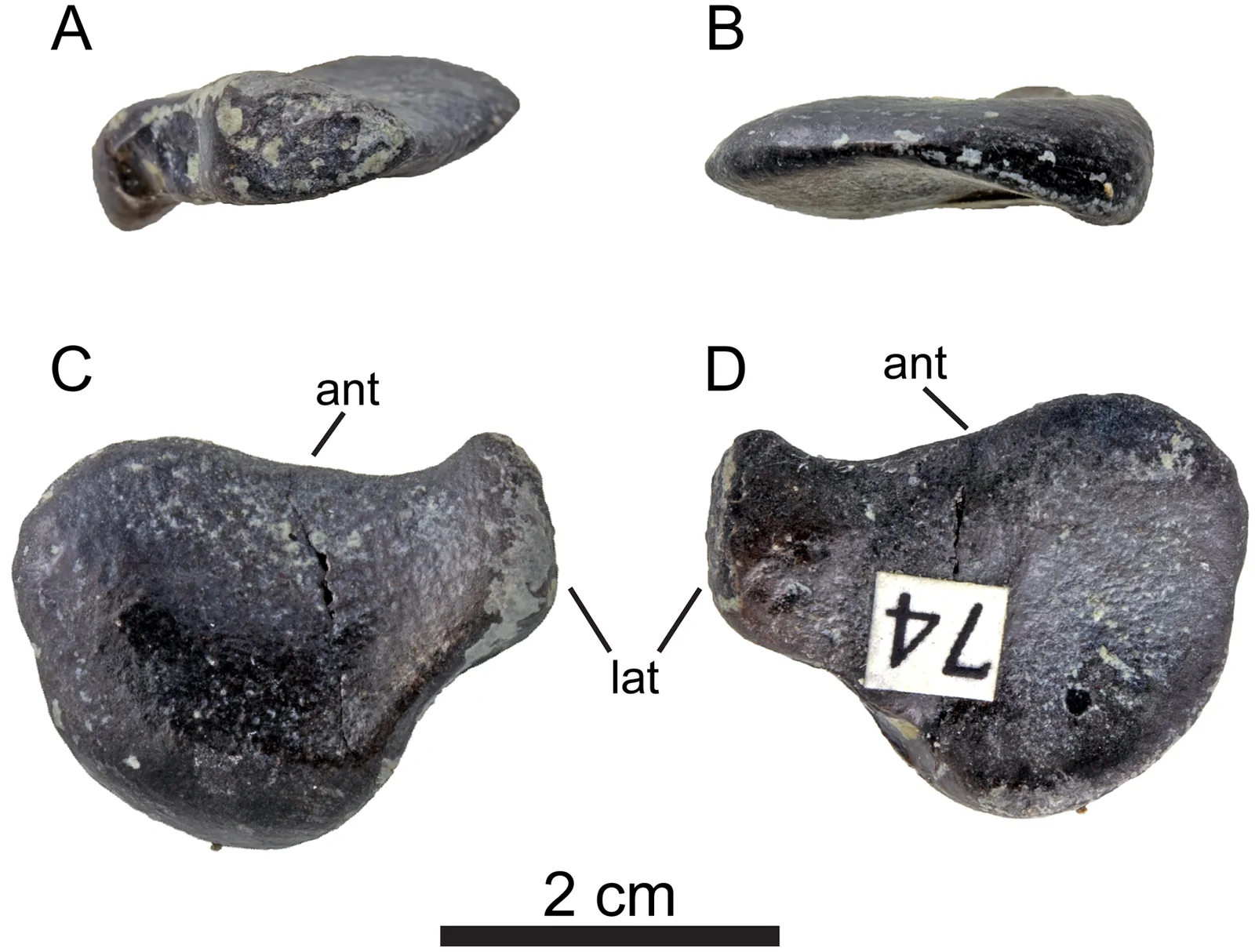
Distal tarsal IV of *Troodon formosus.* Right distal tarsal IV (MOR 553s-7-16-91-74) in lateral (A), medial (B), proximal (C), and distal (D) views. Abbreviations: ant, anterior side; lat, lateral side.

The sample of distal tarsal IV for *Troodon* includes a right from MOR 430 and two rights and a left from MOR 553 (MOR 553s-7-16-91-74, Fig 15). Distal tarsal IV (e.g., MOR 553s-7-16-91-74) is a thin wedge of bone. In general, the element has a short posterior, a short medial, and a longer anterior edge. The lateral edge angles in such a way to accommodate metatarsal V. Tarsal IV is thickest posterolaterally and thins anteriorly. Nearly the entire surface of the specimens from MOR 553 has a flat, dark-gray finish, a texture indicative of a cartilage cover. Only one small patch of black glossy bone occurs, this as a slight posterolaterally facing concavity along the thick lateral edge. Two or three foramina exit the bone here.

### Distal tarsals – MOR 748

**Fig 16:**
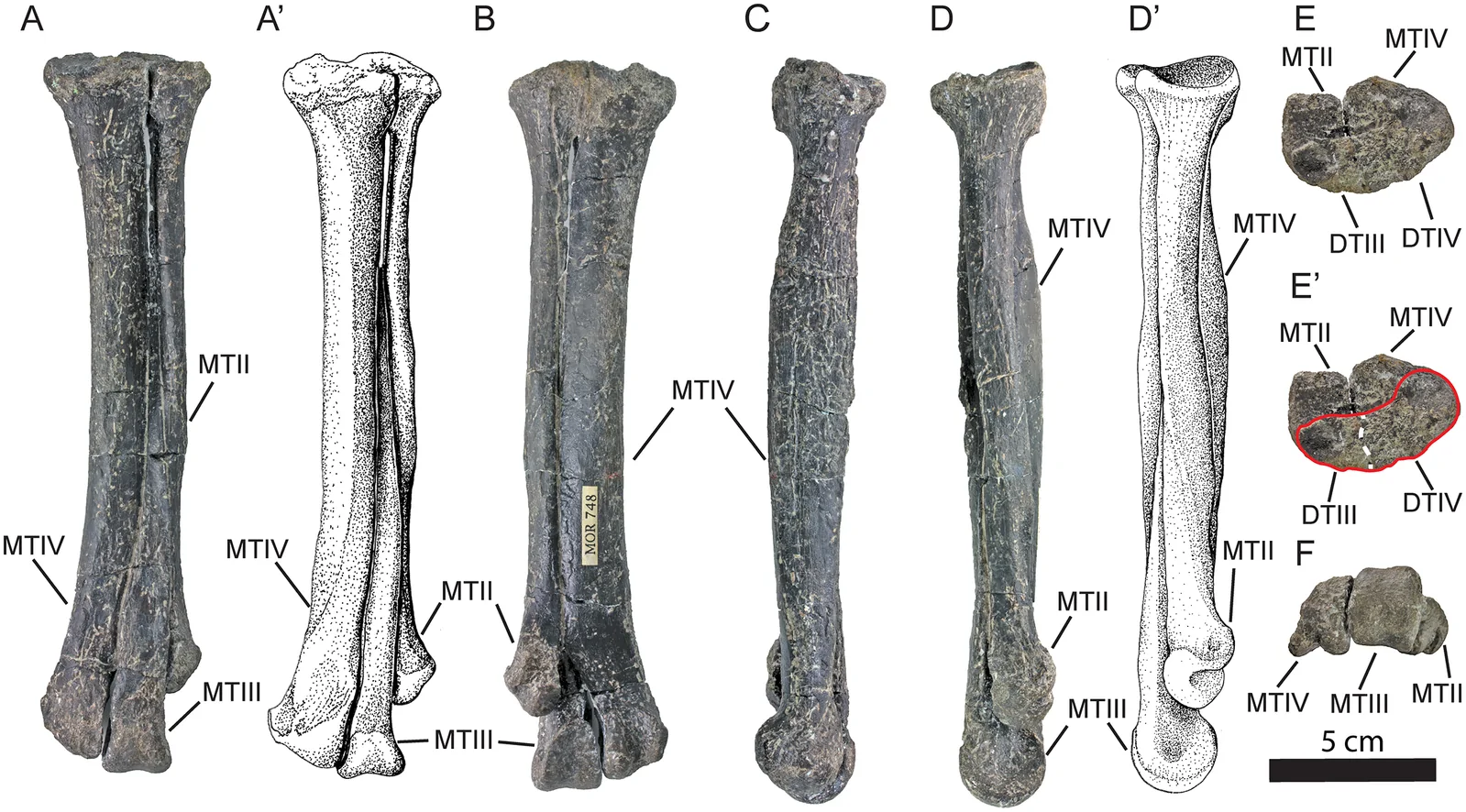
Articulated tarsometatarsus of *Troodon formosus.* Right tarsometatarsus of MOR 748 in anterior (A), posterior (B), lateral (C), medial (D), proximal (E), and distal (F) views, with corresponding line drawings (A’, D’) views. Red solid line illustrates outline of distal tarsals on the metatarsus (E’). White dotted line illustrates the approximate contact between distal tarsals III and IV. Abbreviations: DTIII, distal tarsal III; DTIV, distal tarsal IV; MTII, metatarsal II; MTIII, metatarsal III; MTIV, metatarsal IV.

The distal tarsals of MOR 748 are preserved incompletely in place and may be fused to the proximal ends of the right metatarsus, but they are unfused to each other (Fig 16). Although the boundary remains unclear, it appears that tarsals IV and III cover metatarsal IV and metatarsals II and III, respectively. While metatarsal III is completely obscured, the tarsals cap only the posterior halves of the proximal articulations for metatarsals II and IV.

### Distal tarsal comparisons

Although limited material is available for comparison, the morphology of distal tarsal IV remains very consistent across troodontids. Distal tarsal IV for *Sinornithoides* [84] shares with *Troodon* (MOR 553s-7-16-91-74) a similar angle or step for the accommodation of metatarsal V. Coverage of metatarsal IV by tarsal IV in *Troodon* (MOR 748) is shared with *Jianianhualong* [86] and *Zanabazar* [120]. It is important to note that for *Zanabazar*, however, Norell et al. [3] were unable to identify a boundary between the fused distal tarsals interpreted by Barsbold [120] and instead suggest the possibility of this element being composed of a single tarsal. The coverage of tarsal III is more variable across taxa. In both *Troodon* (MOR 748) and *Zanabazar*, tarsal III covers the entirety of metatarsal III and the posterior portion of metatarsal II [3]. In *Sinornithoides*, it covers only the posterolateral corner, of MT II [84]. Coverage of the metatarsals by the distal tarsals is similarly variable in dromaeosaurids [90,106].

### Metatarsal I

**Fig 17:**
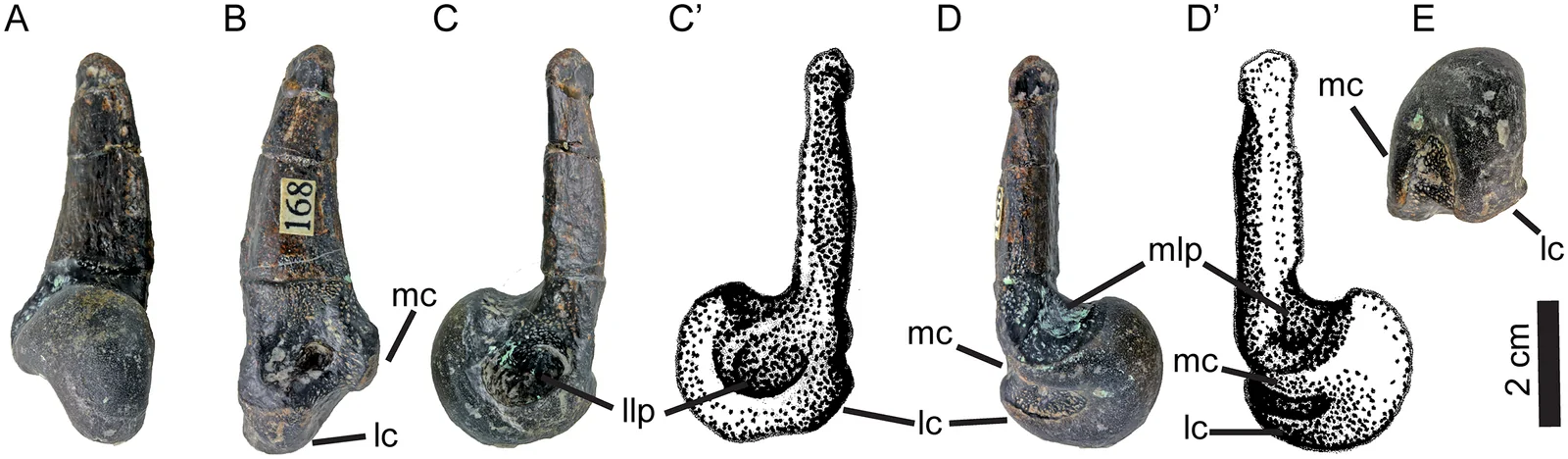
Metatarsal I of *Troodon formosus.* Left metatarsal I (MOR 553s-8-6-92-168) of *Troodon* in anterior (A), posterior (B), lateral (C), medial (D), and distal (E) views, with corresponding line drawings (C’, D’). Abbreviation: lc, lateral condyle; llp, lateral ligament pit; mc, medial condyle; mlp, medial ligament pit.

Three specimens of metatarsal I include an associated element from MOR 748 and two isolated ones from MOR 553 (MOR 553s-8-2-91-293 and 8-6-92-168) (Fig 17). With the exception of a possible pathology on MOR 748, all metatarsal I specimens are consistent in morphology. The element is narrowest proximally but transversely expanded distally. Throughout its short length, the shaft maintains a fairly constant anteroposterior thickness. The flattened lateral edge is positioned to angle both the body of the shaft and the articular condyle slightly laterally. Metatarsal I angles medially away from metatarsal II.

The relatively small shaft abruptly expands distally to a large and bulbous articular condyle. Laterally, the condyle bears a very large and deep ligament pit. The medial pit is absent or barely discernable in comparison. The articular condyle has a large lateral portion oriented proximodistally and a smaller medial portion. A small non-articular surface partially separates these two posterodistally. The articular surface sweeps through a large arc of roughly 255^o^, passing from posterior to near proximal facing. This suggests a wide range of movement for phalanx I-1. The shaft of MOR 748 bears an irregular, medioanterior projection. This creates a small medial facing concavity with the rest of the shaft that appears to be a pathology.

### MT-I comparisons

The distal expansion of metatarsal I in *Troodon* is shared with *Sinornithoides* [81], *Gobivenator* [35], *Linhevenator* [79], *Jianianhualong* [86], *Philovenator* [51], *Talos* [56], and other paravian taxa [90,92,106]. The large and deep lateral ligament pit for *Troodon* is also present in *Linhevenator* [79], but not *Dalianasaurus* [85]. The larger lateral condyle in *Troodon* is shared with *Dalianasaurus* [85], *Sinovenator* [37], and *Sinornithoides* [81]. This is opposite *Talos* [56] and *Linhevenator* [79]. A pathologic metatarsal I as in MOR 748 is also reported for the *Sinornithoides* holotype [81].

### Metatarsal II

**Fig 18:**
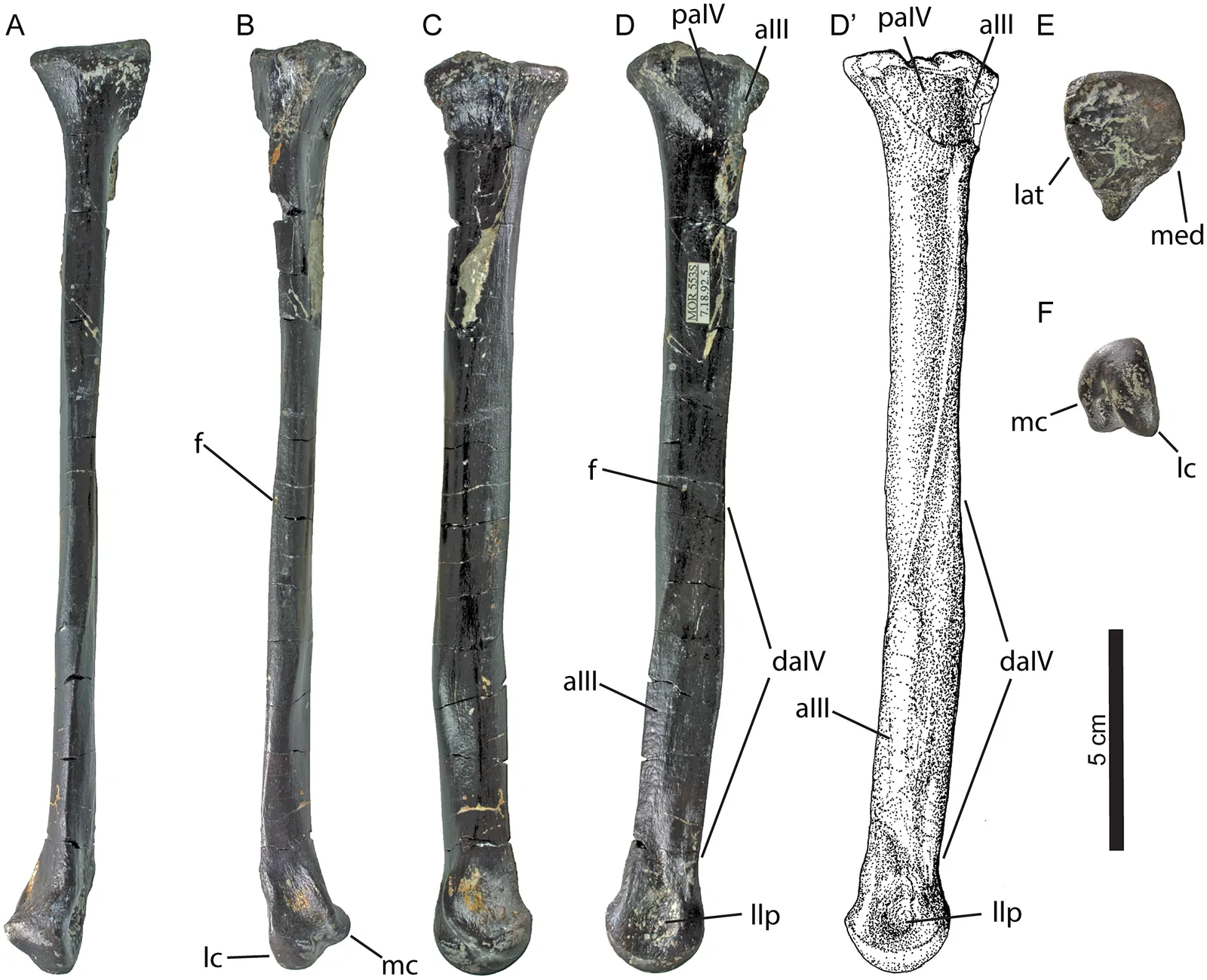
Metatarsal II of *Troodon formosus.* Left metatarsal II (MOR 553s-7-18-92-5) in anterior (A), posterior (B), lateral (C), medial (D) with corresponding line drawing (D’), proximal (E), and distal (F) views Abbreviations: aIII, articulation with metatarsal III; daIV, distal articulation with metatarsal IV; f, foramen; lat, lateral surface; lc, lateral condyle; llp, lateral ligament pit; mc, medial condyle; med, medial surface; paIV, proximal articulation for metatarsal IV.

The sample of metatarsal II for *Troodon* include ten specimens. Six of these specimens are paired from MOR 430, 563, and 748. The remaining four specimens (three left and one right) are from MOR 553. The description and comparisons of metatarsal II is largely based upon the two most complete and well-preserved specimens, MOR 553s-7-18-92-5 (Fig 18) and the right MOR 748 (Fig 16) unless otherwise stated.

**Fig 19:**
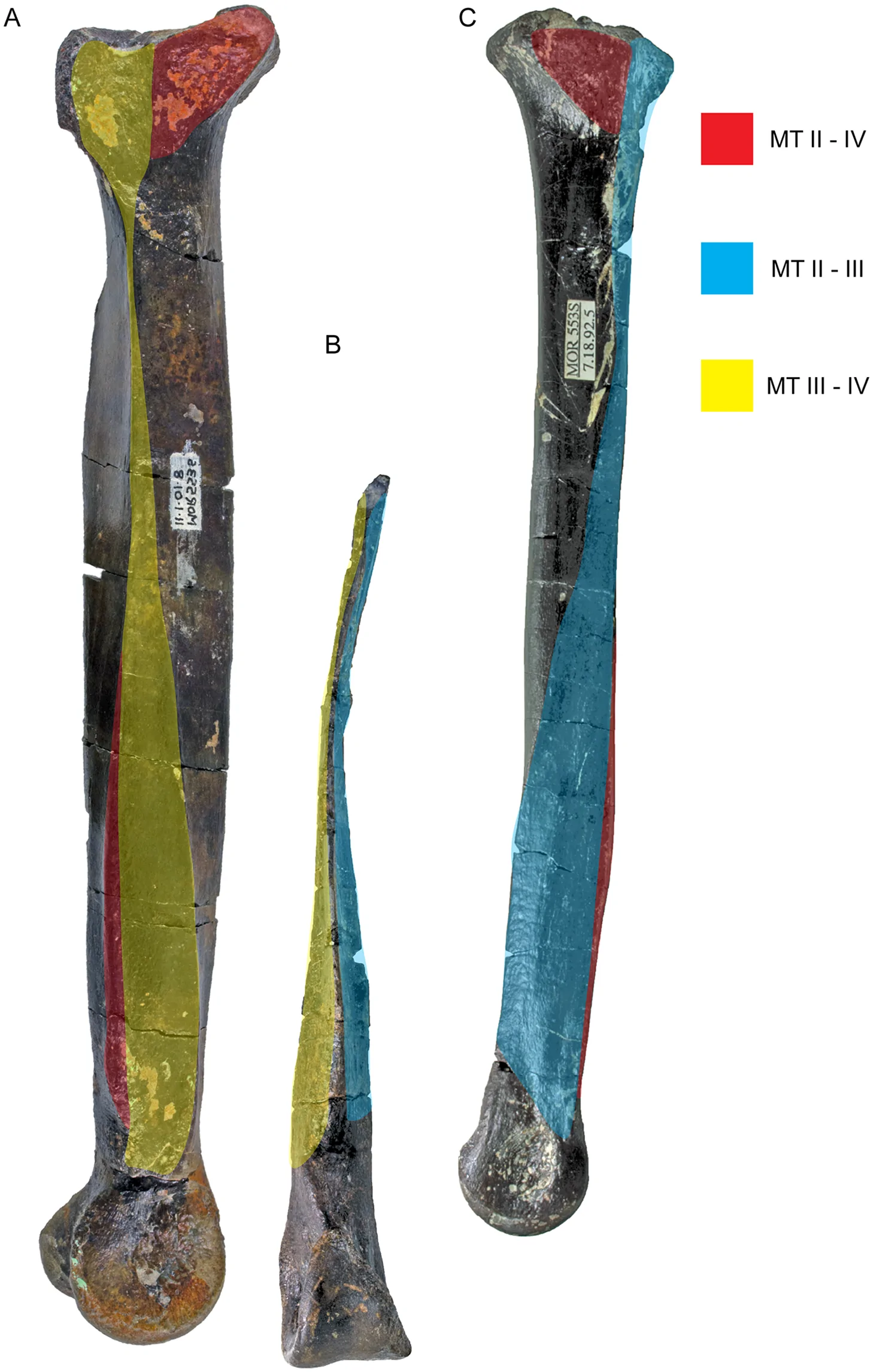
Metatarsal articulation in *Troodon formosus.* Right metatarsal IV (MOR 553s-11-1-01-8, reversed) in medial view (A), left metatarsal III (MOR 553s-8-6-9-406) in posterior view (B), left metatarsal II (MOR 553s-7-18-92-5) in lateral view (C). Abbreviations/color annotations: MT II – IV (red), articular surface between metatarsals II and IV; MT II – III (blue), articular surface between metatarsals II and III; MT III – IV (yellow), articular surface between metatarsals III and IV. Elements scaled to proportions of MOR 748.

The proximal articular surface is anteroposteriorly deeper than mediolaterally broad. These proportions are exaggerated in the smallest specimen, MOR 430. In all specimens, the proximal surface is slightly concave and has a nearly straight lateral edge. The anterior, medial, and posterior borders form a nearly continuous subcircular arc. Near its lateral termination, the posterior edge bears a small, triangular posterior projection. The articular surface of this extension drops slightly distal to the plane defining the main articular surface of the metatarsal. Distal to this posterior projection lies a small triangular and slightly concave lateral face. This surface wraps around the medial side of the proximal end of metatarsal III when the metatarsus is articulated (Fig 19). Anterior to this facet is a second and larger sub-triangular lateral surface. This extends distally from the straight lateral edge of the bone’s proximal end. This face articulates with a similar medial face on metatarsal IV.

Overall length of metatarsal II is significantly shorter than the subequal metatarsals III and IV. The elongate metatarsal II has a remarkably straight and laterally compressed shaft. At midshaft, the mediolateral width is roughly 0.55 of the anteroposterior depth. The shaft is less than half the width than metatarsal IV. Throughout the length of the shaft, the width remains less than 0.60 that of the depth through ontogeny. Medially, the shaft face is slightly convex to nearly flat. A slight thickening with some muscle scarring marks the posterior edge of the distal shaft. In MOR 553s-7-18-92-5, this thickening sits about two-thirds of the way down from the proximal end and extends over about 25 millimeters of the shaft’s length. Proximal to this rugosity on the posterior edge of the shaft lies a small foramen. There is no clear mark or scarring for the attachment of metatarsal I.

A contact for metatarsal III runs along almost the entire length of the shaft laterally (Fig 19). The articular surface shifts from being laterally oriented proximally to anterolaterally oriented distally. A narrow, flat face extends from the small proximal triangular surface described above and continues along the posterolateral edge of the shaft before expanding and angling anteriorly at approximately midshaft so that over the distal third of the shaft, the enlarged metatarsal III articulation runs along the anterolateral edge. Anterior to this the proximal articulation, the lateral and proximal half of the shaft is slightly concave. Distally, as the articulation for metatarsal III shifts from a posterior to anterior position of the lateral aspect of the shaft, it defines an attenuated triangular face with a proximally directed apex. This lateral face articulates with the distal portions of metatarsal IV. Consequently, metatarsal II has two separate contacts for metatarsal IV. The first is a triangular proximoanterior contact. The second articulation is elongate and sits distoposteriorly. The latter extends over the distal half of the metatarsal II shaft. The articular contact for metatarsal III separates the two metatarsal IV contacts.

The distal articular surface of metatarsal II is quite similar to that of metatarsal I and is not ginglymoid. The asymmetric articular condyle consists of a larger, more distally extending lateral portion oriented parasagittally and separated from a smaller, more medially oriented portion by a small non-articular surface distoposteriorly. A large collateral ligament pit marks the second metatarsal laterally. A small pit is variably present medially. The medial pit is absent in the smallest specimen, MOR 430, and largest in the biggest specimens. The articular surface of metatarsal II differs from that of the metatarsal I in that it sweeps through a smaller arc of about 180 degrees. A small, roughened patch of bone lies just proximal to the articular surface on the anterolateral edge of the shaft.

### MT II comparisons

Descriptions of metatarsal II assigned to *Stenonychosaurus* (e.g., NMC 8539) and *‘L. mcmasterae’* (e.g., NMC 12340) are largely consistent with the Two Medicine *Troodon* material [57,58,122]. The shaft being less than half the width and shorter than metatarsal IV in *Troodon* is shared with most troodontines [8,35,54,56,78,79,120,123,124]. This contrasts with many non-troodontine troodontids [2,37,81,87,88], *Philovenator* [51], and other paravians [36,92,97,102] where the shafts of metatarsals II and IV are roughly equal in width. Compared to *Troodon*, the lateral compression of metatarsal II is more extreme in *Philovenator* [51] and less extreme in dromaeosaurids [36,90,106].

The outline of the proximal end of metatarsal II in *Troodon* is shared with *Zanabazar* [3] and *Talos* [56]. In contrast, *Mei* [87], *Sinornithoides*, *Sinovenator*, *Sinusonasus*, MPC-D 100/140 [125] (an unnamed troodontid from the Lower Cretaceous of Mongolia) and some dromaeosaurids and birds [36,97] have a prominent posteromedially to posteriorly projecting ridge, which is absent in *Troodon*. A posteromedially-placed foramen on the midshaft of *Troodon* is also present in *Tamarro* [5]. The arrangement of the articulations for metatarsal II with III and IV in *Troodon* is consistent with that of other arctometatarsalian troodontids [54,56,79,123]. The presence of the distoposteriorly located MT II-IV contact in *Troodon* is not present in non-arctometatarsalian troodontids such as *Sinornithoides* [81]. The non-ginglymoid distal articulation in *Troodon* is shared with most troodontids [5,8,37,56,81,117,125,126], unlike dromaeosaurids (e.g., [32]) and *Buitreraptor* [114]. The relative sizes and orientations of the lateral and medial portions of the distal articular condyle in *Troodon* is shared with *Talos* [56], IGM 100/44 [126], ‘*L. mcmasterae’* (e.g., TMP 1992.036.0575) [8], and *Tamarro* [5]. This contrasts with *Borogovia*, where the two portions of the articular surface have a subequal proximal extent [117]. The relatively large lateral ligament pit in *Troodon* is also present in MPC-D 100/140 [125], IGM 100/44 [126], and *Tamarro* [5]. The variably present, smaller medial pit in *Troodon* is present in MPC-D 100/140 [125] and *Tamarro* [5] while it is absent entirely in *Liaoningvenator* [83], IGM 100/44 [126], and *Linhevenator* [79]. Unlike *Troodon*, the lateral ligament pit in *Borogovia* continues proximally as a groove [118].

### Metatarsal III

**Fig 20:**
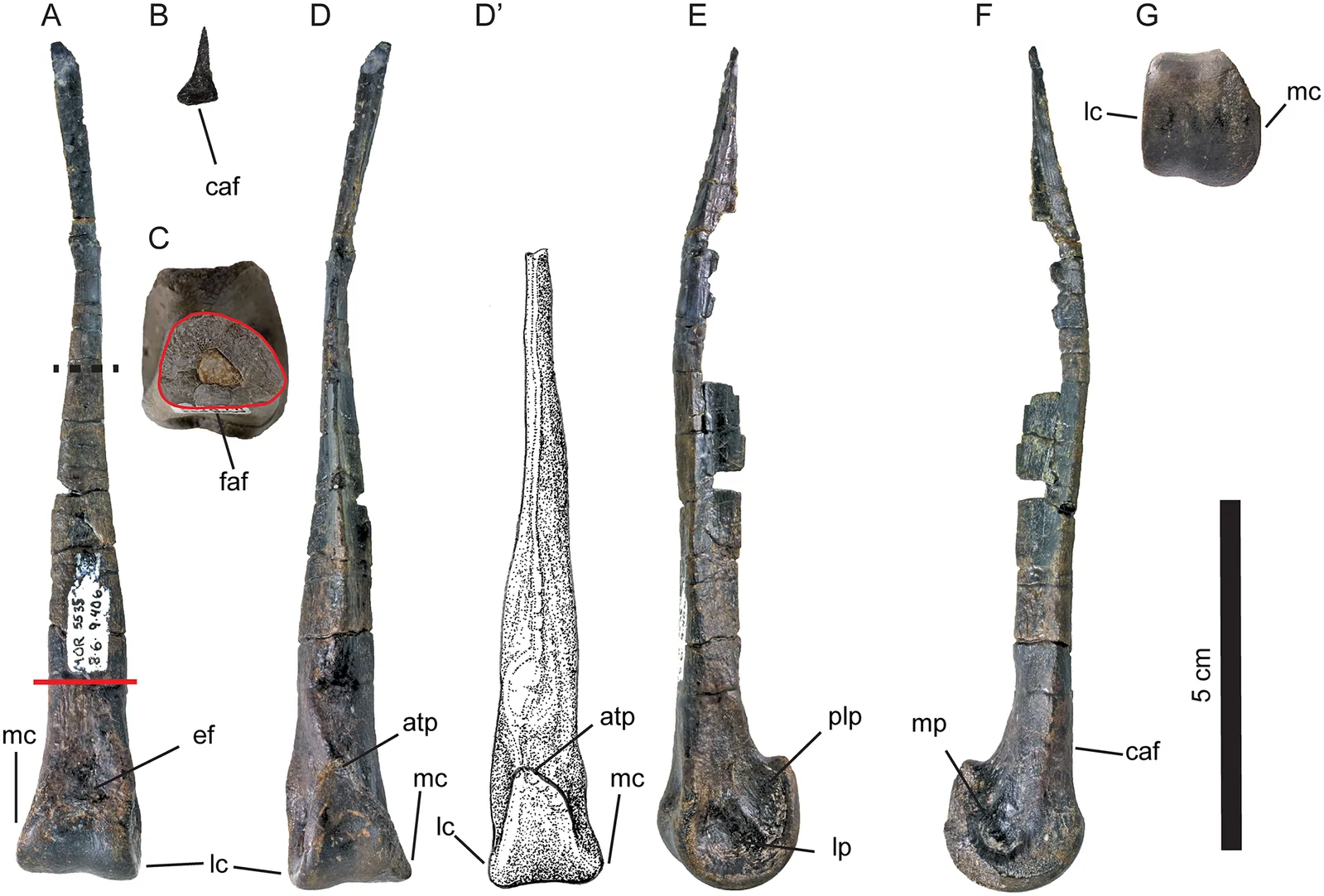
Metatarsal III of *Troodon formosus.* Left metatarsal III (MOR 553s-8-6-9-406) of *Troodon* in anterior (A), posterior (D) with corresponding line drawing (D’), lateral (E), medial (F), and distal (G) views, which shows a convex anterior face. Proximal cross section of left metatarsal III (MOR 748), showing a convex anterior face in proximal view (B); distal cross section of right metatarsal III (MOR 553-11-01-7) (C), which shows a flat anterior face. Abbreviations: atp, asymmetrical tongue-like projection of the distal articular surface; caf, convex anterior face; ef, extensor fossa; faf, flat anterior face; lc, lateral condyle; lp, lateral pit; mc, medial condyle; mp, medial pit; plp, proximal lateral pit. The black dotted line (A) corresponds to the approximate location of the cross section of MOR 748 (B). The red line (A) corresponds to the approximate location of the cross-section of MOR 553-11-1-01-7 (C) outlined in red.

The sample for metatarsal III in *Troodon* from the Two Medicine includes fourteen specimens. Six of these specimens are paired from MOR 430, 563, and 748. MOR 493 consists of a left metatarsal. The remaining seven specimens (four lefts, three rights) are from MOR 553. Most of the available specimens consist only of the distal articular end or a portion of the shaft near this. Three MOR 553 specimens also include a significant portion of the shaft (Fig 20). Only the right metatarsal III from MOR 748 is preserved in its entirety (Fig 16). The description and comparisons of metatarsal III is based MOR 553s-8-6-9-406 and MOR 748 unless otherwise stated.

Metatarsal III is arctometatarsalian. The relative length of metatarsal III (the longest element of the metatarsus) for *Troodon* is short relative to the femur with a MT III/femur length ratio of 0.66, as represented by MOR 748 (Table 1). At its narrowest, the shaft diameter is only 1% that of the greatest length. The metatarsal begins with a wedge-shaped proximal end that fits between the posterior portions of metatarsal II and IV. Metatarsals II and IV meet anteriorly and obscure the proximal third of metatarsal III from the extensor surface. The posterior position of metatarsal III and the deep shafts of the adjacent metatarsals create a deep longitudinal sulcus on the extensor surface of the proximal metatarsus. Over the first 40% of its length, the shaft is at its thinnest both mediolaterally and anteroposteriorly, contributing to the arctometatarsalian condition. In MOR 748, metatarsal III is 212 mm long, but over this proximal portion, the shaft is only 2–3 mm wide.

Beginning at about one-third down its length, the metatarsal angles to the anterior surface of the metatarsus. This corresponds to a change in the shaft cross-section from strap-like to triangular with a slightly convex anterior edge and an attenuated apex pointing posteriorly. Because of these changes, over much of the final third of its length, metatarsal III sits wedged between and slightly anterior to metatarsals II and IV. The shaft also exhibits a slight lateral bend distally, so that the distal articular surface sits slightly lateral to the proximal. This curvature is readily visible in flexor view of isolated metatarsals, where the generally straight posterior ridge arcs laterally just prior to the distal articular surface. This distal curvature marks the point where metatarsal III emerges from between the other two metatarsals and the posterior ridge, which had been very narrow and sharp, becomes much broader and rounded.

**Fig 21:**
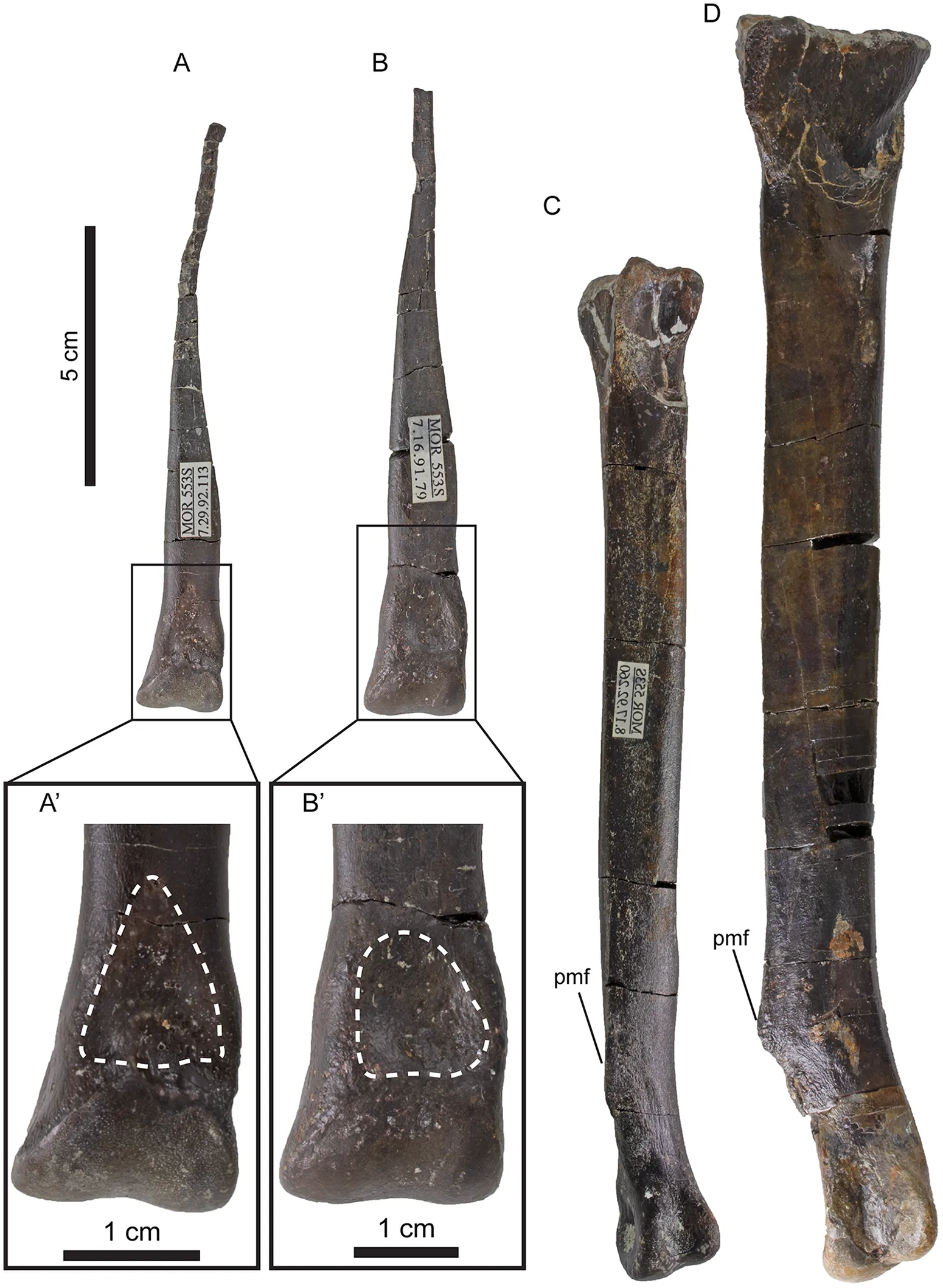
Metatarsal ontogeny and variation of *Troodon formosus.* Left metatarsal III (MOR 553s-7-29-92-113) (A) and left metatarsal III (MOR 553s-7-16-91-79) (B) in anterior views with enlarged anterodistal ends (A’ B’). White dotted line illustrates the outline of the extensor fossa on metatarsal III. Abbreviation: pmf, posteromedial flange. Right metatarsal IV (MOR 553s-8-17-92-260, reversed) (C) and left metatarsal IV (MOR 553s-11-1-01-8) (D) in posterior views, note the variation in the extent of the posteromedial flange.

The distal articular surface is somewhat ginglymoid, with a shallow groove separating the two condyles. Although the condyles have similar mediolateral widths, the medial condyle has a greater radius of curvature. The articular surface extends onto the flexor surface and faces posteriorly. On the flexor surface, the articulation continues proximally as an asymmetric tongue-like projection. A shallow extensor pit lies just proximal to the distal articular surface. This pit is sub-triangular in smaller specimens (e.g., MOR 553S-7-29-92-113, MOR 553S-8-6-9-406) but becomes ovoid in larger ones (MOR 553S-7-16-91-79) where a horizontal rugosity cuts across the top of the fossa (Fig 21). The central portion of this pit remains smooth throughout ontogeny, but its proximal and lateral borders gain a roughened texture in the largest individuals (MOR 553s-7-16-91-79). In all specimens, the distolateral border projects laterally from the edge of the bone. Well-developed collateral ligament pits with roughened surfaces occur on both sides of the distal end. Typically, a second shallower and more longitudinally oriented pit lies proximoposteriorly to the lateral ligament pit.

Throughout ontogeny the proportions of the distal articular surface change. In the smallest specimen MOR 430, the ratio of the mediolateral to anteroposterior dimensions is only 0.56. The average ratio for all other larger specimens is 0.82. The best-preserved specimens (MOR 430, MOR 553s-7-29-92-113, MOR 748, MOR 553s-7-16-91-79, and MOR 553-11-1-01-7) show a consistent trend of increasing mediolateral width to anteroposterior length through ontogeny (Fig 7). In addition to the astragalar condyles and proximal metatarsals, this is another example of relatively narrow articulations in MOR 430.

### MT III comparisons

The short metatarsus of *Troodon* (0.66 femur length) is similar to *Linhevenator* (0.63) [79], but unlike the relatively longer metatarsi of smaller troodontids [1,35,79,84,86,87], and the extreme condition of *Philovenator* (1.24) [51] (Table 1). The arctometatarsalian condition, with metatarsal III being obscured in extensor view proximally and in flexor view distally for *Troodon* is typical of most Late Cretaceous troodontids and some Early Cretaceous taxa such as *Hypnovenator* [116], which is unlike most other paravian taxa [36,90,92,97,102,110,114,121]. In *Philovenator*, the obscured anterior view extends further distally than in *Troodon* to about the midpoint of the metatarsus [51]. In contrast, metatarsal III is not proximally obscured in most Early Cretaceous troodontids, such as *Sinovenator* [37] and *Liaoningvenator* [83]. The extensive degree of posterior coverage of metatarsal III by metatarsals II and IV is greater than in other troodontid specimens (e.g., *Talos*, *Mei*), but this may be a result of ontogenetic differences [56]. The flat to convex anterior edge of metatarsal III in *Troodon* is shared with *Talos* [56] and specimens of *Stenonychosaurus* (e.g., TMP 1998.068.0090) [57], but contrasts with this surface being concave in ‘*L. mcmasterae’* (e.g., TMP 1992.036.0575), *Gobivenator*, MPC-D 100/140, *Linhevenator* [79], and *Philovenator* [8]. The deep longitudinal sulcus on the extensor surface of the proximal metatarsus in *Troodon* is also reported in *Almas* [54] and *Tochisaurus* [123].

The weakly ginglymoid distal articular surface in *Troodon* is shared with *Talos* [56], *Gobivenator* [35], IGM 100/44 [126], and *Urbacodon* [80]. In contrast, *Sinornithoides* [81], dromaeosaurs (e.g., *Deinonychus* [121]), unenlagiines (e.g., *Buitreraptor* [114]), and *Rahonavis* [92] exhibit a more strongly ginglymoid surface. The greater radius of curvature in the medial condyle for *Troodon* is shared with *Talos* [56] and MPC-D 100/140 [125]. The tongue-like projection of the distal articular surface is asymmetric in *Troodon* and *Urbacodon* [80], while it is more symmetrical in *Borogovia* [117] and IGM 100/44 [126]. In *Sinornithoides*, this projection is split with two distinct apexes [81] while the projection is more rounded in *Tochisaurus* [123]. A tongue-like projection is absent for *Philovenator* [52]. *Troodon* shares the distal extensor fossa with other troodontids such as *Philovenator* [51] and *Talos* [56]. The triangular to ovoid outline of this pit in *Troodon* overlaps with the morphology reported for ‘*L. mcmasterae’* (e.g., TMP 1997.133.0008) [8]. The lateral projection of the distolateral edge in *Troodon* is also present in *Talos* [56]*, Linhevenator* [79], MPC-D 100/140 [125], and IGM 100/44 [126]. This lateral projection is more extreme in *Talos*, while it is absent in *Sinovenator* and *Saurornithoides* [56].

### Metatarsal IV

**Fig 22:**
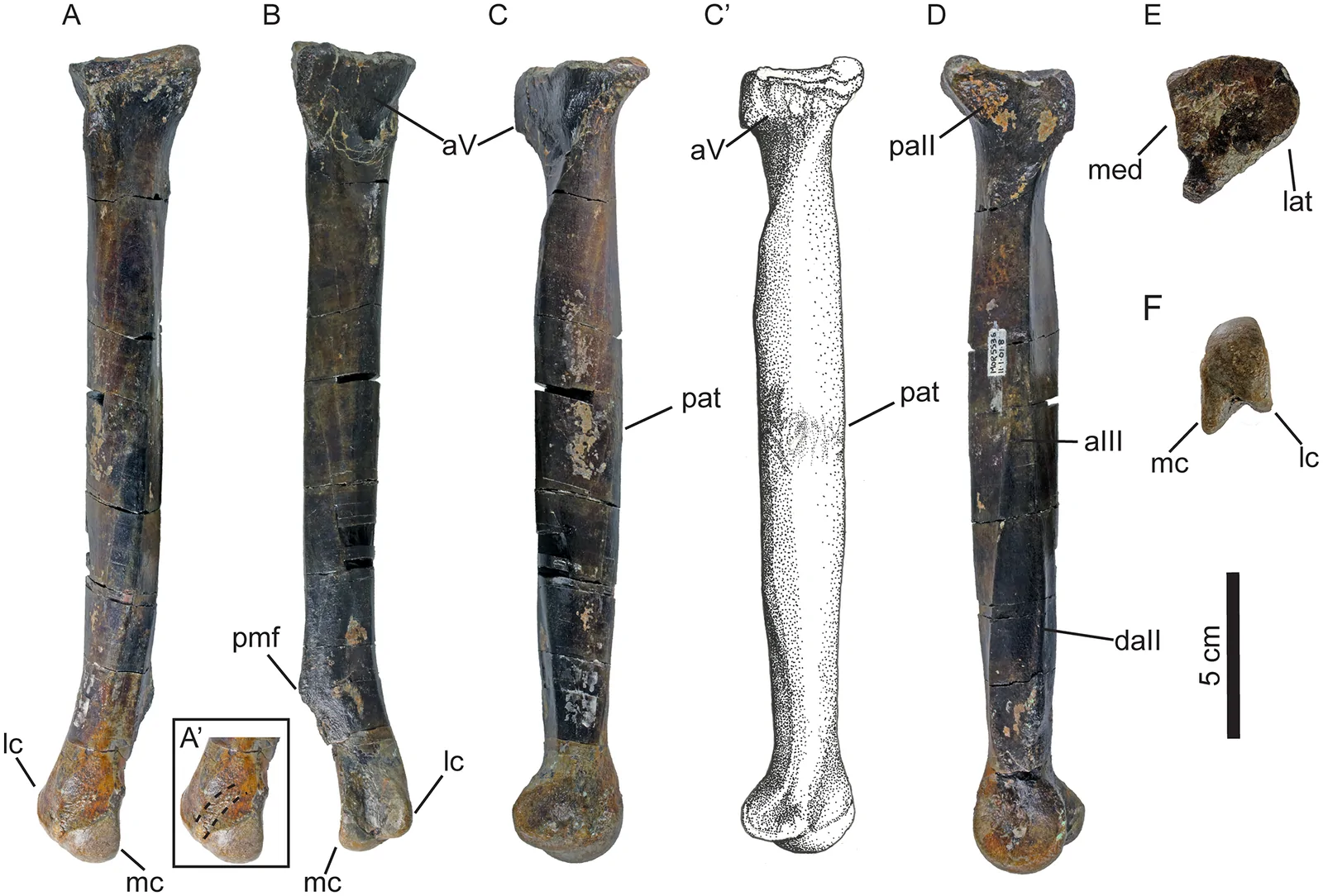
Metatarsal IV of *Troodon formosus.* Right metatarsal IV (MOR 553s-11-1-01-8) of *Troodon* in anterior (A), posterior (B), lateral (C) with corresponding line drawing (C’), medial (D), proximal (E), and distal (D). Distal end of MOR 553s-11-1-01-8 in anterior view (A’) with margins of dorsolateral groove denoted by black dotted line. Abbreviations: aIII, articulation for metatarsal III; aV, metatarsal V articular surface; daII, distal articulation for metatarsal II; lat, lateral side; lc, lateral condyle; med, medial side; mc, medial condyle; paII, proximal articulation for metatarsal II; pat, pathology; pII, proximal articulation for metatarsal II; pmf, posteromedial flange.

The sample for metatarsal IV includes a left from MOR 246–11; a left and right from MOR 430, 563, and 748; and two lefts and two rights from MOR 553 (Fig 22). Metatarsal IV is the stoutest element of the metatarsus. For most of its length, the shaft of metatarsal IV is straight before arcing laterally just prior to the distal articulation. Metatarsal IV is slightly shorter than metatarsal III, with an MT-III to MT-IV length ratio of 1.02. Through ontogeny, metatarsal IV becomes more robust, with an increase in width relative to length (Fig 7 and 21) and an increase in length relative to femur diameter (Fig 10). Two metatarsal IV specimens (MOR 553s-8-17-92-260 and 11-1-01-8) exhibit potential pathologies as raised textured areas on the lateral side of the shafts. The description and comparisons of metatarsal IV for *Troodon* is based on MOR 553s-11-1-01-8 and MOR 748, unless otherwise stated.

The proximal end of metatarsal IV has an anterior expansion and is roughly sub-rectangular in outline, but this varies through ontogeny. The greatest dimension shifts from anterolateral in smaller individuals to mediolateral in larger ones. A posteromedial – projecting flange emerges from the proximal end, contacts the lateral portion of metatarsal III and extends distally. The distomedial extent of this flange is less extreme in smaller specimens [56]. Just distal to the proximal end lies a medially directed triangular facet. In smaller individuals, longitudinal grooves mark the surface whereas larger individuals exhibit a rough, irregular texture. This facet meets a similar structure on metatarsal II and together they exclude metatarsal III from the proximoanterior aspect of the metatarsus (Fig 21).

For much of its length, the shaft has a sub-triangular cross-section. The sub-equal sides face laterally, anteromedially, and posteromedially. Both the lateral and anteromedial sides are convex while the posteromedial side is flat to slightly concave. The two medial sides meet in a sharp angle while the two remaining angles are broadly rounded. The anterior ridge separating the lateral and anteromedial sides follows the curvature of the element. The posterolateral ridge separates the lateral and posteromedial sides and exhibits an irregular texture proximally. This may represent the distal contact of metatarsal V.

The articular surfaces for metatarsals II and III mark much of the anteromedial side beginning about a quarter the length from the proximal end. This surface begins on the posterior edge proximally and expands anteriorly as it continues distally so that at its maximum extent near the base of the distal condyle it occupies about 75% of the anteromedial side. This articular surface is divided in two. A more proximal and anterior portion accommodates metatarsal III while a distal and posterior portion meets metatarsal II. A roughly textured, medial tuberosity marks the posterodistal limit of the metatarsal II articulation (Fig 19).

Beginning at the distal limit of the articular surface for metatarsal II, the shaft of metatarsal IV angles slightly laterally and narrows into an anteroposteriorly thin neck. Two roughened attachment areas sit near the distal articular surface. One, a small bump, sits on the extensor surface just proximal to the articulation. The second extends from the distal extremity of the metatarsal II surface to the posterior medial edge of the distal articulation and consists of a patch of irregularly textured bone. *Troodon* has a deep dorsolateral groove near this region [56]. The distal end bears modest collateral ligament pits on both sides. In outline, the asymmetric distal articulation has a straight medial edge and a convex lateral one. The articulation begins anteriorly as a single, broadly rounded surface then divides posteriorly. After splitting, the medial portion remains along a parasagittal plane while the lateral component splays slightly posterolaterally. The medial portion extends further distally than the lateral one. The articular surfaces of both lateral and medial portions wrap posteriorly around the end of the bone, providing a fair degree of flexion in phalanx IV-1.

### MT IV comparisons

The subequal lengths of metatarsals III and IV in *Troodon* is unique to troodontids [1,3,35,54,56,79,81,83,120,124]. The relative stoutness of metatarsal IV in *Troodon* is shared with other Late Cretaceous troodontids (e.g., *Zanabazar* [3], *Almas* [54], *Talos* [56]). By contrast, the element is relatively slender in *Mei* [1], *Sinusonasus* [88], *Jianianhualong* [86], *Harenadraco* [78], and *Sinovenator* [37]. The anterior expansion of the proximal end in *Troodon* is shared with other taxa such as *Harenadraco* [78] and *Talos* [56], but not *Philovenator* [51]. Both *Troodon* and *Tochisaurus* possess a similar ridge on the anterior side of MT IV [123]. The posteromedial flange for *Troodon* is also reported to have a similar distal extent in *Tochisaurus* [123] and IGM 100/44 [126]. In *Talos*, this flange is present only distally, though this difference may be related to ontogeny [56] given that this flange becomes more exaggerated in larger *Troodon* specimens. The posterolateral ridge in *Troodon* is also present in *Liaoningvenator* [83], *Harenadraco* [78]*, Mei* [87], *Sinovenator* [37], *Sinusonasus* [88], *Philovenator* [51], and *Linhevenator* [79]. This ridge is absent in *Jianianhualong* [86] and *Daliansaurus* [85].

Distally, the lateral curvature of metatarsal IV in *Troodon* is consistent with most paravian theropods [3,36,51,56,79,81,85,90,92,97,114,123,125]. The deep dorsolateral groove present for *Troodon* near the distal end contrasts with *Talos* [56] and MPC-D 100/140 [125] which posses a shallow, pit-like extension of bone that is closed off distally. Unlike *Troodon*, the lateral ligament pit is absent in *Liaoningvenator* [83]. In *Borogovia* [118] and MPC-D 100/140 [125], the medial ligament pit is better developed than in *Troodon*. The relative orientations and extent of the medial and lateral portions of the distal articular surface in *Troodon* is shared with *Talos*, MPC-D 100/140 [125], and *Borogovia* [118]. Unlike *Troodon,* the portions have a more subequal extent in *Sinornithoides* [81].

### Metatarsal V

No Two Medicine Formation specimen preserves a clearly recognizable Metatarsal V. The posterolateral surface of the proximal end of metatarsal IV appears to lack any significant attachment area for metatarsal V. Based on metatarsal IV, *Troodon* had a relatively short metatarsal V that arched away then back to the shaft of metatarsal IV. The textured area situated proximally on the posterolateral edge of metatarsal V may represent this second contact.

**Fig 23:**
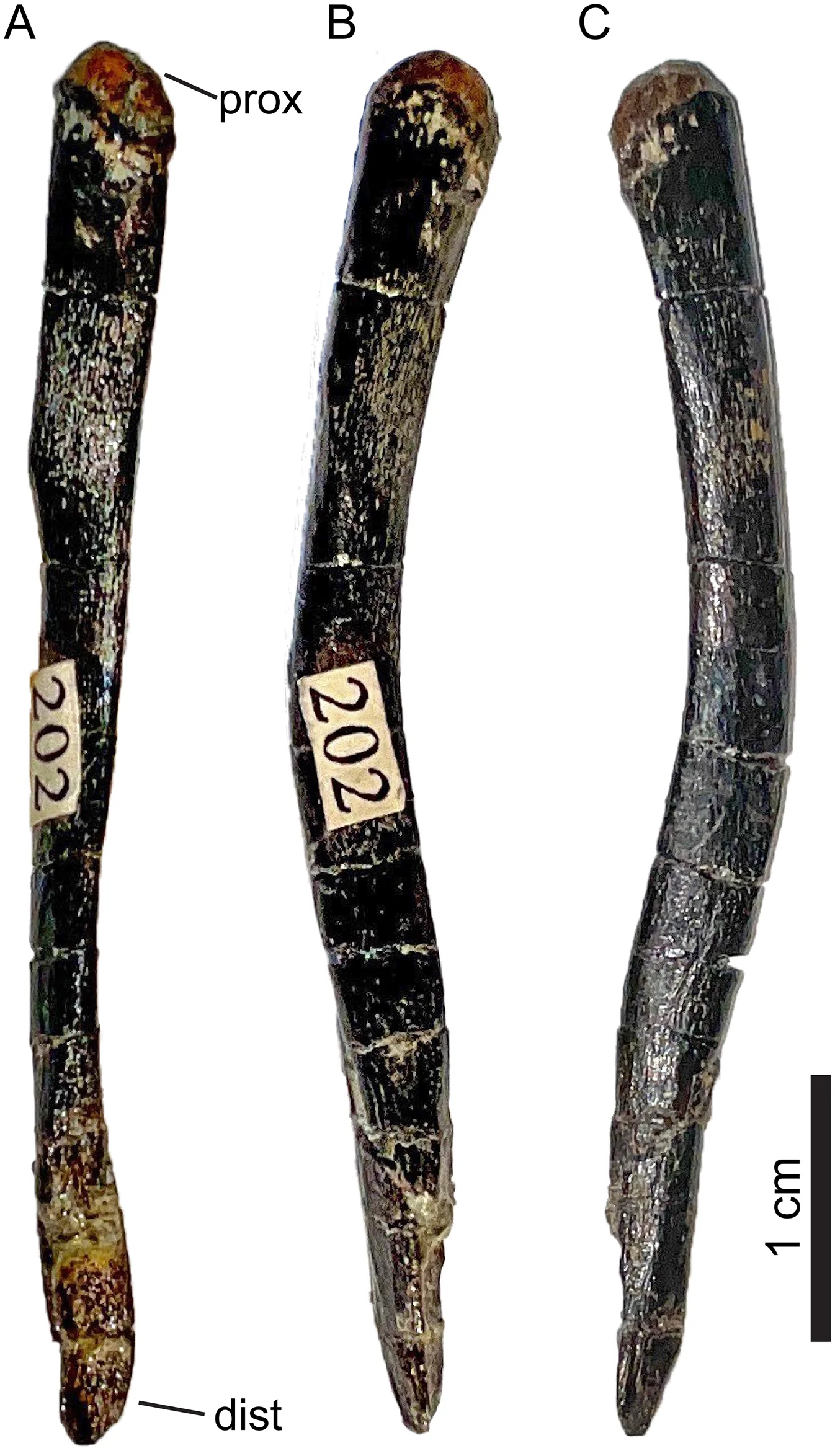
Metatarsal V of *Troodon formosus.* Right metatarsal V (MOR 553s-7-24-91-202) in anterior (A), lateral (B), and medial (C) views. Abbreviations: dist, distal end; prox, proximal end.

One specimen, MOR 553s-7-24-91-202, potentially represents a right metatarsal V (Fig 23). This specimen is an elongate, slightly sigmoidal element that is flattened to ovoid in cross- section. The element twists over its length and measures 53 mm long and 5.2 mm wide. One end terminates in a convex oval articulation, which would potentially be the proximal articulation. A slightly rugose texture is present just distal to this articulation, At the distal end, the edge of the shaft thickens into a flat to slightly concave articular facet that parallels the long axis of the bone.

### Metatarsal V comparisons

MOR 553s-7-24-91-202 closely resembles the metatarsal V from the holotype of *Stenonychosaurus* (NMC 8539), a specimen attributed to ‘*L. mcmasterae’* (TMP 1992.036.0575), and that of *Philovenator* [51,52] In form, MOR 553s-7-24-91-202 would appear to fit subadult metatarsal IV’s such as MOR 553s-8-17-92-260. Consequently, it would measure about 0.27 the length of metatarsal III. This compares favorably to previously reported ratios: *Troodon* (0.30), and *Sinornithoides* (0.38) in [81], and *Philovenator* (0.20) in [52]. In contrast to the reconstruction of Russell [58], the slim, elongate proportions of MOR 553s-7-24-91-202 are similar to metatarsal V in various dromaeosaurids [36,90,121].

### Pes I-1

**Fig 24:**
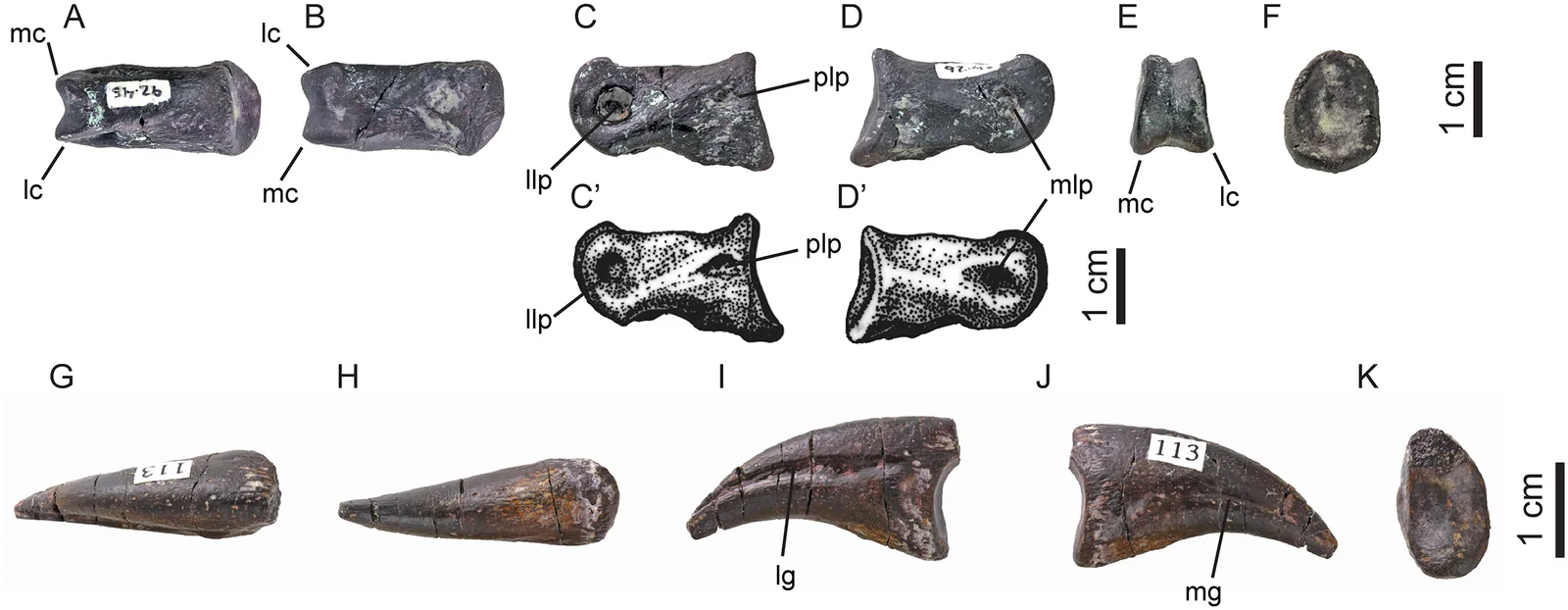
Pedal digit I phalanges from *Troodon formosus.* Left pes phalanx I-1 (MOR 553s-92–45) in extensor (A), flexor (B), lateral (C), medial (D), distal (E), and proximal (F) views, with corresponding line drawings (C’, D’). Left ungual I-2 (MOR 553s-7-18-91-113) in extensor (G), flexor (H), lateral (I), medial (J), and proximal (K) views. Elements are scaled to the proportions of MOR 748. Abbreviation: lc, lateral condyle; lg, lateral ungual groove; mc, medial condyle; mg, medial ungual groove; plp, proximolateral pit.

Of the three specimens of I-1 available, two come from associated skeletons, MOR 430 and 748, and a third from Jack’s Birthday Site, MOR 553s-7-21-92-45 (Fig 24) – the basis of the description and comparisons here. The length of I-1 relative to III-1 is 0.47 in MOR 430 and 0.48 in MOR 748 (Table 2). The proximal articulation is smoothly concave and ovoid in outline with a proximoventral heel. No dorsoventral ridge is present on the proximal articulation, as with all first pedal phalanges in *Troodon*. A pit is present at the proximolateral surface of I-1. The lateroventral surface has a prominent rugosity. The distal ginglymoid articulation is nearly symmetric with a slightly dorsoventrally deeper medial condyle. The limited distal articulation extends just far enough dorsally to reach the extensor surface of the shaft. This surface is dorsoventrally deeper than mediolaterally broad and sweeps an arc of roughly 180^o^. The distal articulation sits at an angle relative to the shaft such that the first digit would have projected slightly anteromedially away from the metatarsus. The lateral ligament pit is much deeper than the medial one. As is typical for ungual-bearing phalanges, the dorsal surface between the pits is narrowed. In comparison to other non-penultimate phalanxes, the narrowing provides greater exposure of the collateral pits dorsally.

**Table 2:** Pedal phalanx length proportions for troodontids.

Taxon	Specimen	III-1 Length (mm)	I-1/III-1	II-1/III-1	II-2/III-1	III-2/III-1	III-3/III-1	IV-1/III-1	IV-2/IV-1	IV-4/IV-1
Troodon	MOR 430	20.8	0.47	0.89	–	0.81	0.71	0.61	0.98	–
Troodon	MOR 563	39.4	–	–	0.61	0.74	0.68	0.59	0.89	0.85
Troodon	MOR 748	49.2	0.48	0.86	–	0.69	0.61	–	–	–
Saurornithoides	AMNH FR 6515	33	0.48	0.83	–	0.73	–	0.62	–	–
Philovenator	IVPP V10597	14.5	–	0.72	0.58	0.83	–	0.76	0.95	0.82
Sinornithoides	IVPP V9612	25.3	0.42	0.83	0.47	0.77	0.63	0.71	0.82	0.71
	IGM 100/44	29	0.45	0.86	0.59	0.59	0.41	0.66	–	–
Mei	DMNH D2154	7.6	–	–	–	–	–	–	0.76	0.62
Talos	UMNH VP 19479	35.58	0.53	0.91	0.62	0.73	0.72	0.64	0.93	0.8
Hesperornithoides	WYDICE-DML-001	26.3	–	–	–	0.66	–	–	–	–
Linhevenator	LH V0021	40	0.58	0.7	–	0.73	0.625	0.88	0.51	0.49
	MPC-D 100/140	25.6	0.44	0.8	0.58	0.7	0.66	0.66	0.9	0.82
Borogovia	ZPAL MgD-1/174	–	–	–	–	–	–	–	0.94	0.89
Daliansaurus	DNHM D2885	–	–	–	–	–	–	–	0.93	0.71

Pedal phalanx length proportions for *Troodon* and other troodontids [1–3,52,79,84,85,117].

**Fig 25:**
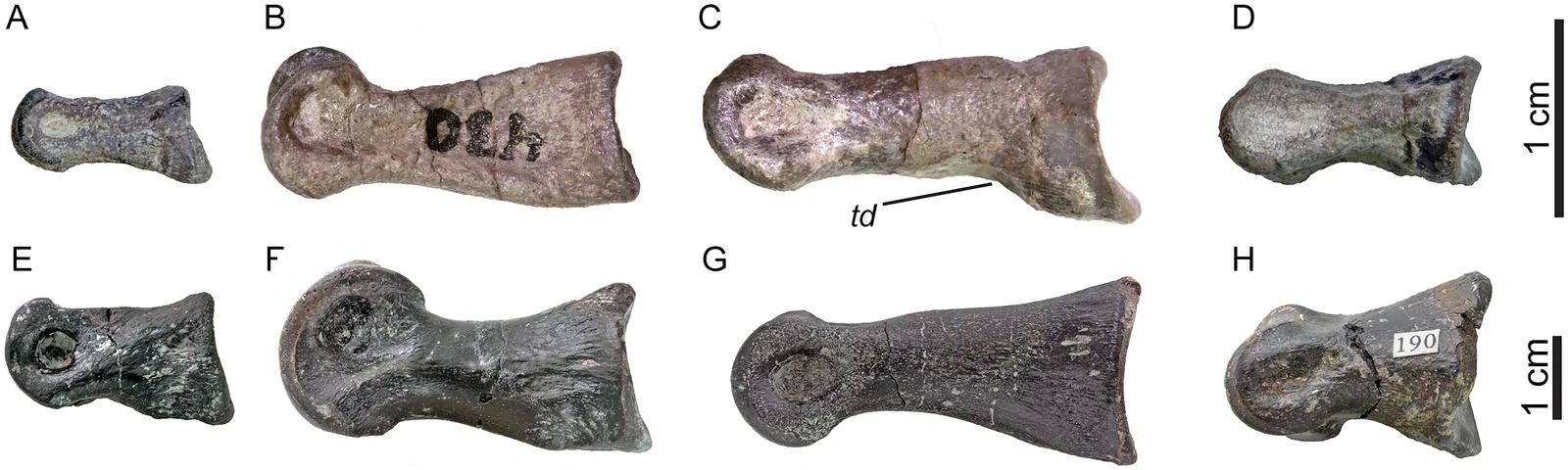
Ontogenetic changes of pedal phalanges for *Troodon formosus.* First pedal phalanges in lateral view of *Troodon* from a small juvenile, MOR 430 (A-D), and adult individuals, MOR 553s (E-H). Digits I-1 (A,E), II-1 (B,F), III-1 (C,G), and IV-1 (D,H). Abbreviation: *td*, taphonomic deformation.

I-1 is somewhat elongate in juvenile MOR 430, but the shaft is stouter in the two adult specimens (Fig 25). Midshaft dimensions are only 30% of the greatest length in MOR 430 but about 40% in the larger specimens. While the shaft of the juvenile element is devoid of any significant features, a raised area with prominent attachment scarring occurs lateroventrally just distal to the proximal end in the larger specimens. A small pit occurs laterally just dorsal to the limits of this textured area.

### Pes I-1 comparisons

Phalanx I-1 for *Troodon* is relatively stout when compared to *Sinornithoides* [81], MPC-D 100/140 [125], and *Talos* [56]. The relative length of I-1 is somewhat consistent across Troodontidae. The I-1 to III-3 length ratio for *Troodon* (~0.47) is similar to *Saurornithoides* (0.48) [58], *Sinornithoides* (0.42) [84], IGM 100/44 (0.45) [126], *Talos* (0.53) [56], *Linhevenator* (0.58) [79], and MPC-D 100/140 (0.44) [125] (Table 2). The non-ginglymoid proximal articulation and ginglymoid distal articulation in *Troodon* is shared with other troodontids, dromaeosaurids [36,97], unenlagiines [127,128], and *Rahonavis* [92]. The proximolateral pit in *Troodon* is shared with *Talos*. The proximoventral heel for *Troodon* is also present in MPC-D 100/140 [125] and *Talos* [56] (though the heel is more pronounced in *Talos*), as well as dromaeosaurids [36,97], unenlagiines [127,128], and *Rahonavis* [92]. In contrast to *Troodon*, the proximal articular surface is more asymmetrical in MPC-D 100/140 [125] and sub-triangular with a proximodorsally-projecting flange in *Harenadraco* [78]. The distal articular surface of *Troodon* being dorsoventrally deeper than mediolaterally broad is shared with MPC-D 100/140 [125] and *Talos*. Both *Troodon* and *Philovenator* [51,52] present evidence in the distal articulation of digit I being oriented anteromedially. This contrasts with *Sinornithoides*, where the angle of articulation would have allowed the first digit to project posteromedially [81]. The distal articulation for *Troodon* is similar to that of MPC-D 100/140 [125], with both having a slightly deeper medial condyle. In MPC-D 100/140 (80), *Sinornithoides* [81], *Shri devi* [36] and *Velociraptor* [97] the distal articulation projects further dorsally relative to the midshaft than in *Troodon*, *Talos* [56], or *Rahonavis* [92]. The deeper lateral collateral ligament pit in *Troodon* is shared with *Saurornithoides* [3] and MPC-D 100/140 [125].

### Pes II-1

**Fig 26:**
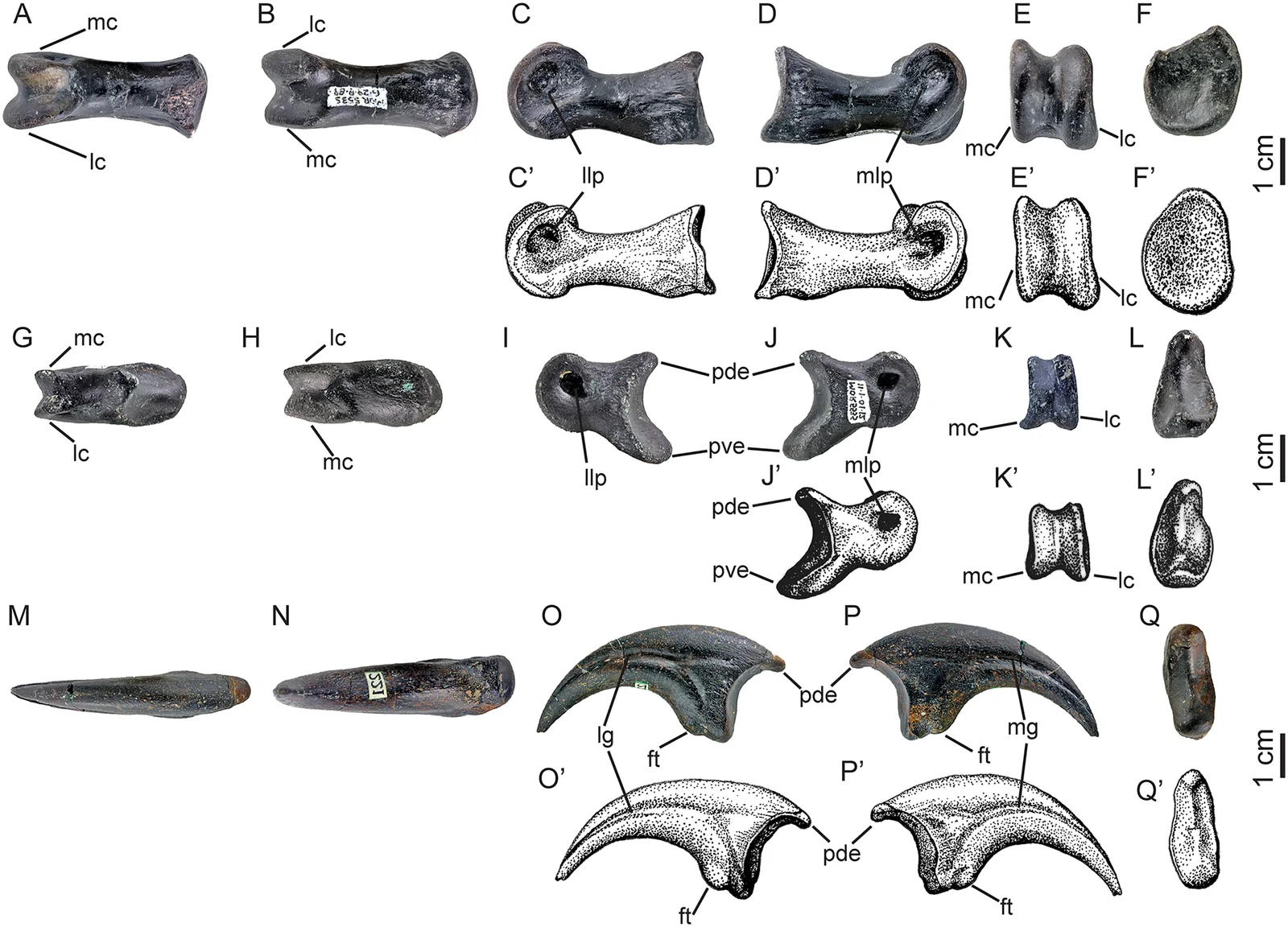
Pedal digit II phalanges of *Troodon formosus.* Left pedal phalanx II-1 (MOR 553s-6-29-9-89) in extensor (A), flexor (B), lateral (C), medial (D), distal (E), and proximal (F) views, with corresponding line drawings (C’-F’). Right pedal phalanx II-2 (MOR 553s-11-1-01-12, reversed) in extensor (G), flexor (H), lateral (I), medial (J), distal (K), and proximal (L) views, with corresponding line drawings (J’-L’). Right ungual phalanx II-3 (MOR 553s-8-12-92-221, reversed) in extensor (M), flexor (N), lateral (O), medial (P), and proximal (Q) views, with corresponding line drawings (O’-Q’). All phalanges are scaled to the relative proportions of MOR 748. Abbreviations: ft, flexor tubercle; lc, lateral condyle; lg, lateral ungual groove; llp, lateral ligament pit; mc, medial condyle; mg, medial ungual groove; mlp, medial ligament pit; pde, proximodorsal extension; pve, proximoventral extension.

**Fig 27:**
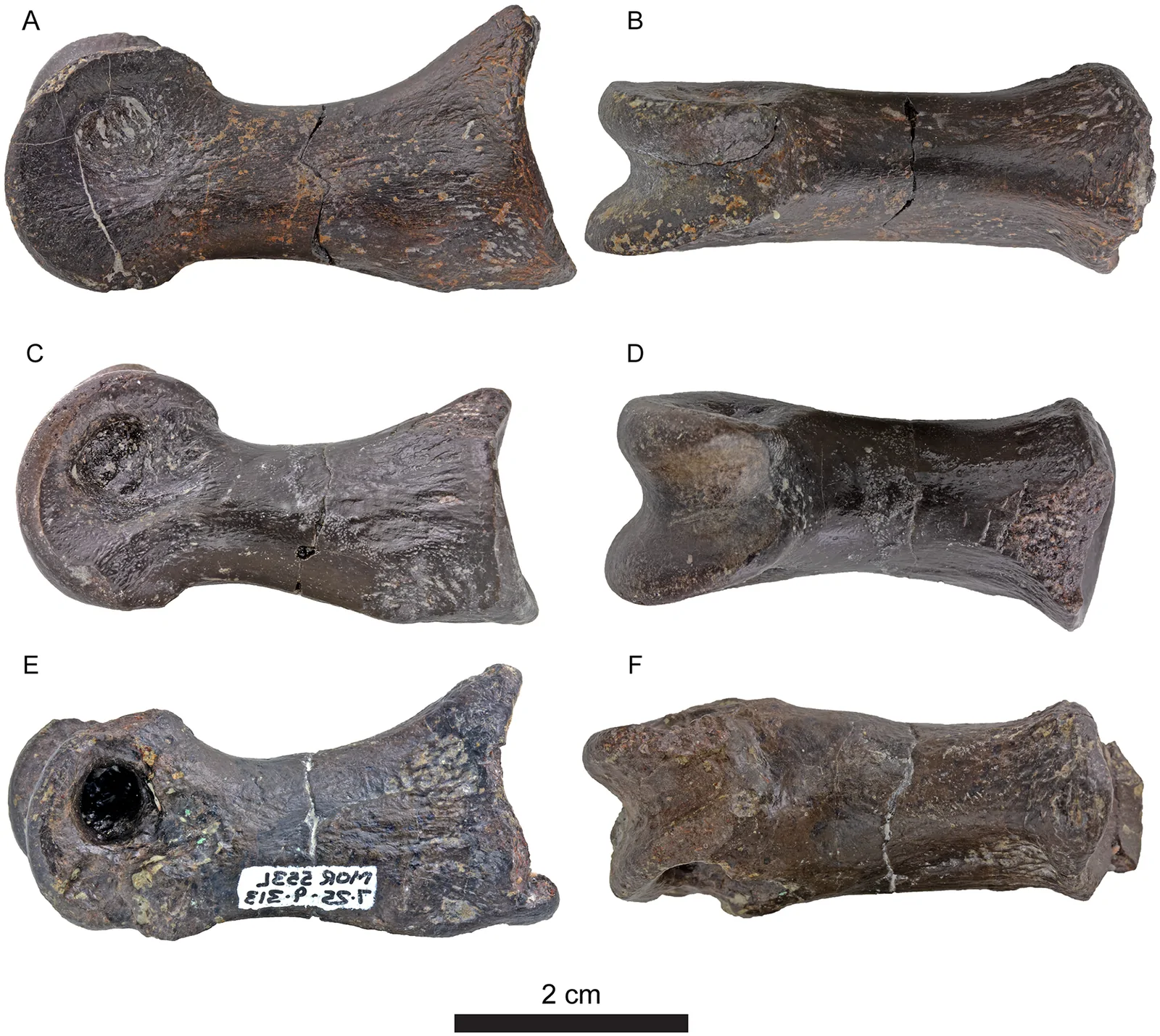
Variation and pathology in pedal digit II-1 for *Troodon formosus.* Pedal digits II-1 for *Troodon* in lateral (A, C, E) and extensor (B, D, F) views. MOR 553s-7-6-91-13 (A, B), the typical morphotype. MOR 553s-6-29-9-89 (C, D), the robust morphotype. MOR 553l-7-25-9-313 (reversed) (E, F), a pathologic pes phalanx.

A total of eleven phalanx II-1 specimens are known for *Troodon* from the Two Medicine sample. These include MOR 430, 563, 748, 656, and six specimens from MOR 553 (Fig 26). Two of these II-2 specimens exhibit pathologies. MOR 553s-7-6-91-13 bears an abnormal, roughly textured rise just dorsal to the proximal articular surface. In contrast, MOR 553l-7-25-9-313 shows extensive pathologic remodeling just proximal to the distal articulation (Fig 27). The description and comparison of pes phalanx II-1 is based on MOR 553s-6-29-9-89, unless otherwise stated.

The shafts of the digit II phalanges in *Troodon*, unlike those of all other phalanges, are dorsoventrally taller than wide. The proximal articulation of II-1 is smoothly concave and ovoid with a proximoventral heel. A roughly textured area is present on the ventral surface proximally, which is slightly bisected longitudinally and bears a slight lateroventral depression. This rugosity, weakly represented in MOR 430 (the small juvenile), becomes increasingly more pronounced through ontogeny. A second variably textured area occurs just distal to the proximal articulation as a smaller, slight depression facing laterodorsally. The strongly ginglymoid distal articulation consists of a broadly arcing, larger lateral condyle and a more tightly curved medial condyle. The medial condyle sits proximal and dorsal to its larger counterpart. The articular surface of both condyles forms an arc of about 215^o^ extending proximally onto the extensor surface. This construction results in phalanx II-2 having a range of movement of about 155^o^ and being fully retractable onto the dorsal surface of II-1. Further, the offset condyles angles phalanx II-2 from anterolaterally when extended to posteromedially when fully flexed. On the distal end, the smaller medial ligament pit consists of two weakly separated depressions, whereas the lateral ligament pit is deeper and undivided.

There is a slight increase in the robustness of II-1 through ontogeny, though this trend is not significantly different from isometry and is weakly correlated (Fig 7). The phalanx II-1/III-1 length ratio is about 0.89 in MOR 430 and 0.86 in MOR 748 (Table 2). In contrast to other dimensions, the dorsoventral midshaft depth remains relatively unchanged. Two specimens stand out as consistently more robust than others of similar greatest length: MOR 748 (the specimen intimately associated with an egg clutch) and MOR 553s-6-29-9-89 (Fig 27). These likely represent fully mature individuals.

### Pes II-1 comparisons

The length of II-1 relative to III-1 (~0.8) for *Troodon* is generally similar to that of most other troodontids [52,56,58,79,84,125,126] (Table 2). Unlike *Troodon*, MPC-D 100/140, dromaeosaurids such as *Deinonychus* [106], and unenlagiines such as *Neuquenraptor* [127] have a divided proximal articular surface on II-1 [125]. *Troodon*, *Saurornithoides* [3], *Borogovia* [117], and MPC-D 100/140 [125] all have a deeper lateral pit. The medial pit, by contrast, is deeper in IGM 100/140 [126] and *Harenadraco* [78]. *Troodon*, *Borogovia* [117], and *Harenadraco* [78] all have a larger lateral distal condyle. A ginglymoid distal articulation that extends dorsal to the shaft is widespread amongst Troodontidae [3,35,51,56,83,85,117,125,126] as well as dromaeosaurids [36,97,106] and unenlagiines [114,127,128].

### Pes II-2

Seven specimens of pedal phalanx II-2 (Fig 26) are represented for *Troodon* (MOR 430, 553, 563). The description and comparison here is based on MOR 553s-11-1-01-12, unless otherwise stated. Pes phalanx II-2 is relatively short, being about 61 percent the length of pes phalanx III-1 in MOR 563 (Table 2). The laterally compressed proximal articular for *Troodon* possesses prominent proximodorsal and proximoventral extensions. Two grooves divided by a midline ridge are present on this articulation. The medial groove of the articular surface sits slightly dorsal to the lateral one. The orientation of this ridge and the corresponding groove of phalanx II-1 would have largely restricted movement of II-2 to just a dorsoventral plane. An irregular bone texture marks the surface of the phalanx just distal and ventral to the proximoventral extension. The laterally compressed distal articulation is ginglymoid and sweeps through an angle of about 180 degrees. The distal articulation extends beyond the dorsal edge of the shaft, with the lateral condyle just slightly larger than the medial. The lateral ligament pit is larger than the medial pit. The element becomes slightly more robust through ontogeny.

### Pes II-2 comparisons

The II-2 to III-1 length ratio for *Troodon* (0.61, MOR 563) is similar to IGM 100/44 (0.59) [126], *Talos* (0.62) [56], *Philovenator* (0.58) [52], and MPC-D 100/140 (0.58) [125]. In comparison, *Sinornithoides* has the smallest value of 0.47 [84] (Table 2). The laterally compressed proximal articulation in *Troodon* and *Talos* [56] is unlike *Borogovia* [117] and MPC-D 100/140 [125]. The midline ridge, proximodorsal and proximoventral extensions on the proximal articular surface for *Troodon* is consistent with most other troodontids [52,56,80,85,118,125]. However, the proximoventral extension in *Troodon* is relatively short compared to *Harenadraco* [78].The proximodorsal projection is not as pronounced in *Sinornithoides* [81], IGM 100/44 [126],MPC-D 100/140 [125], *Rahonavis*, dromaeosaurids [36,97,106], and unenlagiines [114,127,128] as it is in *Troodon*. The laterally compressed ginglymoid distal articulation of *Troodon* is shared with other deinonychosaurs ([36,56,81,114,125,127]). Unlike *Troodon*, the distal articulation does not extend beyond the dorsal edge in *Saurornithoides* [3].

### Pes III-1

**Fig 28:**
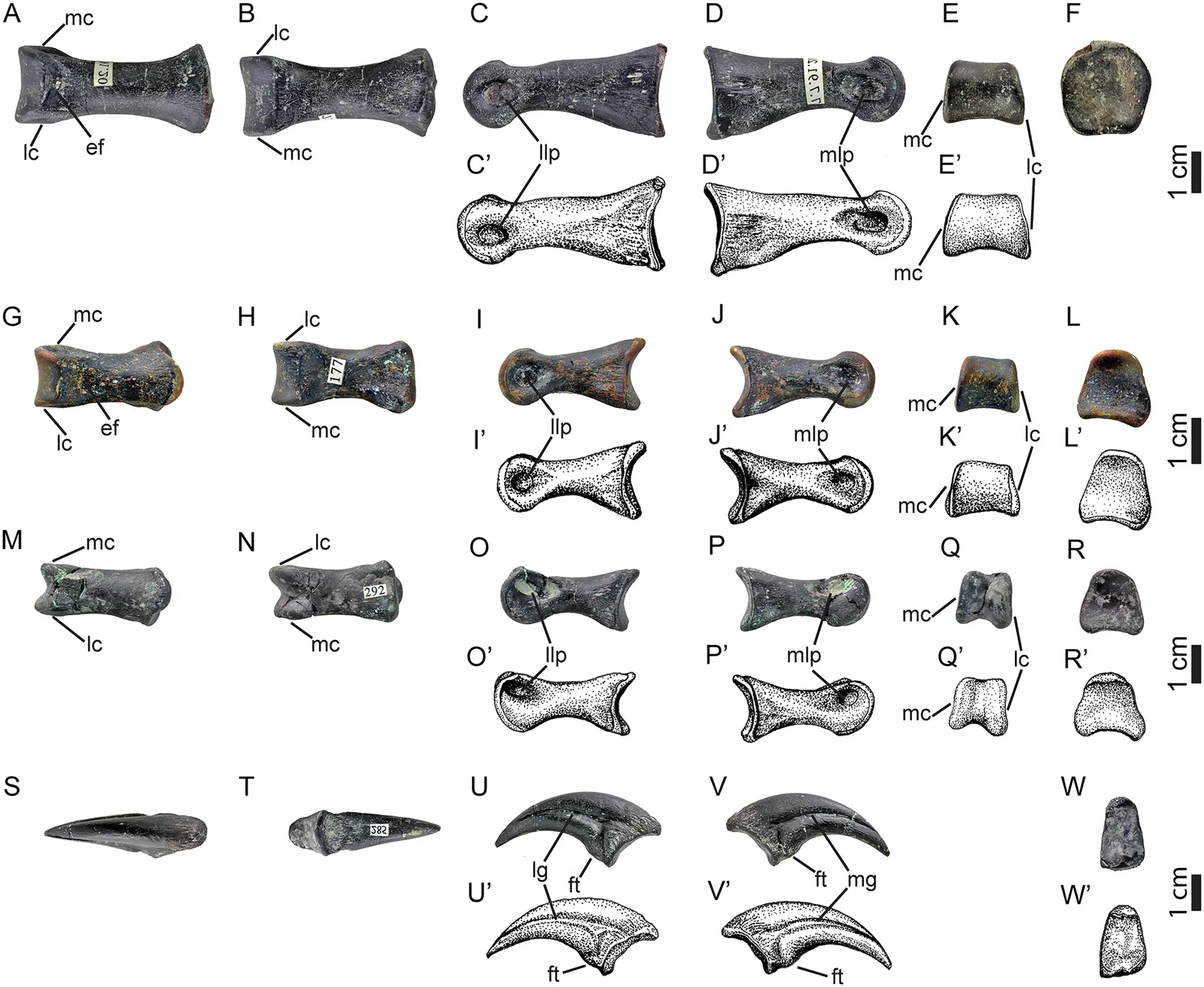
Digit III pedal phalanges of *Troodon formosus.* Right pedal phalanx III-1 (MOR 553s-7-7-91-20, reversed) in extensor (A), flexor (B), lateral (C), medial (D), distal (E), and proximal (F) views, with corresponding line drawings (C’-E’). Left pedal phalanx III-2 (MOR 553s-7-23-91-177) in extensor (G), flexor (H), lateral (I), medial (J), distal (K), and proximal (L) views, with corresponding line drawings (I’-L’). Pedal phalanx III-3 (MOR 553s-8-2-91-292) in extensor (M), flexor (N), lateral or medial view 1 (O), lateral or medial view 2 (P), distal (Q), and proximal (R) views with corresponding line drawings (O’-R’). Pedal ungual phalanx III-4 or IV-5 in extensor (S), flexor (T), lateral (U), medial (V), and proximal (W) views, with corresponding line drawings (U’-W’). Phalanges are scaled to the proportions of MOR 748 and MOR 563 (a large juvenile). Abbreviations: ef, extensor fossa; ft, flexor tubercle; lc, lateral condyle; lg, lateral ungual groove; llp, lateral ligament pit; mc, medial condyle; mg, medial ungual groove; mlp, medial ligament pit.

**Fig 29:**
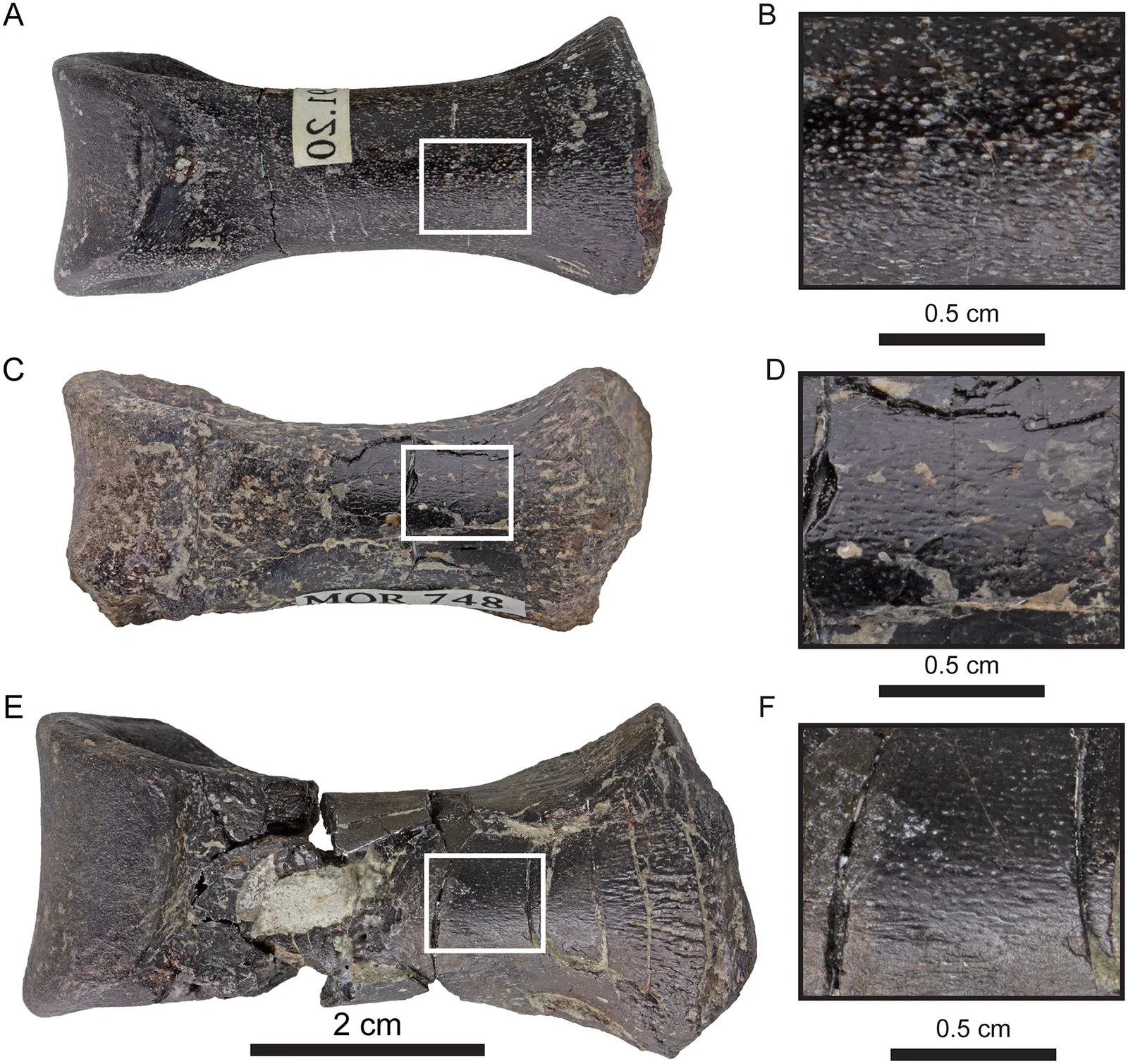
Pes phalanx III-1 variation in *Troodon formosus.* Pes phalanges III-1 in dorsal (A, C, E) views with close-up of proximodorsal surfaces (B, D, E) to highlight differences in bone surface porosity. MOR 553s-7-7-91-20 (A, B; right reversed), MOR 748 (C, E; left), MOR 553l-11-1-01-14 (E, F; right reversed). White box denotes the region from where close-up image of element comes.

The sample for phalanx III-1 (Fig 28) of *Troodon* consists of seven specimens from MOR 430, 563, 553, and 748. One phalanx, MOR 553s-8-11-92-209, bears a potentially pathologic 1 mm wide shallow groove marking the dorsal portion of the proximal articulation. MOR 748 has a phalanx III-1, that, like its II-1, is more robust than other specimens of similar length (Fig 29). Its proportions come closer to the much longer MOR 553l-11-1-01-14. Similar in size to MOR 748, MOR 553s-7-7-91-20 and MOR 553s-8-11-92-209 have a spongy surface texture suggesting immaturity. The description and comparisons for pes phalanx III-1 is based on MOR 553s-8-11-92-209, unless otherwise stated.

The proximal articulation of phalanx III-1, the largest phalanx of the pes, consists of a smooth shallow concavity. In proximal view, the ventral edge is straight while the remainder arcs smoothly in a subcircular outline, except for a sharp angle dorsomedially. The mid-shaft of a typical III-1 is slender with a sub-circular cross-section. The ratio of the midshaft width to length of the element is generally constant through ontogeny (Fig 7). A rise of roughly textured bone is present proximally from the ventromedial to ventrolateral sides. On some specimens, a small longitudinal groove marks the ventromedial portion of this textured surface. A shallow, broad extensor fossa marks the dorsal surface of the shaft just proximal to the distal articulation. The collateral ligament pits are subequal, large and deep. The distal articular surface is mediolaterally broader than tall. The distal articular surface arcs through over 180^o^ and is slightly deeper medially. Ventrally, the proximal edge of the distal articular surface is nearly straight. Dorsally, the greatest proximal extent of the distal articular surface is slightly lateral of midline. This smooth, undivided, surface is not ginglymoid. In the smallest specimens, MOR 430 and 563, the articular surface is concave. Through ontogeny this decreases, so that the articulation in the largest specimens (e.g., MOR 553l-11-1-01-14) is virtually flat mediolaterally and resembles half a cylinder. Through ontogeny, the length of phalanx III-1 slightly decreases relative to femur diameter (Fig 10).

### Pes III-1 comparisons

The undivided, smoothly arched, shallow concavity, of the proximal articulation in *Troodon* is shared with *Mei* [1] and MPC-D 100/140 [125], but not IGM 100/44 [126], *Deinonychus* [106], and *Neuquenraptor* [127] where the surface is weakly divided. As in *Troodon*, an extensor fossa is also present in *Saurornithoides* [3], *Liaoningvenator* [83], and *Talos* [56], although it is only “slightly expressed” in *Talos*. The collateral ligament pits are subequal, large and deep, in *Troodon*, IGM 100/44 [126], *Saurornithoides* [3], *Borogovia* [117], and *Linhevenator* [51], but unlike *Talos* [56], where the lateral pit is deeper. Like *Troodon*, the distal articulation of *Liaoningvenator* [83] is dorsoventrally deeper medially whereas this articulation is deeper laterally in *Talos* [56] and MPC-D 100/140 [125]. The distal articulation for *Troodon* is relatively broader than in *Borogovia* [117]. This non-ginglymoid surface in *Troodon* is shared with MPC-D 100/140 [125], *Borogovia* [117], and *Saurornithoides* [3], but contrasts with the ginglymoid articulation for *Mei* [1], *Liaoningvenator* [83], IGM 100/44 [126], and *Hypnovenator* [116], dromaeosaurids such as *Velociraptor mongoliensis* and *Deinonychus* [97,106], and unenlagiines [89,127].

### Pes III-2

A sample of five specimens for phalanx III-2 (Fig 28) for *Troodon* exists, coming from MOR 430, 553, 563, and 748. The description and comparisons for pes phalanx III-2 is based on MOR 553s-7-23-91-177, unless otherwise stated. Phalanx III-2 represents the third longest element in the pes after III-1 and II-1, being about 69% the length of phalanx III-1 in MOR 748 and about 81% in MOR 430 (Table 2). Its proximal articulation has a roughly triangular outline with a gently concave ventral side. This proximal articulation is completely undivided andstrongly concave dorsoventrally. The dorsal-most portion projects proximally. This articular surface matches the half-cylinder like distal end of III-1. Medial and lateral irregular ridges with slightly roughened surfaces mark the proximoventral portion of the shaft and define a slight ventral concavity. These ridges moderately influence the outline of the proximal articulation. The shaft is mediolaterally broader than dorsoventrally deep. A shallow extensor fossa is present proximal to the distal articulation. Subcircular collateral pits are well developed with the lateral pit being deeper than the medial one. The broad distal articulation is only weakly concave transversely. Its ventral proximal limit is nearly straight while its dorsal one angles slightly medially. As in III-1, the distal articulation is slightly deeper medially.

### Pes III-2 comparisons

Phalanx III-2 of *Troodon* is relatively broader than III-2 of *Borogovia* [117]. The range of relative lengths of III-2 to III-1 in *Troodon* (0.81 in MOR 430, 0.69 in MOR 748) is similar to *Saurornithoides* (0.73) [58], *Philovenator* (0.83) [52], *Sinornithoides* (0.77) [84], IGM 100/44 (0.59) [126], *Talos* (0.73) [56], *Hesperornithoides* (0.66) [2], *Linhevenator* (0.73) [79], and MPC-D 100/140 (0.7) [125] (Table 2). The proximal articulation of *Troodon* is similar in outline (sub-triangular with ventral concavity) to MPC-D 100/140 [125] and *Borogovia* [117]. The undivided proximal articulation in *Troodon* is shared with *Borogovia* [117], *Harenadraco* [78], and MPC-D 100/140 [125], in contrast to the divided surface in dromaeosaurids including *Velociraptor mongoliensis* and *Deinonychus* [97,106] and unenlagiines (e.g., *Neuquenraptor* [127]). An extensor pit is present in *Troodon*, *Saurornithoides* [3], *Harenadraco*, and MPC-D 100/140 [125], but not in *Talos* [56]. The lateral ligament pit is deeper than the medial one in both *Troodon* and *Talos* [56]. The weak concavity of the distal articulation in *Troodon* is similar to that of *Harenadraco* [78], *Saurornithoides* [3], *Talos* [56], and MPC-D 100/140 [125]. As in III-1, the distal articulation is slightly larger medially for *Troodon*, while the lateral side is larger in *Talos* [56] and MPC-D 100/140 [125].

### Pes III-3

At least three specimens (from MOR 430, 553, 563) exist for phalanx III-3 (Fig 28) for *Troodon*. None of the three Two Medicine specimens were found in articulation, consequently their interpretation as lefts could be wrong. The description and comparisons of phalanx III-3 is based on MOR 553s-8-2-91-292, unless otherwise stated. The proximal half of III-3 is virtually identical to that of III-2. The shaft of the phalanx angles slightly lateral to the plane of the proximal articulation. Unlike III-1 and III-2, III-3 lacks an extensor fossa. Further, the collateral ligament pits are placed high on the phalanx, close to the dorsal edge. These pits are subequal in depth with an elongate shape that is more open proximally. The distal articulation is strongly ginglymoid and wider than the shaft. The slightly deeper lateral condyle also extends farther distally than the medial condyle. Through ontogeny there is an overall increase in the element’s robustness, particularly in the dorsoventral dimension. The relative length of phalanx III-3 to III-1 ranges from 0.71 in MOR 430 to 0.61 in MOR 748 (Table 2).

### III-3 comparisons

The proximal articulation of III-3 is concave in *Troodon* as in *Mei* [1], MPC-D 100/140 [125], and *Talos* [56]. Relative to its length, III-3 of *Troodon* is broader than that of *Borogovia* [117] and MPC-D 100/140 [125], but similar in form to that of *Talos* [56]. For *Troodon*, *Sinornithoides* [58], *Talos* [56], *Linhevenator* [79], and MPC-D 100/140 [125], phalanx III-3 is about 0.6 to 0.7 the length of III-1. By contrast, this element is much shorter for IGM 100/44 (0.41) [126] (Table 2). Both *Troodon* and *Talos* lack an extensor fossa for this element [56]. The dorsal placement of the collateral ligament pits for *Troodon* is shared with *Borogovia* [118], MPC-D 100/140 [125], *Linhevenator* [79], and *Talos* [56]. The lateral ligament pit is deeper than the medial in *Talos* [56], unlike the subequal depths in *Troodon*. The elongate ligament pits in *Troodon* are similar to *Talos* [56] and MPC-D 100/140 [125], but unlike *Borogovia* [118] where the lateral pit is elongate and the medial is subcircular. The distal articulation is ginglymoid in *Troodon*, *Borogovia* [117], *Talos* [56], MPC-D 100/140 [125], and *Liaoningvenator* [83] as well as dromaeosaurids (e.g., *Deinonychus* [106]) and unenlagiines (e.g., *Neuquenraptor* [127]), but not in *Harenadraco* [78]. *Troodon*, *Talos* [56], and *Hypnovenator* [116] have a slightly deeper and more distally extending lateral condyle. In MPC-D 100/140 [125], the condyles have a subequal distal extent (Fig 28).

### Pes IV-1

**Fig 30:**
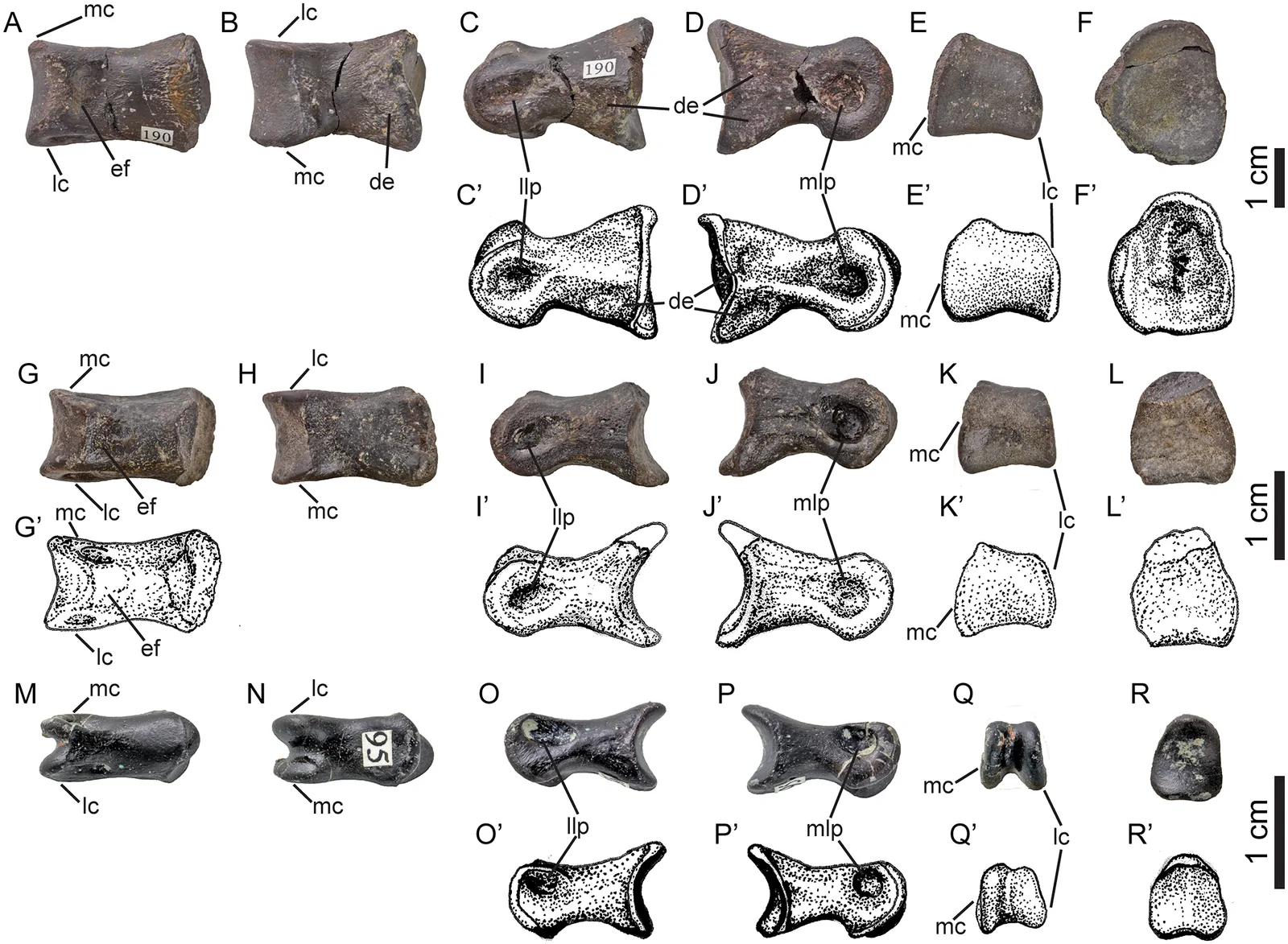
Digit IV phalanges for *Troodon formosus.* Left pedal phalanx IV-1 (MOR 553s-7-24-91-190) in extensor (A), flexor (B), lateral (C), medial (D), distal (E), and proximal (F) views, with corresponding line drawings (C’-F’). Left pedal phalanx IV-2 (MOR 563) in extensor (G), flexor (H), lateral (I), medial (J), distal (K), and proximal (L) views, with corresponding line drawings (G’, I’-L’). Left pedal phalanx IV-4 (MOR 553s-7-28-92-95) in extensor (M), flexor (N), lateral (O), medial (P), distal (Q), and proximal (R) views with corresponding line drawings (O’-R’). Abbreviations: de, proximal depressions; ef, extensor fossa; lc, lateral condyle; lg, lateral ungual groove; llp, lateral ligament pit; mc, medial condyle; mg, medial ungual groove; mlp, medial ligament pit.

The sample of phalanx IV-1 (Fig 30) consists of one specimen from MOR 430, five from MOR 553, and one from MOR 563. The description and comparisons of pes phalanx IV-1 is based on MOR 553s-7-24-91-190, except where noted. Phalanx IV-1 is a short, stout and asymmetric element, being about 60% the length of phalanx III-1 in MOR 430 and MOR 563 (Table 2). The proximal end consists of a dorsoventrally tall single concavity. The articular surface is smoothly outlined in juveniles but becomes more irregular through ontogeny. A small lateral and a large ventromedial projection extend proximally from the articular surface. MOR 553s-8-9-92-190 and 8-11-92-213 bear possibly pathologic irregular grooves and depressions in the center of the otherwise smooth articulation. From the relatively tall proximal end, the shaft pinches down to about half as deep just prior to the distal articulation. Four irregular depressions, likely ligament or tendon attachment scars, mark the shaft just distal to the proximal articulation. These occur dorsomedially, ventromedially, ventrally, and ventrolaterally. An extensor fossa sits just proximal to the distal articular surface. The distal articulation for *Troodon* is not ginglymoid, but slightly concave about the midline, and encompasses a dorsoventral arc of just over 180^o^. It also is much taller medially than laterally. The proximoventral limit of the articular surface is nearly straight. The medial ligament pit is larger than the lateral.

### Pes IV-1 comparisons

Phalanx IV-1 is relatively stout in *Troodon*, *Borogovia* [117], and *Talos* [56], compared to the more elongate element in Early Cretaceous troodontids (e.g., IGM 100/140 [126], *Sinornithoides* [81], *Liaoningvenator*, MPC-D 100/140 [126]) as well as *Philovenator* [51] and *Linhevenator* [79]. The length of phalanx IV-1 relative to III-1 is similar between *Troodon* (0.59–0.61), *Saurornithoides* (0.62) [58], and other troodontids [56,125,126], but IV-1 is relatively longer in *Linhevenator* (0.88) [79], and *Sinornithoides* (0.71) [81] (Table 2). The dorsoventrally tall and undivided concave proximal articular surface is shared with many other deinonychosaurs [1,3,56,85,98,106,117,125,127]. *Troodon* shares the proximolateral projection of the articulation that is present in *Talos* [56], MPC-D 100/140 [125], *Harenadraco* [78], and *Borogovia* [117]. The triangular ventromedial projection in *Troodon* is similar to the proximoventral heel of *Talos* [56] and MPC-D 100/140 [125]. The depressions on the proximal end of the shaft in *Troodon* are also present in *Talos*. Dorsoventral narrowing prior to the distal articulation in *Troodon* also occurs in *Talos* [56] and to a lesser extent in *Daliansaurus* [85], MPC-D 100/140 [125], and *Sinornithoides* [81]. An extensor fossa is present for this element in *Troodon*, MPC-D 100/140 [125], and *Borogovia* [118], but is absent in *Talos* [56] and *Linhevenator* [79]. Ventromedial and ventrolateral scars are present in *Talos* [56] and MPC-D 100/140 [125] as in *Troodon*. The taller medial distal articulation for *Troodon* is shared with *Talos* [56]*, Borogovia* [118], and MPC-D 100/140 [125]. *Troodon*, MPC-D 100/140 [125], and *Talos* [56] all have a larger medial collateral ligament pit. The non-ginglymoid distal articulation of IV-1 in *Troodon* is shared with MPC-D 100/140 [125], but contrasts with in the ginglymoid surface *Hypnovenator* [116], dromaeaosaurids [98,106], and unenlagiines (e.g., *Neuquenraptor* [127])

### Pes IV-2 and IV-3

Pes phalanxes IV-2 and particularly IV-3 (Fig 30) are poorly represented in the Two Medicine material. IV-2 is represented by MOR 430, 563, and a poorly preserved element from MOR 748. The specimens of IV-3 include left and right from the small juvenile MOR 430 and a poorly preserved left specimen from MOR 748. Since there is no significant differences in morphology between IV-2 and IV–3, they will be described together. Both phalanx IV-2 and IV-3 are short elements, with IV-2 being about 89–98% the length of IV-1 (Table 2). These phalanxes are relatively narrow in the juvenile MOR 430, although this is in part accentuated by crushing. These elements become more robust through ontogeny (S1 File). The proximal articular surface has a subtriangular outline with a straight to slightly concave ventral edge and slightly convex medial and lateral sides. A tongue-like proximodorsal projection from the apex and a wider proximoventral projection create a concave surface when viewed laterally. Although the surface lacks a distinct midline ridge, it is slightly convex transversely. In larger specimens, a rough texture is present just dorsal and ventral to the proximal articulation. Proximoventrally, two medial and ventral bumps develop through ontogeny as well. From the relatively deep proximal articulation, the shaft tapers rapidly. Only a very faint extensor pit lies proximal to the distal articulation. The medial collateral ligament pit is much more developed than the lateral. As in IV-1, III-1, and III-2, the distal articulation is deeper medially. The distal articular surface lacks a discrete groove but is more concave than in phalanx IV-1. As in phalanx III-1, the distal end of younger, smaller specimens (MOR 430) tends to be more concave than in adult specimens. Ventrally, the proximal limit of the articular surface terminates in nearly a straight line.

### Pes IV-2 and IV-3 comparisons

The stoutness of IV-2 and IV-3 in *Troodon* is consistent with *Talos* [56], but unlike the relatively more slender elements of *Philovenator* [52], *Harenadraco* [78], *Sinornithoides* [81], *Daliansaurus* [85], and MPC-D 100/140 [125] and the stouter elements of *Borogovia* [117]. The range of variation in relative length of IV-2 to IV-1 for *Troodon* (0.89–0.98) encompasses the known values for many troodontids [1,52,56,58,84,85,117,125] (Table 2). However, the IV-2 and IV-3 for *Linhevenator* are much shorter by comparison (0.51) [79]. The sub-triangular outline of the proximal articular surface in *Troodon* is consistent with MPC-D 100/140 [125] and *Talos* [56]. The proximodorsal and proximoventral projections in *Troodon* are also present in *Borogovia* [118], *Mei* [1], *Daliansaurus* [85], MPC-D 100/140 [125], and *Talos* [56]. Both *Troodon* and MPC-D 100/140 lack a dorsoventral ridge on the proximal articular surface [125], unlike dromaeosaurids (e.g., *Shri devi* [98], *Deinonychus* [106]) and unenlagiines [127,128]. *Troodon* shares with MPC-D 100/140 [125] and *Borogovia* [118] the faint extensor pit just proximal to the distal articulation, unlike *Talos* [56] and *Linhevenator* [79] where this feature is absent. The shape of the distal articulation and collateral ligament pits in *Troodon* is consistent with that of *Talos* [56] and MPC-D 100/140 [125]. By contrast, IV-2 and IV-3 of *Philovenator* [52], dromaeosaurids such as *Shri devi* and *Deinonychus* [98,106], and unenlagiines [127,128] are more ginglymoid.

### Pes IV-4

Three specimens of IV-4 (Fig 30) come from MOR 553 and one comes from MOR 563. No Two Medicine specimens of phalanx IV-4 come from articulated skeletons. Sides were determined from comparison to figures of *Borogovia gracilicrus* [117] and of fits between various *Troodon* elements. The description and comparison of pes phalanx IV-4 is based on MOR 553s-7-28-92-95, unless noted. Phalanx IV-4 is the most elongate phalanx of digit IV, and is about 72–85% the length of phalanx IV-1 (Table 2). Based on the small juvenile, MOR 430, and the large juvenile, MOR 563, IV-4 exceeds IV-3 in length and is slightly shorter than IV-2. The proximal half of IV-4 is very similar in form to those of the preceding IV-2 and IV-3. Distally, IV-4 is most similar to III-3. As in the latter, the collateral ligament pits sit more dorsally on the sides of the shaft and have a relatively elongate shape, particularly the lateral one. The asymmetric distal articulation is ginglymoid and divided by a deep medial groove. The lateral condyle is slightly shorter dorsoventrally and extends farther distally than the medial one. As in III-3, the articular surface does not extend far proximally on the dorsal aspect of the bone while the ventral proximal extension remains strongly divided.

### Pes IV-4 comparisons

Relative to the length of IV-1, the length of IV-4 in *Troodon* (0.72–0.85) is similar to many other troodontids (0.71–0.89) [52,56,84,85,117,125]. By contrast, this element is relatively short in *Mei* (0.62) [1] and *Linhevenator* (0.49) [79] (Table 2). IV-4 is longer than IV-3 in *Troodon* as well as *Sinornithoides* [84], *Borogovia* [117], *Talos* [56], and *Linhevenator* [79], whereas the condition is reversed in *Mei* [1] and *Daliansaurus* [85]. The proximal half of IV-4 in *Troodon* is very similar to MPC-D 100/140 [125]. When viewed qualitatively, the robustness of IV-4 for *Troodon* is similar to *Talos* [56] but is greater than that of *Sinornithoides* [81] and MPC-D 100/140 [125]. *Borogovia* exceeds the robustness of *Troodon* for this element [117]. The undivided proximal and ginglymoid distal articulations and collateral ligament pits of *Troodon* are consistent with that of *Talos* [56], *Harenadraco* [78], and MPC-D 100/140 [125]. The proximal and distal articulations are ginglymoid in *Deinonychus* [106] and *Neuquenraptor* [127].

### Ungual I

Four examples of ungual I-2 (Fig 24) are known coming from MOR 553 and 748. The description and comparisons of ungual I-2 applies to all of these specimens. Ungual I-2 for *Troodon* is an asymmetric, slightly recurved claw. Throughout its length it maintains a laterally compressed cross-section eventually tapering down to a sharp point. The outline of the ginglymoid proximal articulation is asymmetric with a flatter lateral and more convex medial edge. Only a faint blood groove is present medially. The better expressed lateral groove divides proximally, defining a raised, roughly textured and triangular bump just distal to the articular surface. Additional attachment scarring without any clearly developed tubercle occurs proximoventrally.

### Ungual II

**Fig 31:**
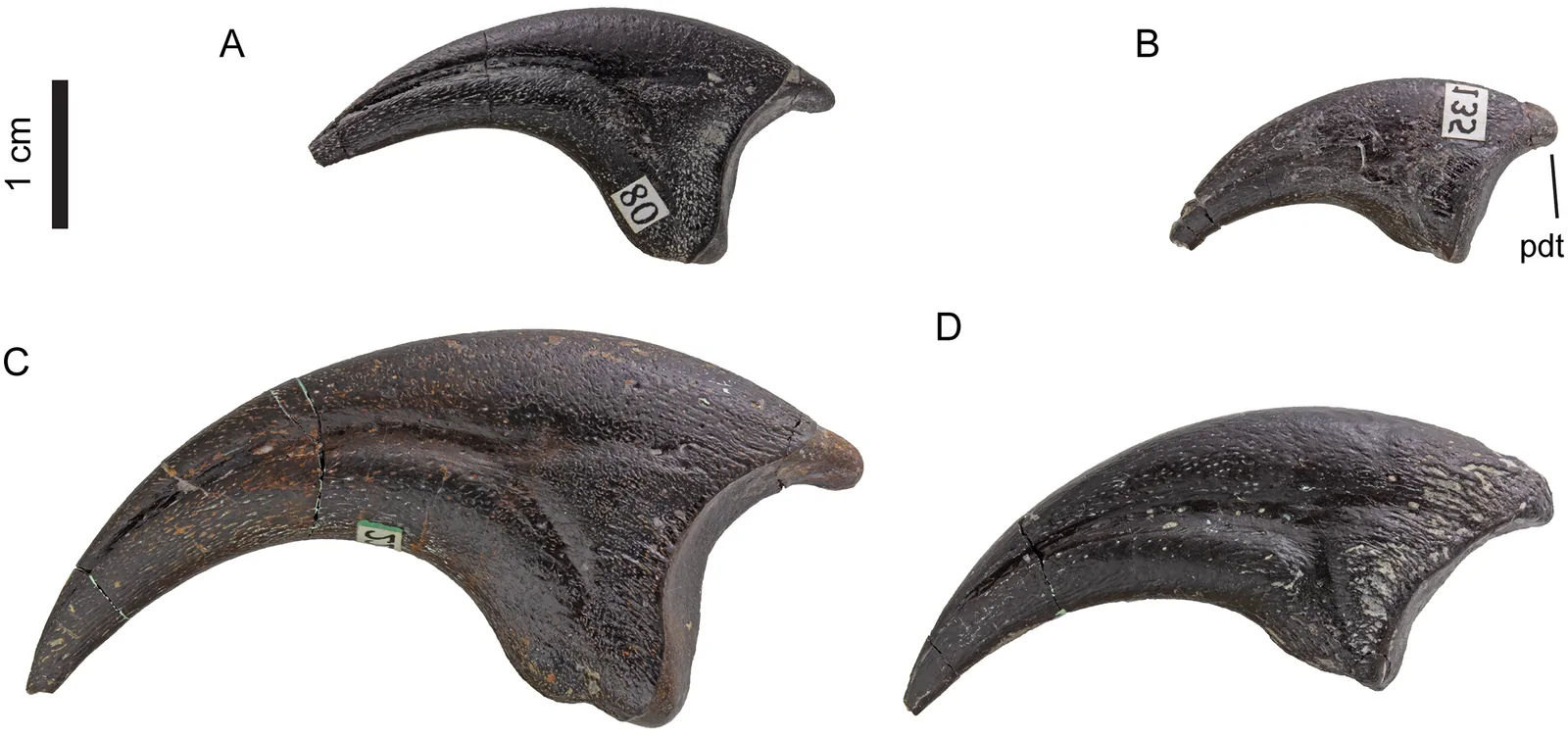
Pedal ungual ontogeny of *Troodon formosus.* Lateral view of juvenile unguals: left pedal ungual II-3 (MOR 553s-7-25-92-80) (A), pedal ungual III-4 or IV-5 (MOR 553s-7-21-91-135, reversed) (B). Lateral view of adult unguals: right pedal ungual II-3 (MOR 553s-8-12-92-221, reversed) (C), pedal ungual III-4 or IV-5 (MOR 553s.8.2.91.292) (D). Abbreviation: pdt, posterodorsal tongue.

The sample for ungual II-3 (Fig 26) consists of eight specimens (five from MOR 553, one from MOR 563, and two from MOR 748). The description and comparisons of ungual II-3 applies to all of these specimens, unless otherwise stated. The enlarged and nearly symmetrical ungual II-3 is strongly laterally compressed and recurved. The proximal articulation remains roughly 2.5 times taller than wide throughout ontogeny (Fig 31). This ginglymoid articulation incorporates a proximally projecting dorsal extension and a smoothly round ventral terminus. On both sides, the blood grooves divide proximally outlining a raised triangle with attachment scarring marking the proximal side. The lateral grooves and triangle are somewhat more pronounced than those of the medial side. A ventral flexor tubercle sits a short distance distally from the articular surface. This feature is not well expressed in the smallest specimens (MOR 563, MOR 553s-8-5-92-164).

### Ungual III or IV

A third type of ungual, distinct from those of digit I and II can be recognized from the Two Medicine sample. Although these include associated material, none come from articulated specimens. It is assumed that these more asymmetrical claws represent digits III and IV (Fig 28). This sample includes four specimens, three from MOR 553 and one from MOR 563. The description and comparisons of this type of ungual applies to all of these specimens unless otherwise stated.

The unguals of digits III and IV differ from those of the inner toes in several ways. First, they are broader with a more triangular cross-section and flatter flexor surface throughout their length. Further, the proximal articulation is not ginglymoid. The articular surface is irregularly smooth and concave, especially in the larger specimens, and lacks a dorsoventral ridge. This seems unusual given the very pronounced ginglymoid distal articulation on the penultimate phalanxes. The outline of the proximal articular surface of the two smallest specimens, MOR 563 and MOR 553s-7-21-91-135, incorporates the small, proximally extending dorsal tongue (Fig 31). This feature is not present in the two larger specimens. Overall, these unguals are as recurved as II-3 and more so than I-2.

### Ungual Comparisons

Overall, the general curvature of the pedal unguals, with the extremely recurved II-3, in *Troodon* is comparable to that of other troodontids [2,3,52,56,79,81,86,88,120,124,125]. This is unlike unguals II-3 and III-4 in *Borogovia* [117] and ungual III-4 in *Harenadraco* [78], which are relatively straight. The curvature and relative sizes of ungual II-3 are much more extreme in dromaeosaurids [36,97,98,106,110], unenlagiines [114,127], and *Rahonavis* [92]. The ginglymoid proximal articulations of I-2 and II-3 in *Troodon* are widespread throughout Deinonychosauria [56,81,98,106,117,125,127]. The lack of a ginglymoid articular surface in III-4 and IV-5 for *Troodon* is shared with MPC-D 100/140 [125] and *Borogovia* [117], which also have ginglymoid distal articulations on the penultimate phalanxes of digits III and IV. This contrasts with the ginglymoid articular surfaces in *Deinonychus* [106]. The flexor tubercle of II-3 in *Troodon*, *Borogovia* [118], and *Hesperornithoides* [2] sits slightly distal to the articular surface. This tubercle is more continuous with the articular surface in *Talos* [56], *Linhevenator* [79], *Philovenator* [51], *Dalianasaurus* [85], *Sinornithoides* [81], and MPC-D 100/140 [125]. The flexor tubercle of II-3 is not as prominent in *Troodon* as it is in *Harenadraco* [78].

## Discussion

Since the initial description of *Troodon formosus* based on a single tooth from the Judith River area of Montana [9], the species has undergone a variety of systematic and taxonomic revisions [6,8,57,75]. The topic of the validity of *Troodon* has remained problematic in part due to the historically fragmentary material assigned to this taxon and the designation of synonymous (e.g., *Stenonychosaurus* [57]) and potentially synonymous (*‘L. mcmasterae’* [8]) taxa. Nevertheless, the name is still in common use [15–21] and begs for a solid taxonomic foundation. We treat MOR 553 as representing *Troodon formosus,* but recognize this assignment is ultimately pending an ICZN decision.

The clade Troodontinae as a whole has remained relatively enigmatic with the vast majority of taxa being known from singular specimens (e.g., [3,56,85,125]). Importantly, only small bodied non-troodontine troodontids (e.g., *Mei* [1], *Sinovenator* [129], *Anchiornis* [82]) are well-represented by multiple articulated skeletons. This lack of additional samples for troodontines has made assessments of functional morphology, individual variation (e.g., ontogeny, sexual dimorphism) and taxonomy challenging. Because the Two Medicine *Troodon* specimens described here represent multiple individuals over a well-preserved ontogenetic growth series, we can make more detailed comparisons to other potentially synonymous taxa, understand the dynamic of troodontid ontogeny and variation, and begin to make interpretations about functional morphology and behavior.

### Comparisons to *Stenonychosaurus* and *‘L. mcmasterae’*

The pelvic and hindlimb material described here for the Two Medicine *Troodon* sample helps clarify some of the anatomical uncertainties surrounding troodontines from the Two Medicine Formation and contemporaneous deposits (e.g., Dinosaur Park, Oldman, and Judith River Formations). The Two Medicine *Troodon* material is largely indistinguishable from the specimens assigned to *Stenonychosaurus* [6,8,57]. Notably, both the holotype for *Stenonychosaurus* (CMN 8539) and all the preserved Two Medicine specimens possess a metatarsal III with a flat to convex anterior face, which distinguishes both taxa from the material assigned to *‘L. mcmasterae’* with a concave anterior face of metatarsal III (TMP 1992.036.0575, TMP 1997.133.0008). Additionally, the shape of the distal extensor fossa of metatarsal III ranges from triangular to ovoid for the Two Medicine sample, which seems to vary ontogenetically. This contrasts with what is reported by van der Reest and Currie [8] for the Two Medicine sample, who also suggest that an ovoid extensor fossa distinguishes *‘L. mcmasterae’* from *Stenonychosaurus*. The pubis material assigned to *‘L. mcmasterae’* (UALVP 55804) is notably more curved than the Two Medicine *Troodon* pubes (MOR 246–11, MOR 430, MOR 553s-8-3-9-387), which possess nearly straight shafts. Additionally, the pubis and ilium in the *‘L. mcmasterae’* specimen are fully fused (co-ossified), unlike any of the material from the Two Medicine sample. Whether or not these differences are taxonomically informative or if the *‘L. mcmasterae’* material represents a later ontogenetic stage than the Two Medicine material requires further analysis with additional fossil material.

### Ontogenetic trends

Overall element morphology remains relatively consistent across the size classes of *Troodon* specimens reported here. For example, the major landmarks of elements (e.g., greater and lesser trochanters of the femur, extensor fossae on the pedal phalanges) are observable across all ontogenetic stages, including some of the embryonic (MOR 246−11) material. The morphologic differences that can be observed across the ontogenetic series are largely related to the relative proportions of these osteologic features, not their complete presence or absence. For instance, while both the posteromedial flange of the fourth metatarsal and the anterior process of the pubic boot become more expansive in larger individuals, the absolute presence or absence of these features does not change.

As is expected, the robusticity of some individual skeletal elements increases with increasing body size. Metatarsal IV, the proximal end of pedal phalanx IV-2, and the distal end of metatarsal III become relatively wider in larger individuals. Other skeletal elements exhibit near isometry in certain aspects, including the femur, tibia, and various phalanges. Relative proportions of skeletal elements in associated specimens also change through growth, with younger individuals typically possessing more elongate distal limb elements. The femur/tibia length ratio, for example, increases from ~0.72 in the small juvenile MOR 430 to ~0.89 in the adult MOR 748. Similarly, the femur/metatarsal IV length ratio increased from ~1.0 in the embryonic MOR 246−11 to ~1.5 in the adult MOR 748. The relative shortening of the distal limb elements through ontogeny in *Troodon* is likely related to allometric scaling is a trend that is consistent with other theropods of differing body sizes, including troodontids [1,38,130].

Surficial bone texture changes can be observed across the ontogenetic series for *Troodon*, a trend that has been documented in many dinosaurs [131–133], including birds [134]. In immature individuals, overall bone texture is typically more porous (e.g., Fig 29), and some articular surfaces are smoother (e.g., Fig 8) than those of mature individuals. As reported in Caldwell et al. [71], the only definitive skeletal fusion (co-ossification) event in the pelvis and hindlimb for this sample is that between the astragalus and calcaneum in the largest, and presumably most mature, specimens in the dataset. MOR 748 possibly exhibits fusion of the distal tarsals to the right metatarsus. If the fusion of other pelvic and hindlimb elements do occur for *Troodon*, they occur even later in ontogeny than the specimens described here. A pelvis attributed to ‘*L. mcmasterae’* (UALVP 55804) does exhibit fusion between the ilium and pubis [8], though it is considerably larger than the largest pubis described here (MOR 553s-8-3-9-387). This delayed fusion of appendicular elements in *Troodon* is in contrast to other potentially immature troodontid specimens such as *Almas* (IGM 100/1323, fused proximal tarsals) [54] and *Philovenator* (IVPP V10597, partially fused tibiotarsus) [51,52]. Variation in the pattern and timing of skeletal fusion across species has been reported for other theropod groups, including oviraptorids [135,136], dromaeosaurids [32,137], and birds [138]. This variation is observable among extant archosaurs as well. For example, a caudo-cranial pattern of vertebral neurocentral suture closure appears to be widespread throughout Pseudosuchia [139,140]. By contrast, the timing of scapulocoracoid fusion is highly variable among extant crocodilians [141]. Additionally, sutures do not necessarily increase in co-ossification through growth. Bailleul et al. [142] documents increasing degrees of cranial suture fusion in the emu (*Dromaius novaehollandiae*) through ontogeny, whereas crocodilians exhibited larger widths in some cranial sutures through growth. The potential for variation in skeletal fusion patterns, even among closely related taxa, highlights the need for caution when using fusion as an indicator of maturity [143].

The overall ontogenetic changes that are observable in the *Troodon* pelvic and hindlimb sample occur at consistent growth stages. This apparently low amount of ontogenetic plasticity for *Troodon* aligns with observations of other paravian dinosaurs, but in contrast to other dinosaur groups (e.g., coelophysoids) [133]. The overall invariance in ontogenetic patterns for *Troodon* supports the use of these characters as a reliable tool for ontogenetic assessment of this taxon and potentially its close relatives. However, the known variability in the timing of these ontogenetic traits among troodontids, and the small sample sizes for these taxa, highlights the need for caution when using the absolute patterns in *Troodon* as a baseline for interpreting the maturity in other taxa. A deeper analysis of the ontogenetic patterns of paravian dinosaurs with the inclusion of a phylogenetically broad array of taxa is needed to understand how reliable ontogenetic assessments can be made.

MOR 430 (the small juvenile specimen) does exhibit some differences from the larger *Troodon* specimens. The relative proportions of the anterior and posterior ends of the pubic boot in MOR 430 are opposite that of the large MOR 553 specimen. Additionally, MOR 430 also lacks the paired distal ridges on the femur that are present in larger specimens. Given that MOR 430 (and MOR 246) were collected stratigraphically lower in the Two Medicine Formation than the larger specimens [60], it is possible these differences are taxonomically significant. Whether these specimens reflect multiple species is the subject of further investigation.

The observed ontogenetic trends in the Two Medicine *Troodon* sample help frame how taxonomic identifications of other troodontids can be made. Though some changes in skeletal proportions, surface texture, and fusion do occur, the presence or absence of most major osteologic features is largely unchanged through growth. In Asia, multiple distinct troodontid taxa have been identified from the same formation and in some cases the same locality [78]. This includes holotype specimens of potentially distinct ontogenetic stages (e.g., *Linhevenator* [79] and *Philovenator* [51], *Byronosaurus* [144] and *Almas* [54]). If some of these co-occurring taxa were actually synonymous, then it would require the complete development or loss of osteologic features through growth and changes in skeletal proportions that are more extreme than what is observable in the *Troodon* growth series. For example, for the type specimen of *Philovenator curriei* (IVPP V 10597) to represent a juvenile individual of *Linhevenator tani* would require drastic developmental changes such as the reduction in the length of the metatarsus relative to the femur. Similarly, if the *Byronosaurus jaffei* [144] and *Almas ukhaa* [54] were the same taxon, changes in the morphology of the cnemial crest of the tibia that are not observable in *Troodon* would need to occur. Hence, our observations of *Troodon* ontogeny would support the validity of these taxa. Whether or not the ontogenetic characteristics of *Troodon* can be used as a baseline for interpreting other taxa is a hypothesis that can only be tested with a broader taxonomic analysis.

### Variation

Although there is overall similarity in ontogenetic patterns amongst the sample, there is some evidence of different growth trajectories amongst the material represented. MOR 748, for example, exhibits numerous characteristics associated with a skeletally/sexually mature individual. This includes being discovered closely associated with an egg clutch [25], histologic characteristics of the bone [47], fused sacral centra and proximal tarsals [71], relatively smooth bone surface texture, and robusticity of some of the pedal phalanges. Some phalanges of similar size to MOR 748 (e.g., MOR 553s-7-7-91-20) are slightly more gracile and exhibit a spongey surface texture, a trait that can be indicative of skeletal immaturity [134]. The pedal phalanges of MOR 748 are more similar to relatively larger specimens such as MOR 553l-11-1-01-14, which also exhibit a relatively smooth bone surface texture. Similarly, other hindlimb elements such as the femora and tibiae have been collected from MOR 553 are considerably larger than the corresponding elements for MOR 748. For example, MOR 553l-7-24-8-64 is a tibia that is about 20% longer than that of MOR 748. It is possible that this variation reflects size (and potentially sexual) dimorphism in *Troodon*, where some individuals attain larger adult body sizes than others. Histologic analyses do suggest divergent growth trends amongst the Two Medicine sample [69]. Similarly, Garros et al. [48] found evidence for two different growth trajectories for troodontid metatarsals from the Dinosaur Park Formation, although it is not clear whether these differences are attributable to intraspecific variation, taxonomy, or pathology. Additional research and sampling is needed to assess the extent of these differences in growth.

### Pathology

A total of eight pedal elements from the Two Medicine sample (3 metatarsals, 5 phalanges) exhibit possible pathologies (Table 3). Individually, these pathologic specimens make up about 20–30% of the sample for their respective elements when excluding embryonic and juvenile material. The most dramatic pathology is an extremely rugose II-1 (MOR 553l-7-25-9-313), given that the offset between shaft and distal end, may have been broken and before healing. Pedal pathologies have frequently been reported for troodontids [48,56,81]. By contrast, pedal pathologies are much less frequently observed in similarly sized theropods [48]. Even among dromaeosaurids, with a similarly enlarged and retractable digit II ungual, identifications of pedal pathologies in the literature are seemingly absent. The greater abundance of pedal pathologies in troodontids compared to dromaeosaurids could be informative of the differences in ecology and/or functional morphology between these two groups. Understanding the diversity in types and causes of the pathologies in this sample and among other troodontids is beyond the scope of this paper, but further study could expand upon the unique behavioral inferences that have been made for troodontids in recent years [23,24,28,30,70].

**Table 3:** List of pathologic *Troodon* elements.

Specimen	Element	Potential Pathology
MOR 748	Metatarsal I	Small medial concavity on shaft.
MOR 553s-8-17-92-260	Metatarsal IV	~2 cm long ovoid rise on shaft.
MOR 553s-11-1-01-8	Metatarsal IV	Low, irregularly textured ridge on lateral side.
MOR 553s-7-6-91-13	Pes II-1	Roughly textured rise dorsal to proximal articulation
MOR 553l-7-25-9-313	Pes II-1	Extensive pathologic remodeling.
MOR 553s-8-11-92-209	Pes III-1	Shallow groove on proximal articulation.
MOR 553s-8-9-92-190	Pes IV-1	Irregular grooves and depressions on proximal articulation.
MOR 553s-8-11-92-213	Pes IV-1	Irregular grooves and depressions on proximal articulation.

Compiled list of pathologic and potentially pathologic *Troodon* hindlimb elements from the Two Medicine Formation sample.

### Functional Morphology

There is a small body of research highlighting the changes in biomechanical properties of the pelvis and hindlimb throughout the evolution of Troodontidae [39]. *Troodon* is one of the largest troodontids known from relatively complete skeletal material and provides a unique opportunity to study the functional morphology of the hindlimb at this body size. The anatomical features suggested to be associated with cursoriality in other theropods (arctometatarsalian condition [42], reduced fibula [41], non-ginglymoid pedal articulations [43,44]) are also observable in *Troodon*. These features are not uniformly present amongst troodontids, with earlier taxa possessing a metatarsus that is not fully arctometatarsalian [116] and more ginglymoid pedal phalanges than *Troodon* [125]. The gradual acquisition of these traits through the evolution of Troodontidae possibly corresponds to changes in locomotor strategies and increased cursorial abilities in later taxa.

Despite possessing a suite of features typically associated with cursoriality, the large size of *Troodon* may have had some influence on its locomotor abilities. Based on the adult specimen, MOR 748, *Troodon* has a relatively short tibia (tibia/femur: 1.12) and metatarsus (metatarsus/femur: 0.66) compared to other troodontids such as *Sinornithoides* (tibia/femur: ~ 1.4, metatarsus/femur: 0.79) [57] and *Gobivenator* (metatarsus/femur: 0.83) [23]. This disparity is potentially a result of allometric scaling between these smaller forms and the larger *Troodon* [85]. The ratios for *Troodon* are comparable or exceed those of the similarly-sized dromaeosaurid *Deinonychus antirrhopus* (tibia/femur: ~ 1.1, metatarsus/femur: 0.45) [113] and smaller-bodied dromaeosaurids such as *Shri devi* (tibia/femur: 1.20, metatarsus/femur: ~ 0.57) [24] and *Velociraptor mongoliensis* (tibia/femur: 1.08, metatarsus/femur: 0.42) [91] which all have notably short metatarsi. Carrano [26] suggested dromaeosaurids to be less capable of cursorial locomotion. The unique combination of “cursorial” and possibly “non-cursorial” traits in *Troodon* suggest it had a distinct locomotor strategy from those of both dromaeosaurids and more diminutive troodontids. However, more work is needed to understand the degree to which each of these morphologic traits played a role in the biomechanical abilities of *Troodon*. This is important to consider given the continuum of cursoriality that exists among modern vertebrates [26] and valuable for understanding the ecologic role of large-bodied troodontids in Late Cretaceous ecosystems.

## Conclusions

The hindlimb and pelvic material described here represent an important first step in documenting the anatomy, variation, and ontogeny in *Troodon formosus*. The ontogenetic trends observed in this sample show that the majority of the osteologic features of the hindlimb are present throughout growth. Additionally, morphologic variability among some of the skeletal remains suggests possible distinct adult sizes within the species, potentially reflecting individual or sexual variation. Finally, the abundance of pedal pathologies amongst the *Troodon* sample adds to the growing body of data highlighting the high frequency of pedal pathologies amongst troodontids. This fossil material also has the potential to provide more nuanced insights into troodontine biomechanics.

## Supporting information

S1 File Measurements and regressions of pelvic and hindlimb elements from *Troodon formosus.* (DOCX)
