# Supplementary material for: Pelvic and hindlimb osteology and ontogeny of a troodontid from the upper member of the Two Medicine Formation (Cretaceous) of Montana
Source: PLoS One. 2026 Sep 9;21(9):e0356249. doi: 10.1371/journal.pone.0356249 (PMC13557401; doi:10.1371/journal.pone.0356249)
Supplement: Supplementary file 1 [file pone.0356249.s001.docx]

| Element | Measurement | Logarithmic equation | Adjusted-R^2^ | 95% confidence interval of scaling coefficient | Allometric trend |
| --- | --- | --- | --- | --- | --- |
| Femur | Length vs Midshaft width | y= 0.038x^1.154^ | 0.9898 | 1.031 – 1.277 | Positive allometry |
| Tibia | Length vs Midshaft width | y= 0.035x^1.123^ | 0.9597 | 0.931 – 1.315 | Isometric |
| Astragalus | Condyle depth vs Condyle width | y= 0.990x^1.128^ | 0.8363 | 0.520 – 1.737 | Isometric |
| Metatarsal III | Distal articulation depth vs Width | y= 0.262x^1.356^ | 0.9749 | 1.201 – 1.512 | Positive allometry |
| Metatarsal IV | Length vs Midshaft width | y= 0.005x^1.605^ | 0.9924 | 1.431 – 1.780 | Positive allometry |
| Pes Phalanx II-1 | Length vs Midshaft width | y = 0.023x^1.627^ | 0.5896 | 0.048 – 3.207 | Isometric |
| Pes Phalanx III-1 | Length vs Midshaft width | y= 0.160x^1.130^ | 0.8887 | 0.639 – 1.620 | Isometric |
| Pes Phalanx III-2 | Length vs Midshaft width | y= 0.445x^0.893^ | 0.9565 | 0.424 – 1.363 | Isometric |
| Pes Phalanx IV-1 | Length vs Proximal width | y= 0.527x^1.026^ | 0.9699 | 0.802 – 1.249 | Isometric |
| Pes Phalanx IV-2 | Length vs Proximal width | y= 0.100x^1.578^ | 0.9973 | 1.375 – 1.780 | Positive allometry |

**Supplementary Table 1:** Regressions of selected element dimensions through ontogeny for *Troodon*. All measurements in millimeters

| Specimen | | MOR 246 (#11) | MOR 430 | MOR 748 |
| --- | --- | --- | --- | --- |
| Side | | R | R | L |
| Length | | ~28 | - | - |
| Post-acetabular length (posterior to ischial peduncle) | | 10.4 | - | - |
| Height (dorsal margin of acetabulum to dorsal margin of ilium) | | ~8 | - | - |
| Anteroposterior length of acetabulum | | 7.6 | 16.6 | 57.5 |
| Exterior of anteroposterior length of acetabular region | | 12.3 | >33.5 | 89.9 |
| Estimated | Illium length | - | 104 | 262 |
|  | Illium height | - | 30 | 77 |

**Supplementary Table 2:** Measurements for ilia from Two Medicine *Troodon* specimens. Abbreviations: L, left; R, right. All measurements in millimeters (mm).

| Specimen | MOR 430 | | MOR 748 | | MOR 553 | |
| --- | --- | --- | --- | --- | --- | --- |
|  |  |  |  |  | 7.27.8.87 | 8.5.9.392 |
| Side | L | R | L | R | R | R |
| PD length | - | - | - | - | 200 | - |
| Width across peduncles | - | - | - | - | 60 | 58.4 |
| Width across obturator process | - | - | - | - | 64.2 | - |
| Obturator process apex to iliac peduncle | - | - | - | - | 140 | - |
| Maximum shaft thickness | 2.7 | 2.5 | - | 8.5 | 10.3 | 10 |

**Supplementary Table 3:** Measurements for ischia from Two Medicine *Troodon* specimens. Abbreviations: L, left; PD, proximodorsal, R, right. All measurements in millimeters (mm).

| Specimen | MOR 246 | MOR 553s | | MOR 748 | MOR 430 | |
| --- | --- | --- | --- | --- | --- | --- |
|  | #11 | 8.3.9.387 | 6.28.9.62 |  |  |  |
| Side | R | R | R | R | L** | R |
| Length | - | 217* | - | - | 103 | - |
| AP proximal | - | 77 | 39 | - | ~18^+^ | - |
| ML proximal | - | 25.7 | 13.4 | - | 3.2 | - |
| AP Iliac articulation | - | ~56 | 24.7 | - | - | - |
| AP Iliac articulation | - |  | 13.4 | - | - | - |
| AP acetabular region | - | 32.5 | 15.6 | - | - | - |
| ML acetabular region | - | 25 | 10.2 | - | - | - |
| DV ischiac process | - | 22 | 11 | - | 11 | - |
| ML ischiac process | - | 21 | 7.6 | - | 4.2 | - |
| Narrowest AP Shaft Dimension | 1 | 14.5 | - | - | ~5 | 4.5 |
| Narrowest ML Shaft Dimension | - | 11.3 | 6.5 | - | 3.7 | 3.3 |
| AP pubic boot | - | 64.4 | - | - | 6 | - |
| ML pubic boot | - | 28.5 | - | - | 4.5 | - |
| Anterior length of boot | - | ~35 | - | - | ~2 | - |
| Posterior length of boot | - | 13.6 | - | - | ~5 | - |

**Supplementary Table 4:** Measurements for pubes from Two Medicine *Troodon* specimens. Abbreviations: AP, anteroposterior; DV, dorsoventral; L, left; ML, mediolateral; R, right; ~ approximate measurement; *probably GL should be ~260; ^+^incomplete (minimum measurement); **this assumes boot goes to left side. All measurements in millimeters (mm).

| Specimen | MOR 246 | | MOR 430 | MOR 563 | | MOR 748 | | MOR 553s | | | | |
| --- | --- | --- | --- | --- | --- | --- | --- | --- | --- | --- | --- | --- |
|  | #11 | |  | Shorter frag. | Longer frag. |  |  | 7.28.91.234 | 8.7.9.417 | 7.22.91.164 | 7.28.91.239 | 7.16.0.61 |
| Side | R | L | R | R | L | R | L | R | L | L | L | L |
| Length | 32.7 | 34.9 | 126 | - | - | 320 | - | 218 | 234 | 238 | 277 | 305^U^  330^E^ |
| ML proximal | - | - | 12* | - | - | 55.1^U^ | 56.6^U^ | 44.5 | 34.1* | 42.3 | 60.2 | 70.7 |
| AP Proximal | 6.1 | 6.7 | 22 | - | - | 56.7 | 51.8^U^ | 32.5^U^ | 31.7^U^ | 31.6 | 49 | 55.7^U^ |
| ML midshaft | 2.3 | 2.3 | 6.3* | - | 15.5* | 28.1 | - | 20 | 17.7 | 19.7 | 26.4 | 33.4 |
| AP midshaft | 3.4 | 3.0 | 11.9 | - | 18.8 | 28.8 | - | 18.8* | 19.3 | 19.2* | 26.2 | 34.9 |
| ML Distal | - | 6.0 | 12.6* | - | - | ~53^U^ | 59.8 | 45.7 | - | 40.5^U^ | 57.2 | 71.7 |
| AP Distal | - | 6.0 | 21.8* | - | - | 45.8^U^ | 43^U^ | 30.2 | - | 26.4^U^ | 42.6 | 64.5 |
| Midshaft C | 8.2^E^ | - | 30 | - | 55 | 89 | - | 61 | 58 | 61 | 83.5 | 104 |

**Supplementary Table 5:** Measurements for femora from Two Medicine *Troodon* specimens. Abbreviations: AP, anteroposterior; C, circumference; DV, dorsoventral; L, left; ML, mediolateral; R, right; ~ approximate measurement; * crushed; ^U^ underestimate; ^E^Estimate. All measurements in millimeters (mm).

| Specimen | MOR 246 | | | MOR 430 | | MOR 563 | MOR 553s | | | | |
| --- | --- | --- | --- | --- | --- | --- | --- | --- | --- | --- | --- |
|  | #1 | #11 | #11 |  |  |  | 7.20.91.132 | 7.17.0.74 | 7.11.91.41 | 8.16.92.254 | 7.28.91.237 |
| Side |  | R | L | R | L | L | L | L | R | L | L |
| Length | - | - | 46.7 | - | ~175 | - | 278 | 320 | 375 | 361 | - |
| Length with astragalus | - | - | - | - | ~180 | - | - | - | - | - | - |
| AP proximal | ~5.3 | 5.8 | - | - | 13* | - | ~45 | 31* | 58 | ~50 | - |
| ML proximal | ~5.7 | ~4.5 | 5.4 | - | 18.7* | - | 33 | 39 | 44 | 51 | - |
| AP at cnemial crest | - | 6.6 | - | - | - | - | 45 | 46 | 62 | 59 | - |
| Height of astragular scar | - | - | - | 49 | - | - | 85 | 99 | 116 | 115 | - |
| Minimum ML midshaft | 3.0 | 2.9 | 3.0 | 7.5 | 7.9 | 18.1 | 19.3 | 23 | 29 | 28 | ~34 |
| AP distal | - | - | - | 7.5 | - | - | 14.7 | 14.6 | 21 | 19 | - |
| ML distal | - | - | 5.7 | 16.2 | 14.9 | - | 41.3 | 44 | 57 | 58 | - |

**Supplementary Table 6:** Measurements for tibiae from Two Medicine *Troodon* specimens (part 1). Abbreviations: AP, anteroposterior; DV, dorsoventral; L, left; ML, mediolateral; R, right; ~ approximate measurement; *incomplete. All measurements in millimeters (mm).

| Specimen | MOR 553e | | MOR 553l | MOR 748 | |
| --- | --- | --- | --- | --- | --- |
|  |  |  | 7.24.8.64 |  |  |
| Side | R | L | R | R | L |
| Length | 323 | - | 430 | 360 | - |
| Length with Astragalus | - | - | - | 367 | - |
| AP Proximal | 42 | 50 | ~51 | 53 | - |
| ML Proximal | 32 | 43 | 52 | ~44 | - |
| AP at cnemial crest | 41 | - | 55 | 57 | - |
| Height of astragalar scar | 92 | - | 130 | 104* | 106 |
| Minimum ML midshaft | 23 | - | 33 | 27 | 27 |
| AP distal | ~11 | 17 | 17 | ~20 | 22 |
| ML distal | ~45 | 60 | 62 | 50 | 50 |

**Supplementary Table 7:** Measurements for tibiae from Two Medicine *Troodon* specimens (part 2). Abbreviations: AP, anteroposterior; DV, dorsoventral; L, left; ML, mediolateral; R, right; ~ approximate measurement; *incomplete. All measurements in millimeters (mm).

| Specimen | MOR 430 | MOR 748 | MOR 553s  7.18.0.90B | MOR 553s  8.17.92.265 | MOR 246 #1 |
| --- | --- | --- | --- | --- | --- |
| Side |  |  |  |  |  |
| AP proximal | ~16 | >37 | 35.6 | 46 | 5.9 |
| ML proximal | 3 | ~11 | 7.1 | 15 | 1.4 |
| Least shaft diameter |  |  | 2.5* | 4.2* |  |

**Supplementary Table 8:** Measurements for fibulae from Two Medicine *Troodon* specimens. Abbreviations: AP, anteroposterior; L, left; ML, mediolateral; R, right; ~ approximate measurement; >minimum measurement; *likely too large given lack of distal end. All measurements in millimeters (mm).

| Specimen | MOR 430 | | MOR 553 | | | MOR 553s | | MOR 748 |
| --- | --- | --- | --- | --- | --- | --- | --- | --- |
|  |  |  | 11.1.01.3 | 11.1.01.2 | 11.1.01.1 | 7.7.91.19 | 8.20.92.311 |  |
| Side | R | L | L | L | L | R | R | R |
| ML width | 14.4 | 14.6^+^ | - | - | 55.7 | 36.7 | 40.2 | 48.8 |
| AP depth | 11.9 | 11.3 | 26.6 | ~30 | ~30 | 20.9 | 22.4 | ~28 |
| PD medial condyle | 9.9 | 9.3 | 20.1 | 26.4 | 25.8 | 17.1 | 17.9 | 23.4 |
| PD lateral condyle | 10.7 | 11.5 | - | - | 32.3 | 19.6 | 19.9 | 27.8 |
| Height of astragulus w/ascending process preserved | 32.1 (I) | 56* | - | 51 (I) | - | 76 (I) | 58 (I) | 87.8 (I)  ~107* estimated |

**Supplementary Table 9:** Measurements for astragali from Two Medicine *Troodon* specimens. Abbreviations: AP, anteroposterior; L, left; ML, mediolateral; PD, proximodistal; R, right; ~ approximate measurement; * complete; (I) incomplete; ^+^measurement including calcaneum. All measurements in millimeters (mm).

| Specimen | Side | Completeness | PD height | AP width | ML width |
| --- | --- | --- | --- | --- | --- |
| MOR 430 | L | Complete | 11.8 mm | ~8mm | <1 mm |
| MOR 748 | R | Incomplete | ~27 mm | ~23 | - |
| MOR 553 11.1.01.1 | L | Most complete | 31.9 | 24.2 | ~6.2 |

**Supplementary Table 10:** Measurements for calcanea from Two Medicine *Troodon* specimens. Abbreviations: AP, anteroposterior; L, left; ML, mediolateral; PD, proximodistal; R, right; ~ approximate measurement; * complete; (I) incomplete; ^+^measurement including calcaneum. All measurements in millimeters (mm).

Distal Tarsal

| Specimen | MOR 430 | MOR 553s | | |
| --- | --- | --- | --- | --- |
|  |  | 7.16.91.74 | 11.1.01.4 | 7.28.91.245 |
| Side | R | R |  | L |
| Transverse width | 7.3 | 15.9 | 16.2 | 17.8 |
| AP length | 5.9 | 12.2 | 13.1 | 15.5 |
| DV thickness | 2.1 | 3.9 | 4.3 | 5.8 |

**Supplementary Table 11:** Measurements for distal tarsal IV from Two Medicine *Troodon* specimens. Abbreviations: AP, anteroposterior; DV, dorsoventral; L, left; R, right. All measurements in millimeters (mm).

| Specimen | MOR 748  (poorly preserved) | MOR 553s | |
| --- | --- | --- | --- |
|  |  | 8.2.91.293 | 8.6.92.168 |
| Side | R | L | L |
| Length | 24.5* | 33.3 | 33.1 |
| ML distal | 10.2* | 13.1* | 10.9 |
| PD distal end | 12.0* | 13.9 | 13.0 |
| AP distal | 11.6* | 13.2* | 13.7 |

**Supplementary Table 12:** Measurements for first metatarsals from Two Medicine *Troodon* specimens. Abbreviations: AP, anteroposterior; DV, dorsoventral; L, left; PD, proximodistal; R, right; *incomplete. All measurements in millimeters (mm).

| Specimen | MOR 430 | | MOR 563 | | MOR 553s | | | MOR 553 | MOR 748 | |
| --- | --- | --- | --- | --- | --- | --- | --- | --- | --- | --- |
|  |  |  |  |  | 7.21.91.227 | 7.8.91.28 | 7.18.92.5 | 11.1.01.5 |  |  |
| Side | L | R | L | R | L | R | L | L | L | R |
| Length | - | - | - | - | - | 114.8*  est. 180 | 207 | - | - | 187 |
| AP proximal | 11.5 | - | 15.5^+^ | - | 21.2 | 27.1 | 32.3 | 31 | ~26 | 28.1 |
| ML proximal | 5.6 | - | 11.6 | - | 15.2 | 18.7 | 25.4 | 22.3 | 23.7 | ~19 |
| AP midshaft | 4.8 | 4.8 | - | 8.8 | 9.2 | 10.9 | 15.6 | - | 12.2 | 13.3 |
| ML midshaft | 2.7 | 2.1 | - | 4.9 | 5.0 | 5.5 | 8.3 | - | 7.1 | 7.2 |
| AP distal | - | 4.7 | 14.2 | - | - | 17.7 | 21.4 | 19.3 | - | 18.4 |
| ML distal | - | 5.8 | 9.6 | - | - | 12.6 | 17.2 | 15.6 | - | 16.7 |

**Supplementary Table 13:** Measurements for second metatarsals from Two Medicine *Troodon* specimens. Abbreviations: AP, anteroposterior; DV, dorsoventral; L, left; ML, mediolateral; R, right; ~ approximate measurement; *greatly shortened; ^+^some lost material. All measurements in millimeters (mm).

| Specimen | MOR 430 | | MOR 563 | | MOR 553s | | | MOR 493 | MOR 553 | | | | MOR 748 | |
| --- | --- | --- | --- | --- | --- | --- | --- | --- | --- | --- | --- | --- | --- | --- |
|  |  |  |  |  | 7.29.92.113 | 8.6.9.406 | 7.16.91.79 |  | 11.1.01.6 | 553-1 | 11.1.01.7 | 552-2 |  |  |
| Side | L | R | L | R | L | L | L | L | L | R | R | R | L | R |
| Length | - | - | - | - | - | - | - | - | - | - | - | - | - | 212 |
| ML proximal | - | - | - | - | - | - | - | - | - | - | - | - | - | ~10 |
| ML midshaft | - | - | - | ~2 | - |  |  | - | - | - | - | - | 3.6 | ~4.3 |
| AP midshaft | - | - | - | ~4.7 | - |  |  | - | - | - | - | - | ~10.5 | - |
| ML distal | 5.9 | - | 14.8 | - | 16.1 | 17.4 | 21.9 | 21.5 | 20.8 | 22.7 | 24.3 | 25.7 | - | 20.3 |
| AP distal | 10.6 | - | 18.4 | - | 20.7 | 20.8 | 25.9 | 27.2 |  | 27.6 | 27.9 | 31.4 | - | 24.7 |

**Supplementary Table 14:** Measurements for third metatarsals from Two Medicine *Troodon* specimens. Abbreviations: AP, anteroposterior; L, left; ML, mediolateral; R, right; ~ approximate measurement. All measurements in millimeters (mm).

| Specimen | MOR 246 | MOR 430 | | MOR 563 | | MOR 553 | | MOR 553e | MOR 553s | | MOR 553l | MOR 748 | |
| --- | --- | --- | --- | --- | --- | --- | --- | --- | --- | --- | --- | --- | --- |
|  | #11 |  |  |  |  | 11.1.01.9 | 11.1.01.10 | 7.1.9.9 | 8.17.92.260 | 11.1.01.8 | 7.28.8.102 |  |  |
| Side | L | L | R | L | R | L | R | L | L | R | R | L | R |
| Length | 33.7 | - | ~114* | - | - | - | - | 144.4 | 195 | 240 | 243 | - | 207 |
| ML proximal | - | 8.1 | 7.7 | - | - | - | - | 16.5 | 23.7 | 41.2 | 37.3 | - | 30.1 |
| AP proximal | - | ~16.2 | 19.4 | - | - | - | - | 20.6 | 35.3 | 38.9 | 35.7^+^ | - | 33.0 |
| ML midshaft | 1.9 | - | 6.4 | - | 13.2 | - | - | 10.2 | 15.0 | 22.3 | 21.7 | - | ~17.5 |
| AP midshaft | 2.2 | - | 7.3 | - | 14.5 | - | - | 11.7 | 18.4 | 26.0 | 28.1 | - | 22.6 |
| ML distal | - | 5.0 | - | - | - | 15.6 | 13.0 | 9.0 | 16.5 | 22.0 | 23.5 | - | 18.5 |
| AP distal | - | 9.2 | - | - | - | 21.3 | 20.6 | 14.8 | 21.3 | 28.9 | 27.3 | - | 22.6 |

**Supplementary Table 15:** Measurements for fourth metatarsals from Two Medicine *Troodon* specimens. Abbreviations: AP, anteroposterior; L, left; ML, mediolateral; R, right; ~ approximate measurement; *estimate based on left and right specimens of MOR 430; ^+^ incomplete. All measurements in millimeters (mm).

| Specimen | MOR 430 | MOR 748 | MOR 553s 7.21.92.45 |
| --- | --- | --- | --- |
| Side | R | R | L |
| Length | 9.8 | 23.5 | 28.6 |
| ML proximal | 4.0 | 12.8 | 12.9 |
| DV proximal | 4.7 | 14.4* | 16.1 |
| ML midshaft | 2.8 | 10.9 | 11.3 |
| DV midshaft | 3.2 | 9.6 | 10.9 |
| ML distal | 3.3 | 10.7 | 10.9 |
| DV distal | 4.3 | - | 13.1 |

* missing portions

**Supplementary Table 16:** Measurements for pedal phalanges I-1 from Two Medicine *Troodon* specimens. Abbreviations: DV, dorsoventral; L, left; ML, mediolateral; R, right; *missing portions.

All measurements in millimeters (mm).

| Specimen | MOR 553 | MOR 553s | MOR 748 |
| --- | --- | --- | --- |
|  | 11.1.01.20 | 7.18.91.113 |  |
| Side | R | L | R |
| Length | - | >31.8 | - |
| ML proximal | 7.8 | 9.1 | 9.9 |
| DV proximal | - | 16.4 | 16.4 |

**Supplementary Table 17:** Measurements for pedal unguals I-2 from Two Medicine *Troodon* specimens. Abbreviations: DV, dorsoventral; L, left; ML, mediolateral; R, right; > minimum measurement. All measurements in millimeters (mm).

| Specimen | MOR 430 | MOR 563 | MOR 748 | | MOR 553s | | | | MOR 553l | | MOR 656 |
| --- | --- | --- | --- | --- | --- | --- | --- | --- | --- | --- | --- |
|  |  |  |  |  | 7.20.91.121 | 7.6.9.148 | 6.29.9.89 | 7.6.91.13 | 11.1.01.11 | 7.25.9.313* |  |
| Side | L | R? | R | L | L | L | L | L | L | R | L |
| Length | 18.6 | - | 42.1 | - | 38.2 | 45.4 | 48.5 | 53.5 | - | 48.8 | - |
| ML proximal | 3.7 | 10.1 | 15.6 | 16.0 | 12.1 | 15.1 | 19.2 | 18.9 | - | 16.5 | 14.6 |
| DV proximal | 7.5 | 15.3 | 22.0 | - | 16.2 | 19.3 | - | 24.9 | - | 22.7 | 19.9 |
| ML midshaft | 3.2 | - | 11.7 | 12.8 | 7.8 | 10.3 | 12.8 | 13.2 | - | 15.5 | 10.9 |
| DV midshaft | 5.6 |  | 12.0 | 13.5 | 9.6 | 12.3 | 13.8 | 14.1 | - | 13.7 | 13.1 |
| ML distal | 4.6 |  | 13.4 | - | 11.5 | 14.0 | 16.8 | 15.4 | - | 16.8 | - |
| DV distal | 6.8 | - | 19.5 | - | 15.1 | 19.6 | 21.4 | 23.0 | - | - | - |

**Supplementary Table 18:** Measurements for pedal phalanges II-1 from Two Medicine *Troodon* specimens. Abbreviations: DV, dorsoventral; L, left; ML, mediolateral; R, right; *pathologic specimen. All measurements in millimeters (mm).

| Specimen | MOR 430 | TM-020 | MOR 563 | | MOR 553s | | MOR 553l | MOR 553 |
| --- | --- | --- | --- | --- | --- | --- | --- | --- |
|  |  |  |  |  | 11.1.01.12 | 8.3.92.143 | 7.25.9.318 | 11.1.01.13 |
| Side | L? | R | L | R | R | R | L | L |
| Length | - | 16.2 | 24.1 | - | 34.1 | - | - | 36.3 |
| ML proximal | 2.8 | 5.7 | 7.1 | - | 13.9 | 9.5 | 10.1 | 14.9 |
| DV proximal | - | - | - | - | 24.2 | - | - | - |
| ML distal | 3.8 | - | 8.0 | 7.4 | 12.6 | 8.7 | 11.1 | 12.3 |
| DV distal | 5.6 | 6.6 | 11.3 | 11.5 | 16.7 | 12.4 | - | 17.7 |

**Supplementary Table 19:** Measurements for pedal phalanges II-2 from Two Medicine *Troodon* specimens. Abbreviations: DV, dorsoventral; L, left; ML, mediolateral; R, right. All measurements in millimeters (mm).

| Specimen | MOR 563 | MOR 553 | MOR 553s | | | | MOR 748 | |
| --- | --- | --- | --- | --- | --- | --- | --- | --- |
|  |  | 11.1.01.21 | 7.25.92.80 | 8.5.92.164 | 8.6.92.176 | 8.12.92.221 |  |  |
| Side |  | R | L | R | L | R | L | R |
| Length | >35 | - | >36 | >31 | >40 | >59 | - | - |
| ML proximal | 5.7 | 6.4 | 6.3 | 5.6 | 6.8 | 10.1 | 9.7 | 9.8 |
| DV proximal | 16.0 | - | 15.8 | 14.2 | 17.6 | 24.1 | - | - |

**Supplementary Table 20:** Measurements for pedal unguals II-3 from Two Medicine *Troodon* specimens. Abbreviations: DV, dorsoventral; L, left; ML, mediolateral; R, right; > minimum measurement. All measurements in millimeters (mm).

| Specimen | MOR 430 | | MOR 563 | | MOR 553 | MOR 553s | | MOR 748 | MOR 553l |
| --- | --- | --- | --- | --- | --- | --- | --- | --- | --- |
|  |  |  |  |  | 11.1.01.17 | 8.11.92.209 | 7.7.91.20 |  | 11.1.01.14 |
| Side | L | R | L | R | L | L | R | L | R |
| Length | - | 20.8 | 39.4 | - | - | 53.8 | 48.9 | 49.2 | 57.6 |
| ML proximal | - | 9.0 | 14.6 | - | - | 25.6 | 21.7 | - | 29.2 |
| DV proximal | - | 8.4 | 16.7 | - | - | 25.4 | 22.4 | >24 | 25.2* |
| ML midshaft | - | 5.4 | 7.9* | - | - | 14.9 | 12.7 | 14.1 | 17.2 |
| DV midshaft | - | 5.4 | 9.3 | - | - | 14.6 | 12.6 | 12.4 | - |
| ML distal | 6.7 | 8.4 | 13.4 | 13.9 | 18.3 | 22.5 | 19.6 | 20.6 | 25.3 |
| DV distal | 6.7 | 6.6 | 11.9 | 11.5 | 15.2 | 17.1 | 15.4 | 16.1 | 19.0 |

**Supplementary Table 21:** Measurements for pedal phalanges III-1 from Two Medicine *Troodon* specimens. Abbreviations: DV, dorsoventral; L, left; ML, mediolateral; R, right; > minimum measurement; * crushed. All measurements in millimeters (mm).

| Specimen | MOR 430 | MOR 563 | MOR 553l | MOR 553s | MOR 748 |
| --- | --- | --- | --- | --- | --- |
|  |  |  | 7.31.8.110 | 7.23.91.177 |  |
| Side | R | L | L | L | L |
| Length | 16.8 | 29.0 | 32.7 | 33.0 | - |
| ML proximal | 7.7 | 12.8 | 16.1 | 15.8 | - |
| DV proximal | 6.4* | 13.5 | 15.7 | 15.9 | - |
| ML midshaft | 5.6 | 8.4 | 10.1 | 10.6 | 13.8 |
| DV midshaft | 3.4* | 7.0 | 9.0 | 8.6 | 10.0 |
| ML distal | 6.8 | 11.3 | 13.5 | 14.1 | 16.8 |
| DV distal | 5.0 | 9.0 | 11.6 | 12.0 | 13.4 |

**Supplementary Table 22:** Measurements for pedal phalanges III-2 from Two Medicine *Troodon* specimens. Abbreviations: DV, dorsoventral; L, left; ML, mediolateral; R, right; * crushed. All measurements in millimeters (mm).

| Specimen | MOR 430 | MOR 563 | MOR 553s | MOR 748 |
| --- | --- | --- | --- | --- |
|  |  |  | 8.2.91.292 |  |
| Side | L | L | L | L |
| Length | 14.8 | 26.9 | 36.8 | - |
| ML proximal | 6.2 | 10.2 | 16.2 | - |
| DV proximal | 5.1 | 11.5 | 16.3 | - |
| ML midshaft | 4.3 | 7.2 | 11.8 | 12.4 |
| DV midshaft | 2.7* | 5.8 | 9.2 | 8.8 |
| ML distal | 5.5 | 9.9 | 14.3 | - |
| DV distal | 4.5 | 9.5 | 14.3 | - |

**Supplementary Table 23:** Measurements for pedal phalanges III-3 from Two Medicine *Troodon* specimens. Abbreviations: DV, dorsoventral; L, left; ML, mediolateral; * crushed. All measurements in millimeters (mm).

| Specimen | MOR 563 | MOR 553s | | |
| --- | --- | --- | --- | --- |
|  |  | 7.21.91.135 | 8.19.92.285 | 8.2.9.353 |
| Length | >27 | >28 | >46.5 | >40 |
| ML proximal | 6.7 | 7.4 | 12.0 | 12.0 |
| DV proximal | 13.2 | 12.4 | 18.5 | 20.5 |

**Supplementary Table 24:** Measurements for pedal unguals III-4/IV-5 from Two Medicine *Troodon* specimens. Abbreviations: DV, dorsoventral; L, left; ML, mediolateral; R, right; > minimum measurement. All measurements in millimeters (mm).

| Specimen | MOR 430 | MOR 553 | MOR 563 | MOR 553s | | | |
| --- | --- | --- | --- | --- | --- | --- | --- |
|  |  | 11.1.01.18 |  | 7.8.9.160 | 8.2.92.190 | 7.24.91.190 | 8.11.92.213 |
| Side | R | R | L | L | L | L | R |
| Length | 12.7 | 21.8 | 22.8 | 32.8 | 34.8 | 34.9 | 35.9 |
| ML proximal | 7.6 | 11.2 | 12.4 | - | 20.4 | 21.5 | 20.5 |
| DV proximal | 6.2 | 14.0 | 15.0 | - | 23.2 | 23.9 | 23.2 |
| ML distal | 7.1 | 11.5 | 12.0 | - | 19.0 | 19.2 | 19.3 |
| DV distal | 5.5 | 10.7 | 11.6 | - | 17.8 | 17.8 | 18.1 |

**Supplementary Table 25:** Measurements for pedal phalanges IV-1 from Two Medicine *Troodon* specimens. Abbreviations: DV, dorsoventral; L, left; ML, mediolateral; R, right. All measurements in millimeters (mm).

| Element | Phalanx IV-2 | | | | | Phalanx IV-3 | | |
| --- | --- | --- | --- | --- | --- | --- | --- | --- |
| Specimen | MOR 430 | MOR 563 | MOR 748 | | TM-088 (no MOR Number) | MOR 430 | | MOR 748 |
| Side | L | L | L | R |  | L | R | L |
| Length | 12.4 | 20.3 | 24.6 | 24.2 | 25.0 | 10.5 | 10.6 | ~20 |
| ML proximal | 5.4 | 11.3 | - | 15.4 | 16.5 | 4.2 | 5.0 | - |
| DV proximal | 7.0 | - | 16.7 | 15.3 | 17.2 | 6.5 | 6.5 | - |
| ML distal | 4.6 | 10.4 | - | 15.3 | 14.6 | 4.5 | 4.2 | - |
| DV distal | 6.0 | 9.9 | 12.3 | - | 12.5 | 5.0 | 5.3 | - |

**Supplementary Table 26:** Measurements for pedal phalanges IV-2 and IV-3 from Two Medicine *Troodon* specimens. Abbreviations: DV, dorsoventral; L, left; ML, mediolateral; R, right; ~ approximate measurements. All measurements in millimeters (mm).

| Specimen | MOR 553s | MOR 553 | MOR 563 | MOR 553s |
| --- | --- | --- | --- | --- |
|  | 7.28.92.95 | 7.8.9.161 |  | 7.11.91.44 |
| Side | L | R | R | L |
| Length | 14.2 | 14.7 | 19.3 | 21.4 |
| ML proximal | 6.3 | 6.5 | 8.0 | 10.0 |
| DV proximal | 7.6 | 7.6 | 9.8 | 11.5 |
| ML distal | 6.0 | 6.3 | 8.6 | 9.9 |
| DV distal | 6.1 | 6.2 | 8.0 | 9.8 |

**Supplementary Table 27:** Measurements for pedal phalanges IV-4 from Two Medicine *Troodon* specimens. Abbreviations: DV, dorsoventral; L, left; ML, mediolateral; R, right. All measurements in millimeters (mm).
